# Heavy metals: toxicity and human health effects

**DOI:** 10.1007/s00204-024-03903-2

**Published:** 2024-11-20

**Authors:** Klaudia Jomova, Suliman Y. Alomar, Eugenie Nepovimova, Kamil Kuca, Marian Valko

**Affiliations:** 1 Department of Chemistry, Faculty of Natural Sciences, Constantine The Philosopher University in Nitra, 949 74 Nitra, Slovakia; 2 Doping Research Chair, Zoology Department, College of Science, King Saud University, 11451 Riyadh, Saudi Arabia; 3 Department of Chemistry, Faculty of Sciences, University of Hradec Kralove, 50005 Hradec Kralove, Czech Republic; 4 Center of Advanced Innovation Technologies, VSB-Technical University of Ostrava, 708 00 Ostrava-Poruba, Czech Republic; 5 Biomedical Research Center, University Hospital Hradec Kralove, Hradec Kralove, Czech Republic; 6 Faculty of Chemical and Food Technology, Slovak University of Technology, 812 37 Bratislava, Slovakia

**Keywords:** Heavy metals, Toxicity, Oxidative stress, ROS, Human disease, Antioxidant enzymes

## Abstract

Heavy metals are naturally occurring components of the Earth’s crust and persistent environmental pollutants. Human exposure to heavy metals occurs via various pathways, including inhalation of air/dust particles, ingesting contaminated water or soil, or through the food chain. Their bioaccumulation may lead to diverse toxic effects affecting different body tissues and organ systems. The toxicity of heavy metals depends on the properties of the given metal, dose, route, duration of exposure (acute or chronic), and  extent of bioaccumulation. The detrimental impacts of heavy metals on human health are largely linked to their capacity to interfere with antioxidant defense mechanisms, primarily through their interaction with intracellular glutathione (GSH) or sulfhydryl groups (R-SH) of antioxidant enzymes such as superoxide dismutase (SOD), catalase, glutathione peroxidase (GPx), glutathione reductase (GR), and other enzyme systems. Although arsenic (As) is believed to bind directly to critical thiols, alternative hydrogen peroxide production processes have also been postulated. Heavy metals are known to interfere with signaling pathways and affect a variety of cellular processes, including cell growth, proliferation, survival, metabolism, and apoptosis. For example, cadmium can affect the BLC-2 family of proteins involved in mitochondrial death via the overexpression of antiapoptotic Bcl-2 and the suppression of proapoptotic (BAX, BAK) mechanisms, thus increasing the resistance of various cells to undergo malignant transformation. Nuclear factor erythroid 2-related factor 2 (Nrf2) is an important regulator of antioxidant enzymes, the level of oxidative stress, and cellular resistance to oxidants and has been shown to act as a double-edged sword in response to arsenic-induced oxidative stress. Another mechanism of significant health threats and heavy metal (e.g., Pb) toxicity involves the substitution of essential metals (e.g., calcium (Ca), copper (Cu), and iron (Fe)) with structurally similar heavy metals (e.g., cadmium (Cd) and lead (Pb)) in the metal-binding sites of proteins. Displaced essential redox metals (copper, iron, manganese) from their natural metal-binding sites can catalyze the decomposition of hydrogen peroxide via the Fenton reaction and generate damaging ROS such as hydroxyl radicals, causing damage to lipids, proteins, and DNA. Conversely, some heavy metals, such as cadmium, can suppress the synthesis of nitric oxide radical (NO^·^), manifested by altered vasorelaxation and, consequently, blood pressure regulation. Pb-induced oxidative stress has been shown to be indirectly responsible for the depletion of nitric oxide due to its interaction with superoxide radical (O_2_^·−^), resulting in the formation of a potent biological oxidant, peroxynitrite (ONOO^−^). This review comprehensively discusses the mechanisms of heavy metal toxicity and their health effects. Aluminum (Al), cadmium (Cd), arsenic (As), mercury (Hg), lead (Pb), and chromium (Cr) and their roles in the development of gastrointestinal, pulmonary, kidney, reproductive, neurodegenerative (Alzheimer’s and Parkinson’s diseases), cardiovascular, and cancer (e.g. renal, lung, skin, stomach) diseases are discussed. A short account is devoted to the detoxification of heavy metals by chelation via the use of ethylenediaminetetraacetic acid **(**EDTA), dimercaprol (BAL), 2,3-dimercaptosuccinic acid (DMSA), 2,3-dimercapto-1-propane sulfonic acid (DMPS), and penicillamine chelators.

## Introduction

The term “environment” refers to the conditions surrounding an organism or group of organisms, particularly the assortment of external physical factors that impact and influence an organism’s ability to grow, develop, and survive (Duruibe et al. 2007). It encompasses aquatic, terrestrial, and atmospheric habitats as well as flora, animals, and abiotic elements. The most tangible elements of the environment, such as air, water, and food, as well as the less physical but no less significant ones, such as the communities in which we live, are taken into consideration (Gore 1997). Any substance found in the environment whose effects may have adverse health effects, lower quality of life, harm the environment's welfare, or even potentially result in death is considered a pollutant. Such a substance usually exists in the environment beyond an exposure limit that is established by health and safety authorities (Occupational Safety and Health Administration).

The term “heavy metal” refers to a metallic element that has a relatively high density (> 4 g/cm^3^ or 5 times greater than water) and is dangerous or poisonous even at low concentrations (Hawkes 1997). Heavy metals (the most toxic being cadmium, lead, arsenic, and mercury) are an integral part of the environment in which we live since they cannot be broken down or eliminated. Heavy metals are naturally occurring components of the Earth's crust, are persistent environmental pollutants, and have many unfavorable effects on ecosystems (Rice et al. 2014).

Heavy metals are found in the biosphere, including rocks, soils, and water, and originate from a variety of sources, such as mining, industrial effluents, urban runoff, sewage discharge, soil erosion, natural weathering of the Earth’s crust, pesticides, disease control agents used on crops, metal pipes for water, traffic, combustion byproducts from coal-burning plants and many others.

Toxic metal particles emitted into the atmosphere from mines gradually settle in floodplains and riverbank soils, where they may remain for hundreds or even thousands of years. Millions of people worldwide in North and South America, Asia, and other parts of the world reside in floodplains where hazardous waste from current and former metal mining operations may be present in dangerously high concentrations (Macklin et al. 2023).

The toxicity of these pollutants is a growing concern due to ecological, nutritional, and environmental factors. Heavy metals such as arsenic, cadmium, chromium, copper, lead, nickel, and zinc are the most frequently detected heavy metals in wastewater and pose a threat to both human health and the environment (Lambert and Leven 2000).

Humans are also exposed to heavy metals through industrial operations such as foundries, smelters, oil refineries, petrochemical plants, pesticide manufacturing, and the chemical industry (Mitra et al. 2022). Industrial applications for arsenic include the glass, textile, paper, and wood industries, and it is also employed as an alloying agent (Balali-Mood et al. 2021). Aluminum is used in many industries because of its beneficial properties, generally in an alloyed form. Many industries use the hexavalent chromium employed in chromate painting, welding, and electroplating. The majority of heavy metal exposures at work are related to metal working and machining, including but not limited to work done in foundries, fabrication, smelting, welding, grinding, refinishing, and repairing. The application of toxicology concepts and methods to chemical and biological risks that arise at work due to heavy metal exposure is known as occupational toxicology.

Heavy metals can enter the body through several means, such as the intake of polluted food or liquids (water), inhalation of contaminated air, and dermal absorption, and may negatively affect a wide range of biological processes (Tchounwou et al. 2012). The risks associated with heavy metals usually outweigh the advantages. For example, even though trivalent chromium is beneficial to health, hexavalent chromium has been identified as a carcinogen, and if inhaled, it may cause lung cancer (Sun et al. 2015). Lead poisoning has been linked to intellectual impairments, predominantly in infants (Hou et al. 2013). In addition to their potential to cause harm to other parts of the human body, heavy metals often induce toxicity to the kidneys, brain, liver, skin, and heart. Even at lower exposure levels, these metallic elements are known to cause damage to numerous organs and are classified as systemic toxicants. Both the International Agency for Research on Cancer and the U.S. Environmental Protection Agency classify several carcinogens as human carcinogens (known or probable).

Heavy metals in biological systems may gain an extra advantage because of their ability to coordinate with various chemical targets and undergo oxidation–reduction reactions, which allows them to evade control mechanisms such as transport, homeostasis, compartmentalization, cell signaling, and antioxidant systems. By displacing essential metals from their natural binding sites, heavy metals may bind to these protein regions, leading to cellular dysfunction and eventual poisoning (Valko et al. 2005). In addition, the consequences of heavy metal binding to proteins include the Fenton reaction-mediated generation of ROS and oxidative stress, which in turn may cause the oxidative degradation of biological macromolecules, DNA damage, and other detrimental effects.

**Fig. 1:**
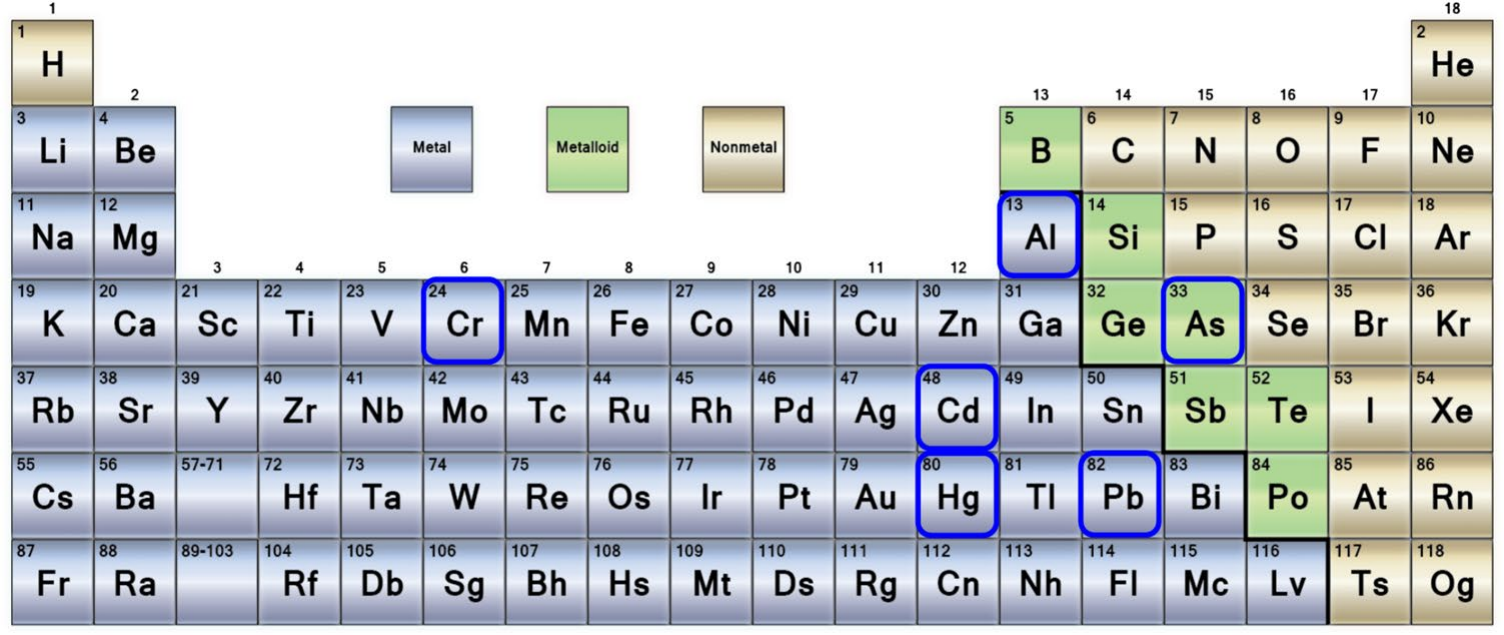
Periodic table of elements showing the marked metals that are the subject of this review. With courtesy from: https://sciencenotes.org/printable-periodic-table/

This review aims to discuss the mechanism of toxicity of aluminum, cadmium, arsenic, mercury, lead, and chromium (Fig. 1). In addition, the health effects of heavy metals on organ systems are comprehensively reviewed.

## Aluminum

Aluminum (Al) is the 13th element in the periodic table and the third most abundant element (8.2% by weight) in the Earth’s crust. The electronic configuration of aluminum is 1s^2^2s^2^2p⁶3s^2^3p^1^ = [Ne]3s^2^3p^1^. Al has a relatively low density and silver sheen. It has various industrial applications in chemical and food processing, medication, and the construction industry (Brough and Jouhara 2020).

The ionization energies of all three electrons on the valence shell (3s^2^3p^1^) are significantly lower than the fourth ionization energy. Aluminum as a metal is zerovalent; however, aluminum compounds can exist in three oxidation states, + 1, + 2, and + 3, with the latter being the most stable (Housecroft 2018). Al has a strong affinity toward oxygen and forms aluminum oxides on the surface, thus forming a protective coating on the materials.

### Aluminum in the human environment

Aluminum-based compounds are present in various products and processes, such as oil refining, photography, and building. These compounds include aluminum nitrates, chlorides, phosphates, sulfates, silicates, and hydroxides (Igbokwe et al. 2019).

Aluminum is present in the air as particles originating from rocks and soil (Sorenson et al. 1974). The major anthropogenic sources of aluminum are coal power plants, steel foundries, the metal refining industry, and car emissions. Inhalation, ingestion, and dermal exposure are the main routes of intoxication by aluminum. Inhaled aluminum enters organisms in the form of particles or droplets. The industrial release of waste containing aluminum to surface waters could be toxic to aquatic life and humans (Gensemer and Playle 1999). Al has the lowest solubility at approximately neutral pH (6.5); under acidic conditions (pH < 5.7), aluminum is present in the form of the trivalent cation Al^3+^ and aluminum hydroxy species Al(OH)^2+^. Foods may contain aluminum naturally or from food additives (Sepe et al. 2001). The possible sources of aluminum in foods originate from the grilling of meat in aluminum foil and the use of aluminum cookware and wrapping. The extent of food contamination by aluminum during the cooking process depends on the cooking temperature, cooking duration, pH, and composition of the food (Valkonen and Aitio 1997).

### Metabolism of aluminum

As outlined above, inhalation and ingestion are the two main routes of contamination of the human body with aluminum. Following inhalation, aluminum dust or (nano)particles are relatively poorly absorbed (approximately 2–3%). The absorption efficiency depends predominantly on the size and chemical form of the Al particles. In the bloodstream, aluminum is chelated by various organic ligands (neutral or anionic) and further distributed to organs, mostly to bones and lungs (Kim et al. 2018). Soluble aluminum compounds do not accumulate in the lungs and are preferentially released into the bloodstream (Riihimäki and Aitio 2012).

Following ingestion, gastrointestinal absorption of aluminum is the main route through which this metal systemically accumulates in humans and other living species. It has been reported that the absorption of Al is associated with a disrupted intestinal mucosa barrier (Hao et al. 2022). The absorption process of Al compounds strongly depends on the structure, size, and nature of the ligands that are coordinated with aluminum and the pH of the environment. It has been reported that Al coordinated with organic ligands such as flavonoids and phytates from food inhibits aluminum absorption (Reto et al. 2007). Conversely, Al absorption is enhanced in the presence of citrate, lactate, maltol, fluoride, and other ligands.

More than 90% of an aluminum load is bound to a transport protein—albumin—and an iron-binding glycoprotein—transferrin. Thus, the absorption of aluminum interferes with the disturbed homeostasis of iron. In individuals with iron overload (e.g., hemochromatosis), the absorption of aluminum is significantly inhibited.

Although aluminum has no physiological relevance, its structural characteristics, such as ionic radius (0.54 A) and oxidation number (+ 3), make it a competitive inhibitor of several essential metals with similar structural characteristics, such as calcium (ionic radius = 0.99 A and oxidation number + 2) and magnesium (ionic radius = 0.72 Å and oxidation number + 2). Under physiological conditions, aluminum occurs in the form of Al(OH)_3_ and is barely soluble in aqueous environments. Physically healthy subjects absorb less than 0.5% of orally administered aluminum through the gastrointestinal tract (Yokel and McNamara 2001). The intestinal absorption of aluminum can be facilitated by the fatty acids present in food (Aspenstrom-Fagerlund et al. 2009). Al is efficiently eliminated from the human body by the kidneys (Bernardo and Edwards 2023). Aluminum can accumulate in the human body in sufficient quantities only when the gastrointestinal tract is bypassed or when there is significant renal dysfunction.

### Mechanisms of aluminum toxicity

Aluminum toxicity manifests as diverse effects capable of causing a wide range of systemic pathological conditions (aluminum toxicosis). The toxic effects of aluminum are related to aluminum-related pro-oxidant effects, which in turn cause oxidative stress-induced damage to proteins and membrane lipids (Exley 2013).

#### Aluminum and neurotoxicity

Perhaps the most critical target of aluminum toxicity is the brain. Aluminum is eliminated from the human body slowly, with a half-life of ca. 7 years; therefore, long-term cumulative damage to neurons may have severe pathological consequences (Drago et al. 2008).

Experimental research indicates that aluminum compounds may be neurotoxic because they may produce damaging ROS, which may have an impact on several organelles, including nuclei, lysosomes, and mitochondria (Pasha and Oglu 2017; Wu et al. 2012). Al-induced oxidative stress in mitochondria results in a decrease in the mitochondrial membrane potential, which is essential for the normal functioning of neuronal cells (Rahimzadeh et al. 2022).

The pathophysiology of neurodegenerative diseases such as Alzheimer’s disease and their association with Al^3+^ toxicity have been reported (de Sautu et al. 2018). Like amyloid-β, a neuropathological hallmark associated with Alzheimer’s disease (Mujika et al. 2018), aluminum may interact with the oxygen atoms of amino acids and the protein backbone, resulting in protein denaturation and structural alterations. Similarly, aluminum has been shown to trigger aggregation and precipitation of the protein amyloid-β (Ricchelli et al. 2005). Another indirect link between aluminum and Alzheimer's disease is manifested by its interaction with oxygen atom-containing residues of proteolytic enzymes (proteases), resulting in the inhibition of the proteolytic degradation process of amyloid-β and its increased accumulation (Sakamoto et al. 2006).

#### Aluminum and inflammation

Analysis of various tissues revealed that aluminum-induced oxidative stress can further exert proinflammatory effects (Igbokwe et al. 2019). Systemic inflammation is a precursor of various disorders, including neurodegenerative diseases. Exposure to aluminum increases the levels of the chemokine macrophage inflammatory protein-1α (MIP-1α) and pro-inflammatory cytokines such as interleukin-1 (IL-1) and tumor necrosis factor (TNF-α). Cytokines released after aluminum exposure have been shown to recruit leukocytes, which in turn secrete more chemotactic cytokines (chemokines) and proinflammatory cytokines, resulting in the enhancement of the inflammatory process (Milnerowicz et al. 2015).

After the mice were exposed to aluminum sulfate-containing standard food for 0, 1, 3, or 5 months, markers of systemic inflammation were assessed in the blood serum (Pogue et al. 2017). The control group received magnesium sulfate. The aluminum-exposed group of mice presented increased levels of proinflammatory interleukin-6 (IL-6), tumor necrosis factor-α (TNF-α), and acute phase reactive protein C-reactive protein (CRP), which indicates that an aluminum-containing diet contributes to systemic inflammatory conditions, as observed in many pathologies, including neurodegenerative disorders such as Alzheimer’s disease.

#### Aluminum and energy metabolism

Inhibitory effects have been observed for the interaction of aluminum with metabolic and other enzyme systems (Sushma et al. 2007; Zatta et al. 2000). Inhibitory effects have been observed for the interaction of aluminum with the phosphate groups of nucleoside di- and tri-phosphates (such as ADP and ATP), two key players in the energy transduction process. The main parenchymal liver cells, hepatocytes exposed to Al, show suppressed ATP production, impaired function of the tricarboxylic acid cycle (TCA, also known as the Krebs cycle or the citric acid cycle), increased levels of oxidized lipids and proteins, and inhibited glycolysis (Mailloux et al. 2011). These and other aluminum-mediated metabolic disorders may be responsible for the weight loss reported in individuals exposed to aluminum in the metal industry (Cerme et al. 2020).

It has been shown that Al^3+^ is an irreversible inhibitor of plasma membrane Ca^2+^-ATPase and a slowly reversible inhibitor of sarcoplasmic reticulum Ca^2+^-ATPase (de Sautu et al. 2018). Whereas in the sarcoplasmic reticulum, calcium pump binding of Al^3+^ interferes with Ca^2+^ in the plasma membrane, the calcium pump does not. This study revealed the distinct mechanism of Al^3+^ inhibition in two Ca^2+^-ATPases.

An interesting relationship between the exposure of chicks to Al and vitamin D levels has been reported (Dunn et al. 1993). The results revealed that exposure of chicks to AlCl_3_ reduced the tissue levels of vitamin D-dependent calbindin D-28 K, a member of a large family of intracellular calcium-binding proteins. More detailed experiments confirmed that aluminum species, but not chloride anions, are responsible for decreased tissue levels of vitamin D-dependent proteins.

#### Aluminum and renal toxicity

For the first time, aluminum intoxication from tap water was described in the late 1970s in patients in Newcastle (UK) undergoing hemodialysis (Ward et al. 1978). Indeed, aluminum intoxication is usually observed in people who have impaired kidney function. Aluminum intoxication of the renal system is manifested by altered protein synthesis, nucleic acid functions, membrane permeability, and inhibition of enzyme activities. Aluminum-mediated enzyme inhibition may in turn lead to increased ROS formation and consequently oxidative stress. Increased ROS can alter membrane fluidity and the DNA repair process, induce DNA damage, and affect the NF-kB, JNK, and p53 signaling pathways.

In renal tissue, aluminum causes a decrease in the glutathione (GSH) concentration and glutathione peroxidase (GPx), glutathione S-transferase (GST), and catalase (CAT) activities (Mahieu et al. 2009). This results in an increase in lipid peroxidation (LPO). Aluminum accumulation in renal tissue causes renal-tubular cell degeneration, alters cellular metabolism, and increases oxidative stress. These factors then impact p-aminohippuric acid (secreted by the renal tubules) transport and phosphate reabsorption in kidney tubules, cause imbalances in sodium and water, and ultimately result in nephrotoxicity.

#### Aluminum and peroxidation of lipids

Al^3+^ may replace iron in Fe^3+^ sites because of the charge compatibility and structural similarities between the ionic radii of Al^3+^ and Fe^3+^. Therefore, aluminum can be bonded to iron transport proteins. This process may increase the levels of free intracellular Fe^2+^ (a substrate for the Fenton reaction), decrease Fe^2+^ binding, and cause membrane damage via the peroxidation of membrane lipids (El-Sayed et al. 2011).

Al-mediated iron overload results in ROS-induced lipid peroxidation and the apoptosis of erythrocytes, lymphocytes, and osteoblasts (Kell 2009; Niemoeller et al. 2006). Al-induced apoptosis is related to increased expression of the proapoptotic proteins Bax, Bak, and Bim and suppressed expression of the antiapoptotic protein Bcl-2 (Xu et al. 2018).

Analysis of tissue homogenates from rats exposed to aluminum revealed abnormal levels of thiobarbituric acid reactive substances (TBARS) and malondialdehyde (MDA), which are toxic, and oxidative stress markers. Compared with normal levels, MDA enhances the release of proinflammatory cytokines such as tumor necrosis factor (TNF-α) several times. In addition, aluminum has been shown to decrease the levels of antioxidant enzymes such as SOD, catalase, and glutathione peroxidase and the highly abundant cytoplasmic antioxidant glutathione (GSH) (Anane and Creppy 2001).

#### Aluminum and developmental toxicity

The toxic effects of aluminum have the potential to negatively affect the development and growth of various organs. Similarly, prenatal exposure to aluminum and the development and morphology of the gerbil prostate have been studied (Gomes et al. 2019). The results revealed that intrauterine exposure of gerbils to aluminum caused permanent changes in the development and morphology of both male and female gerbil prostates. These data suggest that the altered modulation of androgen and estrogen prostate receptors causes aluminum-induced endocrine disruption in gerbils.

### Health effects of aluminum toxicity

Aluminum has been implicated in the pathogenesis of various disorders affecting the functions of the nervous system, renal system, lungs, gastrointestinal tract, cardiovascular system, and other organ systems.

#### Aluminum and pulmonary effects

Workers exposed to aluminum compounds have been shown to have deficiencies in pulmonary function tests. These tests include forced vital capacity (FVC), forced vital capacity (FPV), forced mid-expiratory flow rate (FEF%), and various other tests that help to diagnose a wide spectrum of respiratory disorders with significant structural and functional alterations (Rahimzadeh et al. 2022). This is because aluminum is produced as poisonous dust that leads to the development of lung cancer, pulmonary fibrosis, pulmonary granulomatosis, pulmonary alveolitis, alveolar proteinosis, asthma, chronic bronchitis, chronic pneumonia, and chronic obstructive pulmonary disease (COPD) (Igbokwe et al. 2019).

Exposure to aluminum may also cause asthma (Burge et al. 2000); however, asthma diagnosed among workers in the metallic industry may have a more complex origin, involving other chemical factors such as smoke and inhalation of gases (Taiwo et al. 2006).

Aluminum phosphide is a highly toxic inorganic compound used as a pesticide and in the semiconductor industry. Acute oral exposure to aluminum phosphide causes pulmonary edema; however, the toxic effect has been attributed to the formation of highly toxic phosphine gas rather than to aluminum toxicity (Alter et al. 2001)**.**

Chronic Al exposure in laboratory animals revealed no histological changes in the lungs of rats exposed to 70 mg of aluminum/kg/day in drinking water as AlCl_3_ for 1, 2, or 3 months (Dixon et al. 1979). If laboratory animals are not exposed to aluminum dust or aerosol inhalation routes, observation of pulmonary lesions is rare and inconsistent.

Histological evaluation of the lungs of Wistar rats following aluminum chloride exposure has been reported (Buraimoh and Ojo 2013). The rats received aluminum chloride through oral intubation for 8 weeks. Photomicrographs of the lungs revealed congested blood vessels in the Al-exposed group of rats, confirming the detrimental effect of aluminum chloride on lung histology.

The effects of inhalation exposure to Al fumes on pulmonary function have been investigated through the levels of C-reactive protein (CRP), reflecting the level of inflammation, and alpha-1-antitrypsin (A1AT) protein, whose deficiency reflects a risk of lung damage, particularly chronic obstructive pulmonary disease (COPD) (Elserougy et al. 2015). The results of this study suggest the determination of both markers, CRP and A1AT, which sensitively reflect the level of potential lung damage in workers exposed to aluminum fumes.

#### Aluminum and neurological effects

Although it is highly protected, the brain is vulnerable to aluminum-mediated damage. As mentioned above, the half-life of aluminum in the brain is approximately 7 years; therefore, cumulative damage over such a long period may affect the axonal transport of neurofilaments, neurofilament assembly, and neuronal development, induce oxidative stress, and cause neuroinflammation (Bernardo and Edwards 2023).

An association between aluminum exposure and the incidence of Alzheimer’s disease was reported for the first time in 1965 (Klatzo et al. 1965). The aluminum hypothesis has been suggested on the basis of observations of the effects of aluminum salts on the rabbit brain, which induce cognitive deficits related to the development of neurofibrillary alterations, similar to the neurofibrillary tangles present in the brains of patients with Alzheimer’s disease. Research has not confirmed this hypothesis; currently, only a few papers are devoted to aluminum's role in the etiology of AD every year (Wang et al. 2016).

Glutamate is the major excitatory neurotransmitter in the central nervous system and plays an important role in psychiatric and neurodegenerative pathologies (Wang et al. 2024). It has been suggested that environmental exposure to and dietary intake of heavy metals, including aluminum, can worsen excitotoxicity, which in turn is manifested by enhanced clinical problems associated with autism spectrum disorders (Blaylock and Strunecka 2009).

In association with the need to alleviate symptoms of aluminum-altered excitotoxicity, neuroprotective substances from natural sources may be beneficial. Convolvulus pluricaulis (Convolvulaceae) is a traditional Indian herbal medicine used as a nerve tonic (Bihaqi et al. 2009). The regular administration of Convolvulus pluricaulis (150 mg/kg) together with AlCl_3_ (50 mg/kg) for 3 months suppressed the increased enzymatic activity of acetylcholine esterase and inhibited the aluminum-mediated decline in sodium pump (Na^+^/K^+^-ATPase) activity.

#### Aluminum and hematological effects

Aluminum can interfere with the process of the formation of blood cellular components (hematopoiesis) (Drüeke et al. 1986). The results of an animal study revealed that Wistar rats that received 4 mg/kg aluminum for 3 weeks experienced a disturbed process of hematopoiesis (Chmielnicka et al. 1996). The most sensitive indicator was increased activity of heme oxygenase (HO-1) in the liver and decreased serum iron levels in the rats. Al also increased the liver and kidney activity of delta-aminolevulinic acid synthase (ALA synthase) and decreased rat serum iron levels. Conversely, no alterations in the activity of delta-aminolevulinic acid dehydratase activity (ALA-D) were observed in the livers or kidneys of the rats. These results confirmed that the accumulation of Al in the bone, liver, and spleen may directly affect hematopoiesis. The most sensitive indicators of this process are a decrease in rat serum iron levels and increased heme oxygenase activity in the liver tissues of the rats.

Aluminum has been implicated in the incidence of anemia (Bernardo and Edwards 2023). The factors by which aluminum affects the incidence of anemia involve hindered iron absorption and transport in the serum. In addition, aluminum displaces the binding of iron to transferrin, the major iron transport protein. Patients suffering from aluminum-induced anemia often have decreased corpuscular hemoglobin and increased reticulocyte counts, which measure the number of reticulocytes (immature red blood cells) in the bone marrow.

#### Aluminum and cardiovascular effects

Although the brain is most vulnerable to aluminum-induced damage, the cardiovascular system, including vessels, is also highly susceptible to aluminum toxicity (Tinkov et al. 2024). Epidemiological data from both occupationally and non-occupationally exposed individuals confirmed that exposure to aluminum increases the incidence of a wide range of cardiovascular disorders, the most common of which are atherosclerosis and hypertension. Laboratory studies have reported that increased aluminum body burden is correlated with lipid metabolic disorders such as dyslipidemia and impairment of the renin‒angiotensin‒aldosterone system (RAAS), maintaining a concerted action of hormones and proteins, which in turn regulate blood pressure, blood volume, electrolytic balance, and other important cardiovascular functions. Aluminum toxicity mediates increased ROS levels and, subsequently, oxidative stress, inflammation, and ultimately, cardiomyocyte apoptosis.

Compared with workers in metal-free environments, workers in the metal industry who process aluminum may suffer from atherosclerosis and hypertension (Panev et al. 2019). Aluminum exposure interferes with atherosclerosis through several distinct mechanisms. Aluminum may increase the risk factor for atherosclerosis and dyslipidemia, which are characterized by increased levels of cholesterol, specifically increased low-density lipoprotein-cholesterol (LDL-C, “bad”) and decreased levels of high-density lipoprotein-cholesterol (HDL-C, “good”) (Ghosh et al. 2023). Decreased levels of “good” HDL-C following aluminum exposure are related to the inhibition of paraoxonase-1 (PON1), which is an essential protective enzyme that plays an important role in drug metabolism and the prevention of cardiovascular and neurodegenerative diseases (Taler-Verčič et al. 2020).

One of the most toxic Al-based compounds, aluminum phosphide, has been reported to negatively impact cardiovascular health. Al-phosphide poisoning is responsible for myocardial dysfunction caused by myocarditis, an inflammatory condition of the myocardium (Hangouche et al. 2017). A patient intoxicated with Al-phosphide underwent transthoracic echocardiography, which revealed that myocardial dysfunction was related to global hypokinesis and the presence of a massive left ventricular thrombus. In addition, magnetic resonance confirmed left ventricular systolic dysfunction. Following 6 months of symptomatic treatment, all the cardiac lesions were reversed.

#### Aluminum and gastrointestinal effects

Oral aluminum intoxication can trigger dysfunction of the intestinal barrier, manifested by increased epithelial cell apoptosis, dysregulation of transmembrane proteins responsible for the transport of charged species and solutes, and increased intestinal permeability (Hao et al. 2022). In the gastrointestinal system, aluminum triggers immune responses that lead to the activation of immune cells to secrete many proinflammatory cytokines. In addition, aluminum can negatively affect the bacterial microflora in the gut by inhibiting the growth of probiotic bacteria and supporting the growth of pathogenic bacteria, which in turn causes a systematic inflammatory response.

Aluminum enhances the penetration of lipopolysaccharides across the intestinal mucosal barrier into the blood circulation. Elevated lipopolysaccharides sensitize Kupffer cells to secrete proinflammatory cytokines such as tumor necrosis factor-α (TNF-α), IL-1β, and IL-6, resulting in intestinal endotoxemia, alternatively termed circulating endotoxin (Wang et al. 2017).

The aluminum-mediated inflammatory pathway causes damage to the gut barrier and activates extracellularly regulated protein kinases (EKs) and NF-κB (Jeong et al. 2020). The activated NF-κB pathway can, in turn, increase the expression of matrix metalloproteinase-9 (MMP-9). Overexpressed MMP-9 has been correlated with increased metastatic potential in gastrointestinal cancers (Prathipaa et al. 2021).

The intestinal barrier is vulnerable to oxidative stress-induced damage. Al exposure downregulates the levels of the antioxidant enzymes SOD, catalase, and glutathione peroxidase and the synthesis of the highly abundant intracellular antioxidant glutathione (GSH), which is reflected by increased GSSG/GSH ratios, malondialdehyde levels, and other oxidative stress biomarkers in the small intestine of rats (Eltahawy et al. 2017).

Increased exposure to aluminum may disturb gut homeostasis and contribute to the progression of inflammatory bowel diseases (IBDs), such as Crohn’s disease and ulcerative colitis. Intoxication with aluminum in murine models of colitis has been studied (Pineton de Chambrun et al. 2014). Colitis and chronic colitis in mice are induced by 2,4,6-trinitrobenzene sulfonic acid and dextran sodium sulfate, respectively. Histological examination revealed that aluminum increased the level and duration of inflammation. In addition, aluminum increased the expression of colonic myeloperoxidase, which may serve as a biomarker of inflammation and oxidative stress. This finding was further supported by the increased expression of proinflammatory cytokines and suppressed epithelial cell renewal in the experimental animals compared with those in the control animals.

## Cadmium

Cadmium (Cd) is the 48th element in the periodic table and has physicochemical properties similar to those of mercury and zinc. The electronic configuration of cadmium is [Kr] 4d^10^5s^2^, and the most stable and most common oxidation number of cadmium is + 2. Cadmium is a soft, silvery-white flammable metal with very low solubility in water. Unlike the majority of other metals, cadmium has a shallow susceptibility to corrosion and is, therefore, used to create a protective layer on the surface of more corrosive metals. Every year, more than 13,000 tons of cadmium are produced worldwide for color pigments, Ni–Cd batteries, metal coatings, chemical stabilizers, and alloys.

### Cadmium in the environment

Cadmium is a rare element in the Earth’s crust and originates from the breakdown of rocks caused by physical and chemical forces (weathering) and volcanic action. Anthropogenic sources of cadmium result from the mining and processing of cadmium-containing sulfide ores of copper, zinc, and lead. Areas contaminated with cadmium represent a potential source of cadmium dust. The main route of Cd exposure is through the lungs and partly through the gastrointestinal tract. Many studies have confirmed that the half-life of cadmium in humans is approximately 15–20 years (Valko et al. 2005).

Human activities result in the release of approximately 5000–13000 tons of cadmium per year into the environment. Cadmium is blown into the air by the wind from natural and industrial processes such as the combustion of Cd-containing ores or volcanic activities. The origins of cadmium in water include water supplies close to mines, battery recycling facilities, landfills, hazardous waste sites, the burning of fossil fuels, excessive fertilizer use, waste incineration, and cadmium-using industrial activities. Aquatic organisms can absorb cadmium from contaminated water and accumulate it in their tissues (He et al. 2023). Relatively high amounts of cadmium in phosphorus fertilizers have a strong negative effect on the seed germination of crops. Thus, excessive anthropogenic activities due to the excessive use of chemical fertilizers may increase the concentration of cadmium in the food chain.

### Cadmium metabolism

As outlined above, the main routes of cadmium exposure are the lungs and gastrointestinal tract, two major sites where cadmium is taken up and transported via the blood to the liver and kidney (Nordberg 1984). Several studies across various countries and regions have shown that the concentration of cadmium in the blood of individuals can reach a maximum of 1 µM, most frequently much lower, approximately in the range of 1–10 nM (van Kerkhove et al. 2010).

The amount of cadmium absorbed depends on age, sex, physical status, and other factors. Reproductive-aged women with lower iron can be at risk for increased absorption of cadmium following oral exposure (Olsson et al. 2002). Depending on the particle size and solubility, approximately 10–50% of inhaled cadmium is absorbed. With respect to orally ingested cadmium, only 6% is absorbed, and less than 1% of cadmium is absorbed dermally. Humans excrete absorbed cadmium mostly through urine (ATSDR 2010).

The major portion of Cd that enters the body is transported in the bloodstream via erythrocytes, albumin, and other transport proteins and accumulates in the liver, kidneys, and gut. The kidney and liver are particularly susceptible to cadmium toxicity. As a defense mechanism, the tissues of these organs efficiently synthesize cadmium-inducible metallothionein. This small cysteine-rich protein, discovered in 1957, binds cadmium ions (Margoshes and Vallee 1957) and thus protects cells against Cd-mediated toxicity (Genchi et al. 2020). In addition to toxic metals, metallothionein may also bind essential (zinc, copper, and iron) metals. The coordination environment around the Cd^2+^ ion in metallothionein is tetrahedral with four sulfur donor atoms from -SH groups. Thus, metallothionein provides protection against cadmium and other toxic heavy metals, maintains the homeostasis of essential metals, and suppresses oxidative stress via a ROS-scavenging mechanism (Sabolić et al. 2010). Cadmium in the form of Cd-metallothionein complexes is stored in the liver and consequently redistributed to the kidneys, the main target organ for cadmium toxicity. In contrast to free metals, cadmium coordinated with metallothionein has a significantly lower level of toxicity.

The rather slow elimination rate of cadmium is most likely caused by the fact that cadmium is tightly attached to metallothionein, which is nearly entirely reabsorbed in the renal tubules (Genchi et al. 2020). The elimination routes of cadmium involve the kidneys, urine, and saliva. The urine cadmium concentration most precisely represents the overall body load, whereas the blood cadmium concentration indicates recent exposure. Nevertheless, the excretion rate increases dramatically, and urine cadmium levels no longer represent the body burden when renal impairment due to cadmium exposure occurs.

Long-term cadmium exposure may cause various adverse effects involving kidney tubular disorders, resulting in the dysfunction of channels and transporters of the renal tubular system, resulting in the loss of fluids and a shift in electrolyte homeostasis (Genchi et al. 2020).

Cd^2+^ interferes with the transport system across the cell membrane and epithelia. Cadmium in renal tubules can suppress the reabsorption of Na^+^, K^+^, Mg^2+^, Ca^2+^, Cl^−^, amino acids, glucose, and other transported molecules, which in turn can result in pathological states such as polyuria, hypercalciuria, glucosuria, and other pathologies. Despite the extensive research devoted to elucidating the mechanisms by which Cd^2+^ affects membrane transport, it is still unclear whether Cd^2+^ has a primary or secondary effect on cell membrane transport (Cuypers et al. 2010a, b; van Kerkhove et al. 2010, 2013).

Owing to the structural similarities between cadmium ions and several d-electron essential (transition) metals, such as Fe, Zn, and Cu, cadmium can significantly affect the cellular homeostasis maintained by these essential metals, which may have serious health consequences (Chen et al. 2022a, b).

DMT1—divalent metal (ion) transporter 1 is the major iron transporter and entry point for iron into the body (intestinal brush-border uptake). Iron deficiency has been shown to increase the intestinal absorption of Cd^2+^ through the DMT1 transport system (Park et al. 2002). Research has confirmed that the structural similarity with Fe^2+^ or Cu^2+^ allows Cd^2+^ to cross the biological membrane through channels designed for the active transport of essential metals. These channels involve not only divalent metal transporter 1 (DMT1) but also ZIP transporters, which are responsible for Zn transport into the cytoplasm across the cellular membrane, Ca^2+^-selective transient receptor cation channels, and other transport systems. Zinc reabsorption in kidney renal tubules by ZIP transporters has been shown to be inhibited by cadmium ions (He et al. 2009).

A vast majority of the filtered Ca^2+^ is reabsorbed by the nephron. The Ca^2+^ transport mechanisms may involve apical Ca^2+^ channels (regulated by vitamin D and dietary calcium), basolateral Ca^2+^-ATPase (regulating the large transmembrane gradient of Ca^2+^ ions), and sodium‒calcium (Na^+^-Ca^2+^) exchangers (membrane proteins that remove Ca^2+^ from cells) (Carafoli 1991). Intoxication by cadmium interferes with calcium transport systems and decreases fluid reabsorption, negatively affecting passive Ca^2+^ absorption across the renal proximal tubule and bulk transport of solutes in the ultrafiltrate transported back from the renal tubule by water flux (solvent drag) (Hechtenberg and Beyersmann 1991).

Despite the many valuable experimental models attempting to elucidate the effect of cadmium on transmembrane transport, future work will be needed to emulate the experimental conditions much closer to the in vivo situation. Perhaps the most critical parameter is the concentration of Cd^2+^ applied under in vitro conditions, which is often several orders of magnitude greater than that encountered in vivo.

### Mechanisms of cadmium toxicity

Cadmium is one of the most toxic heavy metals and may exert a wide range of toxic effects via a variety of mutually interconnected mechanisms. The major mechanisms and molecular pathways of cadmium toxicity involve (i) gene regulation, (ii) oxidative stress, (iii) mitochondrial apoptosis, (iv) autophagy, and (v) interactions with essential metals (Dukic-Cosis et al. 2020).

#### Cadmium and gene regulation

Cadmium affects the expression of various genes (Dukic-Cosis et al. 2020).

*Immediate early genes (IEGs):* An immediate early gene is characterized by low-level expression following rapid and transient induction by an extracellular stimulus such as cadmium intoxication. Cadmium intoxication activates c-jun N-terminal kinase (JNK), the most crucial mitogen-activated protein kinase (MAPK) family activated by environmental stressors. Cadmium-induced tumorigenesis is often associated with the increased transcription of the proto-oncogenes c-fos, c-jun, and c-myc (Wang and Templeton 1998).

*Stress response genes:* Studies of Cd-induced carcinogenesis have explored the expression of stress response genes, such as those encoding metallothionein, heat shock proteins (HSPs), and genes involved in the synthesis of glutathione. For example, suppressed expression of metallothionein has been related to susceptibility to cadmium-induced toxicity and carcinogenicity.

*Transcription factors involved in cancer development:* Cadmium can interfere with the activity of transcription factors involved in development and tumor promotion, such as nuclear factor kappa B (NF-κB), nuclear factor erythroid 2-related factor 2 (NRF2), and metal-regulatory transcription factor (MTF1), which are known to induce the expression of metallothionein in response to heavy metal (cadmium) toxicity (Liu et al. 1995).

#### Cadmium and oxidative stress

Cadmium is not a redox-active metal, and therefore cannot directly participate in ROS formation; however, it can indirectly negatively affect the activity of various antioxidant enzymes and cellular proteins, which can in turn increase ROS levels.

Protein and non-protein thiols occur in biological systems at a ratio of approximately 3:1, and both have the capacity to chelate cadmium ions. Cellular thiols play a key role in redox-regulated signal transduction, and following cadmium intoxication, thiols interact with Cd^2+^ ions to form covalent protein-SH-cadmium bonds (Valko et al. 2005). Cadmium can covalently bind not only to cysteine residues of proteins but also to other amino acids, which may result in the breakdown of peptide bonds. This results in suppressed activity of antioxidant enzymes such as SOD, catalase, and glutathione peroxidase and increased oxidative stress, which in turn affects cellular redox homeostasis. Thus, cadmium intoxication is accompanied by increased Cd-induced oxidative stress and potential damage to all important biomolecules, including DNA, proteins, and phospholipid membranes.

The enzyme inhibitory activity of cadmium has been reported for antioxidant enzymes (e.g., superoxide dismutase (SOD) and glutathione peroxidase (GPx)) and metabolic and energy regulatory enzymes (e.g., lactate dehydrogenase and ATPase), resulting in a ROS-mediated increase in oxidative stress and enhanced peroxidation of lipids (Cannino et al. 2009).

Interestingly, cadmium seems to be indirectly involved in the Fenton reaction. Although cadmium does not directly catalyze the decomposition of hydrogen peroxide in the Fenton reaction, it has the capacity to displace endogenous metals from metal-binding sites of storage or transport proteins to increase the pool of redox-active metals (copper or iron) whose catalytic activity in the Fenton reaction increases ROS levels and consequently oxidative stress. In addition, the cellular redox state can be disturbed by the interaction of cadmium with extracellular and intracellular antioxidants such as glutathione (GSH), which is one of the most efficient intracellular non-enzymatic antioxidants (Cuypers et al. 2010a, b).

In addition to GSH, another detoxification mechanism against Cd-induced toxicity and oxidative stress is maintained by metallothioneins (see also above). Metallothioneins can effectively scavenge ROS and thereby protect against Cd-induced oxidative stress and Cd-mediated carcinogenesis (Yang and Shu 2015).

#### Cadmium and mitochondrial apoptosis

Mitochondria play a key role in metabolic energy production in eukaryotic cells through oxidative phosphorylation (OXPHOS). Dysfunctional mitochondria are related to many chronic diseases and aging. Mammalian mitochondrial DNA is more vulnerable to oxidative damage and mutations than nuclear DNA (Venkatesh et al. 2009).

Mitochondrial thiols maintain redox homeostasis and involve glutathione (GSH), thioredoxin-2 (TRX2), and enzymes such as glutathione reductase, which support glutathione peroxidase (GPx), among other systems. All these mitochondrial thiols are critical targets for cadmium toxicity.

Cadmium can inhibit the respiratory chain, which consists of five different enzyme complexes that, in turn, may alter the activity of mitochondrial proteins (transporters across inner and outer membranes), resulting in suppressed respiration, altered mitochondrial membrane potential (ΔΨm), and mitochondrial swelling due to the shift in ion homeostasis of the matrix (Cannino et al. 2009). Mitochondrial complexes I and II pass electrons to complex III and finally to oxygen via complex IV (Diebold et al. 2019). The toxic effect of cadmium is caused by blocking the electron-transfer chain, mainly through the impaired activity of complex III.

Mitochondria are known to play a key role in activating apoptosis in mammalian cells (Wang and Youle 2009). Two distinct apoptotic pathways involve the (i) extrinsic pathway (triggered in response to external stimuli affecting the interaction of ligands with cell surface death receptors, which in turn triggers a caspase cascade) and the (ii) intrinsic pathway (mitochondria-mediated pathway, triggered in response to internal stimuli such as DNA damage). The intrinsic pathway is activated in response to toxic stimuli involving cadmium-mediated ROS formation. The intrinsic pathway is mediated by the Bcl-2 family of proteins. Cadmium stress increases the expression of the proapoptotic protein Bax and downregulates the antiapoptotic protein Bcl-2. Thus, the Cd-mediated decrease in the Bcl-2/Bax ratio in favor of the denominator Bax results in the release of proapoptotic factors such as cytochrome c from the intermembrane space to the cytosol. This activates a caspase cascade, especially caspase-9, which in turn activates caspase-3 (Wang et al. 2019).

#### Cadmium and autophagy

Autophagy is a natural degradation process that removes dysfunctional components, clears damaged organelles, and maintains homeostasis (Shao et al. 2024). By adapting to external stimuli such as toxic metals, autophagy can either be activated or blocked. If the process of adaptation fails, the organism is exposed to inflammation, dysregulated proliferation, and apoptosis, which may lead to cellular injury, cancer, and organ failure.

Moderate levels of ROS induced by cadmium may trigger autophagy, which is considered a self-protection mechanism (Gibson 2013). Cadmium interferes with ROS-triggered autophagy and apoptosis in the liver by enhancing ROS formation and activating the AMPK/PPAR-γ/NF-κB axis (Wang et al. 2022). Similar conclusions have been reached in the retinal pigment epithelium, where Cd induces oxidative stress-activated endoplasmic reticulum (ER) stress, autophagy, and apoptosis (Zhang et al. 2019).

BECLIN-1 is a protein involved in the regulation of autophagy. The BECLIN-1/Bcl-2 complex is an important factor connecting autophagy with apoptosis (Xu and Qin 2019). Transcriptional upregulation of the protein BECLIN-1 is usually associated with an enhanced response to external stimuli (Menon and Dhamija 2018). Cadmium-exposed rat testes exhibit increased expression of cellular BECLIN-1, downregulated expression of the antiapoptotic protein Bcl-2, upregulated expression of the proapoptotic protein Bax, and activated caspase-3 (Arab et al. 2022). Linagliptin, a selective dipeptidyl peptidase-4 (DPP-4) inhibitor that was originally discovered as an antidiabetic drug for treating type 2 diabetes mellitus, was able to restore critical proteins and the balance between autophagy and apoptosis.

The regulatory mechanisms of nuclear factor erythroid 2-related factor 2 (Nrf2) following cadmium exposure in the kidney have been studied (Dong et al. 2021). Cadmium caused injury to cells from rat kidneys (NRK-52E) and primary rat proximal tubular cells, blocked autophagic flux, and promoted the accumulation of protein p62, a stress-inducible clearing protein involved in maintaining the equilibrium between cell death and survival. In addition, cadmium activated p62-dependent nuclear translocation of Nrf2. These results confirmed that cadmium-mediated nuclear translocation of Nrf2 depends on the accumulation of p62, which is directly related to autophagic flux inhibition. The increased nuclear translocation of Nrf2 inactivates the AKT/mTOR cell cycle control pathway, ultimately resulting in cell damage.

#### Cadmium and Na^+^, K^+^-ATPase

Ion-ATPases account for the majority of energy consumption linked to neural signaling (Meyer et al. 2022). Ion-ATPases are strongly linked to glycolytic enzymes in several cell types, and increased ion-ATPase activity can activate glycolysis in multiple ways.

Various animal models have revealed that Cd^2+^ interferes with the renal proximal tubule Na^+^/K^+^-ATPase (van Kerkhove et al. 2010). The effect of Cd^2+^ on Na^+^, K^+^-ATPase is rather indirect than direct. The indirect effect of Cd^2+^ involves extensive ROS formation, which may destabilize the alpha-subunit of Na^+^,K^+^-ATPase. In the case of low/mild Cd intoxication, cells are able to restore the activity of the sodium–potassium pump after the initial decline in ATPase activity; however, if the dose of Cd exposure is very high or if the duration of exposure is too long, the toxic effect dominates. In addition to kidney tubules, the suppressed activity of the sodium‒potassium pump has been observed in hepatic microsomes and microsomes of the rat brain.

### Health effects of cadmium toxicity

**Fig. 2:**
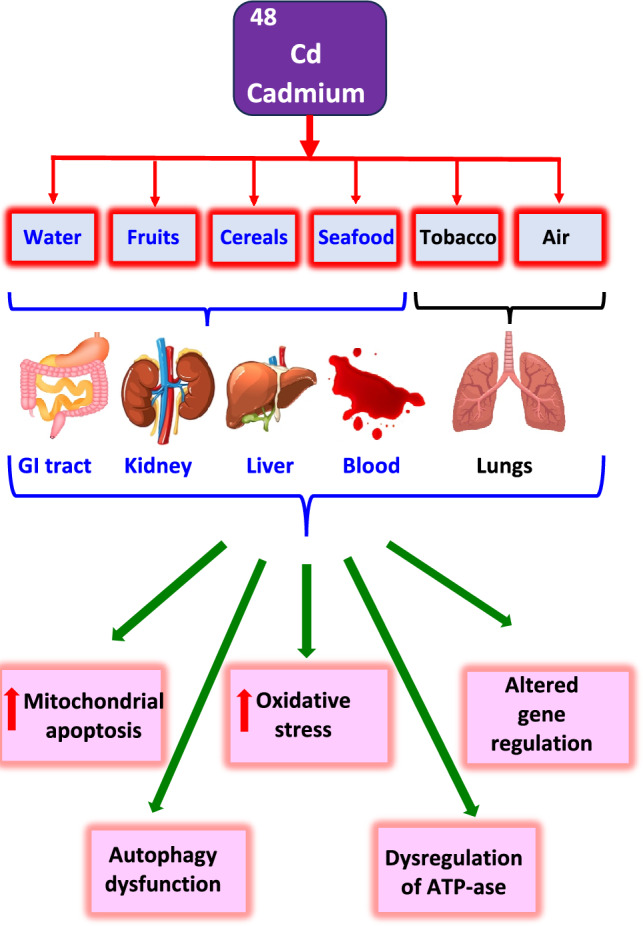
Sources and effects of cadmium toxicity

The organs most affected by cadmium intoxication are consistent with the routes of exposure (Charkiewicz et al. 2023; ATSDR 2010). They involve the respiratory system and gastrointestinal tract. Occupational exposure to cadmium may result in adverse health effects, including various chronic diseases (Fig. 2). Acute inhalation of cadmium is manifested by symptoms resembling flu (fever, body pain), and chronic exposure is predominantly caused by lung and kidney diseases.

#### Cadmium and the respiratory system

*Clinical manifestations:* Accumulated evidence has revealed that the inhalation of cadmium can cause chronic obstructive pulmonary disease (COPD), a chronic inflammatory condition that causes airflow restriction and breathing problems, sometimes termed chronic bronchitis or emphysema (Lampe et al. 2008). Air pollution by cadmium and smoking are typical causes of COPD. Several animal studies have well-documented cadmium-induced lung toxicity. Cadmium intoxication, which is associated with pulmonary inflammation and inhalation of cadmium aerosol (1.6 mg/m^3^ a day) over 6 weeks, results in pulmonary damage in rats (Hart 1986). This and other animal pulmonary toxicity studies proposed that reduced lung function in smokers may also be related to cadmium contained in cigarette smoke (~ 2 μg of cadmium/cigarette), among other factors (Kirschvink et al. 2006).

A mouse model study confirmed that increased pathological scores and alveolar destruction indicators, including airway wall thickness, are consistent with long-term Cd exposure (Wang et al. 2023).

Cross-sectional studies in male adults revealed that a high cadmium content in the blood is associated with the incidence of COPD (Oh et al. 2014). Pathologically altered pulmonary function in COPD patients exposed to cadmium has been attributed to, to some extent, a reduction in telomerase activity. As expected, COPD patients of greater age and smoking habits are more prone to suffer from Cd-associated declines in pulmonary function (Lv et al. 2023). Interestingly, one study reported no correlation between cadmium exposure and altered pulmonary function in children (Pan et al. 2020).

If we accept that cadmium causes cancer, then lung cancer is the most likely diagnosis. Given that occupational workers are often exposed to several heavy metals with carcinogenic properties (e.g., cadmium, arsenic, and lead), it is difficult to evaluate the effect of cadmium itself. In addition, other risk factors, such as smoking, genetic predisposition to cancer, eating habits, age, physical activity, and other factors, may be important. The level of cadmium in the blood of smokers may correlate with the early stages of lung cancer development. In addition to lung cancer, some studies have reported a potential link between cadmium exposure and kidney and prostate cancers (Rapisarda et al. 2018).

*The molecular mechanism of cadmium-induced pulmonary damage:* This type of damage is caused by Cd exposure and involves the upregulation of inflammatory cytokines, including tumor necrosis factor-α (TNF-α), interleukins (IL-1β, IL-6, IL-8, and IL-10), and transforming growth factor-β (TGF-β) (Wang et al. 2021). The transmembrane protein pulmonary E-cadherin, which is involved in cellular adhesion, was downregulated in cadmium-exposed mice; conversely, several mesenchymal markers, including N-cadherin (implicated in cancer metastasis), vimentin (class III intermediate filaments), and α-smooth muscle actin (α-SMA), were markedly upregulated. Long-term cadmium exposure in mice results in the deposition of extracellular matrix (ECM)-containing collagen around small airways. These results confirmed that the decline in COPD-associated lung function is caused by long-term cadmium exposure in mice.

Lung macrophages play important roles in the inflammatory response to injury and the repair of injury. Cadmium-induced injury (100 nanograms/kg) in mice results in the polarization of macrophages (a process of the production of distinct functional phenotypes) to a proinflammatory M1 phenotype (Larson-Casey et al. 2020). In addition, cadmium-induced toxicity enhanced the glycolytic function of macrophages, and the extracellular acidification rate manifested as the cellular release of acidic substances into the extracellular space.

#### Cadmium and renal damage

*Clinical manifestations of cadmium-induced renal toxicity:* The toxic effects of cadmium on kidneys are dose-dependent. In occupationally exposed male workers, the cadmium concentration in urine thresholds for significantly altered renal markers ranged from 2.4 to 11.5 μg cadmium/g creatinine. A threshold of 10 μg cadmium/g creatinine (corresponding to 200 μg cadmium/g renal cortex, which is the critical concentration of cadmium in the kidney) is responsible for the incidence of low-molecular-mass proteinuria and subsequent loss of renal filtration reserve capacity (Roels and Hoet 1999).

Animal models of cadmium-induced renal toxicity have established that the primary target of cadmium is the proximal tubules of nephrons, where it causes damage to epithelial cells, ultimately leading to polyuria and proteinuria (Prozialeck and Edwards 2012). Cadmium renal toxicity is also manifested by increased urinary excretion of glucose, amino acids, charged ions (Na^+^, K^+^, and Ca^2+^), malnutrition, obesity, and diabetes.

Low-level cadmium-induced nephrotoxicity (~ 2 μg/g creatinine) clinically manifests as low-molecular-weight proteinuria, which is characterized by excessive urinary loss of β2-microglobulin, α1-microglobulin, or other low-molecular-weight plasma proteins (Jarup 2002; Jarup et al. 2000). At relatively high levels of cadmium exposure (~ 4 μg/g creatinine), the lysosomal enzyme N-acetyl-B-glucosaminidase (NAG) is increasingly excreted in the urine as an indicator of renal tubular dysfunction.

Glomeruli are blood capillaries located at the beginning of a nephron in the kidney, and their damage (glomerular disease) may allow blood to enter the urine. Cadmium may cause damage to glomeruli via a suppressed glomerular filtration rate, resulting in a disturbed balance of certain substances in the bloodstream, such as increased levels of albumin in the urine.

Cadmium-induced nephropathy is one of the most important causes of mortality in workers exposed to cadmium (ATSDR 2010). The advanced stages of cadmium-induced nephropathy are clinically manifested by glycosuria, characterized by the presence of reducing sugars (e.g., glucose, galactose) in the urine, altered calcium homeostasis, and the wasting of calcium and phosphate. The secondary effects of nephropathy are osteoporosis (lowered bone mass but a normal ratio of bone mineral to bone matrix) and osteomalacia (lowered ratio of bone mineral to bone matrix).

*Molecular mechanisms of cadmium-induced renal damage:* The unified view of scientists on the mechanism of cadmium-induced kidney damage points to ROS-induced oxidative stress in the proximal tubules of nephrons (Yan and Allen 2021). The major sources of cadmium-induced ROS in the kidney are mitochondria and NADPH oxidase.

The mitochondrial electron transport chain components complexes I, II, and III have all been established as major sites of ROS production. Leaked electrons from the electron transport chain can partially reduce oxygen to superoxide radicals, which can be converted to hydrogen peroxide, finally leading to the redox-metal-catalyzed formation of hydroxyl radicals (the Fenton reaction), causing damage to DNA, protein modification, and lipid peroxidation. The most damaged complexes are complexes II and III.

**Fig. 3:**
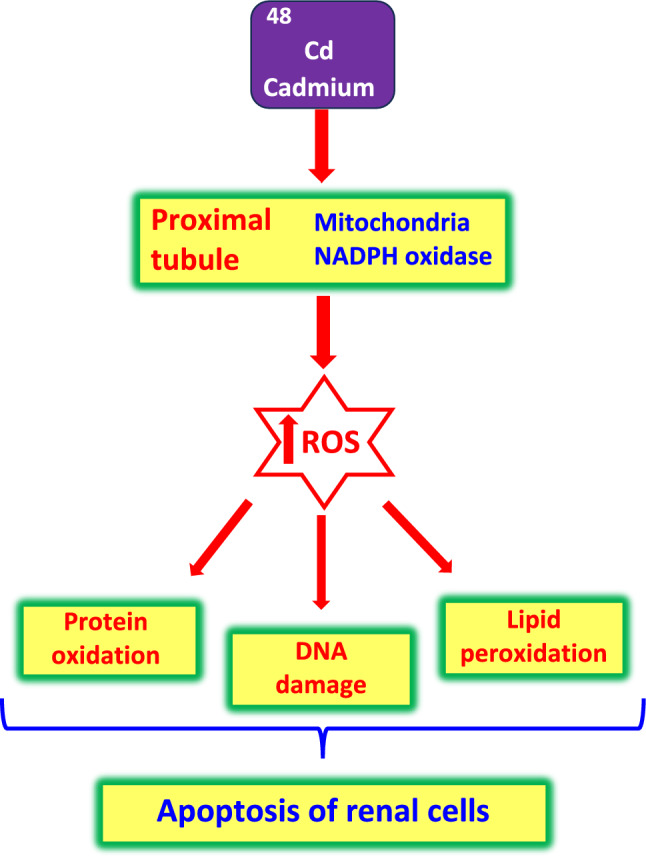
Cadmium-mediated oxidative stress and renal injury

After entry and accumulation in mitochondria, cadmium can bind to protein thiol groups that impair protein function (Cannino et al. 2009). Following cadmium exposure, kidney mitochondria display swelling and deformation, increased expression of SOD1, and suppressed expression of SOD2 and catalase (Liu et al. 2019). Cadmium exposure is responsible for the downregulation of the antiapoptotic protein Bcl-2 and the increase of the oxidized (GSSG) to reduced (GSH) glutathione ratio (Nair et al. 2015). Thus, renal injury is largely caused by increased levels of cadmium-induced oxidative stress, damage to all important biomolecules, and/or cell death through both the apoptosis and even necrosis of all types of kidney cells (Fig. 3).

#### Cadmium and cardiovascular diseases

*Clinical manifestations of cadmium-induced cardiovascular diseases*: Among other adverse effects, cadmium intoxication has been associated with an increased risk of cardiovascular diseases. Urinary cadmium has been correlated with hypertension, indicating that the development of cardiovascular disease is tightly linked with the occurrence of cadmium-induced renal diseases. However, various epidemiological studies have reported that the effect of cadmium on cardiovascular diseases is not straightforward (Charkiewicz et al. 2023).

Chronic cadmium exposure reportedly increases systolic blood pressure (Noor et al. 2018). Hypertension has been attributed to decreased tissue and blood levels of atrial natriuretic peptide, a hormone secreted from the right atrium involved in the regulation of saltwater balance. In addition, the toxic effect of cadmium on the cardiovascular system is accompanied by increased blood levels of the steroid hormone aldosterone and retention of sodium and water (ATSDR 2010).

Positive associations between cadmium exposure and overall cardiovascular diseases, risk of heart failure, stroke, and coronary heart diseases have been reported. The results suggest that the level of cadmium intoxication quantified in blood or urine is associated with an increased risk of cardiovascular diseases (Verzelloni et al. 2024).

*Molecular mechanisms of cadmium-induced cardiovascular diseases:* Animal experiments reported that cadmium is an inhibitor of mitochondrial respiration, decreases the activity of important antioxidant enzymes such as superoxide dismutase (SOD) and glutathione peroxidase (GPx), and increases thiobarbituric acid reactive substances (TBARS), which are formed as a byproduct of lipid peroxidation (Okoye et al. 2022). Thus, cadmium-induced toxicity impacts cardiac repair mechanisms, the activation of apoptosis, metabolic processes, and increased ROS formation.

Cadmium stimulates the production of macrophages and monocytes, which in turn affects the development and promotion of atherosclerosis. The development of atherosclerotic lesions is associated with the death or apoptosis of foam cells, resulting in the formation of necrotic foam (Rasin and Sreekanth 2023).

Cadmium affects the flow of calcium and electrolyte balance (see above), thus causing toxicity to smooth muscle cells, which results in the accumulation of lipids in blood vessel walls, enhancing the effect of atherosclerosis (Lin et al. 2021). Cytokines and inflammation affect atherosclerosis. Following cadmium exposure, the levels of proinflammatory cytokines such as the interleukins IL-8, IL-1, and tumor necrosis factor (TNF-α) increase, which directly affects atherosclerosis.

Long-term cadmium exposure increases total cholesterol levels and decreases cholinergic relaxation, which negatively affects the synthesis of nitric oxide, manifested by altered vasorelaxation and consequently the regulation of blood pressure (Lin et al. 2021).

#### Cadmium and cancer

*Clinical manifestations of cadmium-induced cancer:* Cadmium and related cadmium-based inorganic compounds were classified as carcinogenic to humans by the International Agency for Research on Cancer (IARC) (Hartwig 2013; IARC 2012) and nearly 2 decades ago as human carcinogens by the German MAK Commission (Grein 2006).

The involvement of cadmium among carcinogens is due primarily to sufficient evidence for an increased relative risk of lung cancer in workers occupationally exposed to cadmium. Extended follow-up studies have been carried out on initial cohorts in the U.S., Sweden, and the UK, with most of these studies indicating increased risks of lung cancer (Sorahan 2009).

Elevated lung cancer risk was observed in the group of subjects who lived in Belgium near three smelters. During the investigated period from 1985 to 2004, the subjects in the high-exposure group presented an elevated risk of lung cancer, which was supported by increased urinary cadmium excretion (Nawrot et al. 2006).

Compared with other cancer sites, the kidney in particular may be at greater risk because of its known nephrotoxicity and large and ongoing body burden. In contrast, neither a Swedish cohort study nor a British cohort study reported increased risks for renal cancer as a result of cadmium exposure (Sorahan and Esmen 2004; Jarup et al. 1988). However, case–control studies that estimated the relative risk of kidney cancer due to occupational cadmium exposure conducted in the U.S., Finland, Germany, and Canada reported that increased incidences of renal cancer were associated with workplace cadmium exposure. These studies used job-exposure matrices (JEMs) to estimate cadmium exposure.

*Molecular mechanism of cadmium carcinogenesis:* The carcinogenic effect of cadmium is likely caused by several different mechanisms (Hengstler et al. 2003). Interactions of proteins and other target systems with cadmium seem to be more important for the induction of carcinogenicity than direct DNA damage. These target systems involve DNA repair pathways, tumor suppressor and signal transduction proteins, and antioxidant defense systems. While cadmium-induced activation of each of these targets may trigger a carcinogenic effect, their collective activation may exhibit a synergistic effect (Hartwig 2013; IARC 2012).

Since cadmium-based compounds interact preferentially with proteins rather than causing DNA damage in cell extracts or isolated DNA (Hu and Mao 2002), indirect processes likely underlie cadmium genotoxicity. In agreement with these findings, cadmium-induced genotoxicity may be related primarily to increased oxidative stress and interactions with DNA damage response mechanisms. Certain cadmium compounds are capable of causing oxidative DNA base alterations and DNA strand breaks; however, these effects are often only observed at substantially increased cadmium concentrations (Schwerdtle et al. 2010).

Although the effects of cadmium compounds on point mutations are typically low and/or limited to relatively high cadmium concentrations in mutagenicity assays, cadmium-based substances cause clastogenic effects, including micronuclei, breaks in chromosomes, and changes in chromosome number (Beyersmann and Hartwig 2008; Filipic et al. 2006). Clastogenic effects have been confirmed by enhanced cadmium-mediated multilocus deletions (Filipic and Hei 2004).

Unlike redox-active metals such as copper or iron, cadmium cannot take part in redox reactions such as the Fenton reaction or direct ROS formation. However, contrary to this assumption, both in vivo and in vitro experiments reported elevated ROS levels as a result of cadmium exposure (Liu et al. 2009).

Cadmium-induced oxidative DNA damage has been attributed to the inhibited activity of antioxidant enzymes (SOD, catalase, and glutathione peroxidase) and the suppression of overall antioxidant defense mechanisms, resulting in increased oxidative stress-induced DNA damage (Hartwig 2013; Liu et al. 2009). An alternative explanation for cadmium-induced oxidative damage is that structurally similar cadmium may displace redox-active metal ions (e.g., copper or iron) from their natural binding sites, which may in turn catalyze the decomposition of hydrogen peroxide in the Fenton reaction, producing damaging hydroxyl radicals (Valko et al. 2006). The suppression of cadmium-induced DNA strand breaks and chromosomal abnormalities in mammalian cells by the application of low-molecular-weight antioxidants and antioxidant enzymes confirmed the involvement of ROS in this process (Valko et al. 2006; Ochi and Ohsawa 1985).

Cadmium-induced ROS may interact with several redox-controlled signaling pathways (Hartwig 2013). This comprises signaling through protein kinases that are both Ca^2+^-independent and cAMP-independent, as well as signaling through cyclic adenosine monophosphate (cAMP) and nitric oxide (NO^**·**^). Moreover, cadmium activates transcription factors such as nuclear factor kappa B (NF-κB) and nuclear factor erythroid 2-related factor 2 (Nrf2). Cadmium induces the expression of protooncogenes, such as c-fos, c-jun, and c-myc, which are activated in response to mitotic stimuli and are often overexpressed in malignancies. Activator protein 1 (AP-1) is a transcription factor composed of the genes c-fos and c-jun that activates several genes related to cell division and growth. Cadmium suppresses negative regulators of cell proliferation, for example, by inactivating p53, in addition to directly activating mitogenic signals.

Prolonged treatment of prostate epithelial cells with cadmium reportedly results in malignant transformation (Qu et al. 2007). The transformed cells exhibited acquired resistance to apoptosis, which was attributed to an increase in antiapoptotic Bcl-2 protein activity, which disrupted the JNK signal transduction pathway.

An interesting link between metallothionein and cadmium exposure has been reported (Hartwig 2013). Metallothioneins are relatively small cysteine-rich proteins that bind heavy metals such as cadmium, zinc, and copper. Numerous genes, including those encoding not only metallothionein but also GSH synthesis, superoxide dismutase, and catalase, are induced by cadmium. As a result, the presence of cadmium extends cell survival, which may be beneficial for protection against acute cadmium toxicity, as demonstrated by comparative experiments using metallothionein-transgenic and metallothionein-null mice (Klaassen et al. 2009). Nevertheless, adaptation to cadmium might have two pitfalls: on the one hand, elevated levels of metallothionein might result in cadmium buildup and its extended half-life; however, on the other hand, decreased DNA repair and apoptosis may occur (Singh et al. 2009).

Future studies must focus on the relevance of the corresponding cadmium-mediated pathways in exposed humans and experimental animals, given the new reports on the carcinogenicity of cadmium in several target organs under low-exposure settings, which are typical for a work environment with the presence of heavy metals.

## Arsenic

Arsenic (As) is the 33rd element in the periodic table and most commonly occurs at oxidation numbers of + 5, + 3, 0, and − 3. The electronic configuration of arsenic is [Ar]3d^10^4s^2^4p^3^. Arsenic is formally considered a metalloid; it has properties of both metals and nonmetals and can exist in three allotropic α (yellow), β (black), γ (gray), and two organic and inorganic forms (Housecroft 2018). From a toxicological point of view, arsenic is classified as a heavy metal and is distributed in nature in soils and water, representing a toxicity risk for both humans and nature.

### Arsenic in the environment

In nature, arsenic is found in sediments, ores, or igneous rocks and often has an affinity for elements such as sulfur or oxygen. An important source of arsenic is red ore arsenopyrite, a mixture of arsenic, iron, and sulfur. The most abundant form of arsenic in the air is inorganic arsenic trioxide, As_2_O_3_. Water and soil contain predominantly inorganic arsenates iAs^V^, [AsO_4_]^3−^ and/or arsenites iAs^III^ [AsO_3_]^3−^.

Industrial exposure to arsenic includes the processing of glass, textiles, ammunition, paper, wood preservatives, and other industries. In addition, in the cosmetic industry, color pigments used in the production of eye shadows frequently contain toxic metals, including arsenic. Arsenic present in cosmetic products can enter the circulatory system through percutaneous penetration; therefore, the limit for cosmetic products is less than 5 ppm of heavy metal impurities. The largest source of arsenic in organic form is seafood, mushrooms, rice, and poultry (Jones 2007).

The areas contaminated with arsenic include some regions in Bangladesh and West Bengal (India), where more than 40 million people use water contaminated with arsenic (Chowdhury et al. 2000). According to the WHO, the limit for As in water is 10 µg/l.

For therapeutic reasons, Chinese herbal products are enriched with arsenic, which in turn has caused the intoxication of consumers all over the world (Martena and Wielen 2010). Quantitative mass spectrometry revealed that samples sold on the Dutch market exceeded the safety levels of heavy metals (not only As, but also Hg and Pb) by 20%. Percutaneous absorption of high concentrations of arsenic may present a carcinogenic risk; therefore, the content of heavy metals should be regularly controlled in cosmetic products.

### The fate of arsenic in the human body

The main routes of arsenic intoxication are ingestion, inhalation, and partly dermal absorption (ATSDR 2023). Following absorption, arsenic is absorbed into the bloodstream and transported by red and white blood cells throughout the body, mainly to the liver and, to a much lesser extent, to the kidneys, muscles, heart, lungs, pancreas, and brain. Several weeks after arsenic exposure, residual amounts are found mainly in hair and nails (Yip and Dart 2001).

**Fig. 4:**
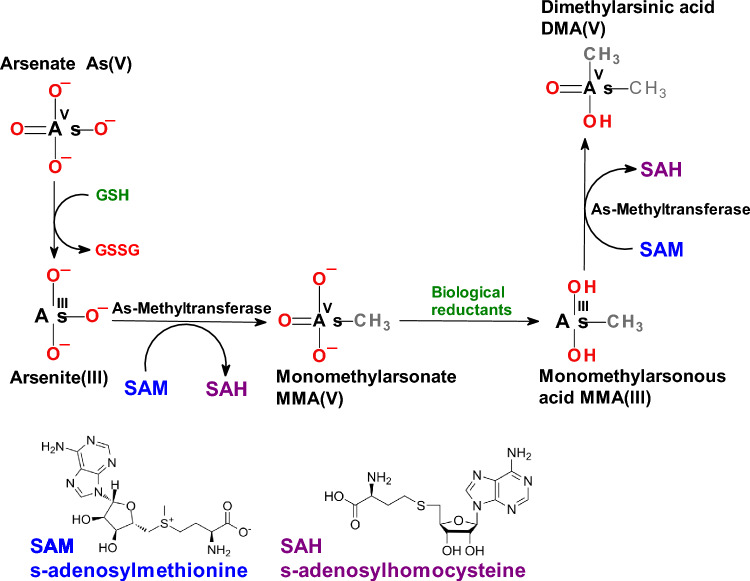
A simplified scheme of arsenic metabolism. Arsenate, As(V), is reduced by glutathione (GSH) to arsenite, As(III). As(III) is oxidatively methylated via the enzyme arsenite methyltransferase (As-methyltransferase) to form monomethylarsonate, MMA(V). This reaction uses s-adenosylmethionine (SAM) as the methyl donor, which is converted during the course of the reaction to s-adenosylhomocysteine (SAH). MMA(V) is then reduced by biological reductants to monomethylarsonous acid, MMM(III). MMA(III) is again oxidatively methylated by arsenite methyltransferase (As-methyltransferase) to form dimethyl-arsinic acid, DMA(V). For more details, see the text

The liver is the main organ where arsenic undergoes biomethylation. Pentavalent arsenates [AsO_4_]^3−^ are first reduced by glutathione (GSH) to trivalent arsenites [AsO_3_]^3−^ (Miller et al. 2002), followed by enzymatic oxidative methylation catalyzed by the enzyme methyltransferase to form monomethylarsonates, MMA(V). The source of methyl groups in the reaction is the s-adenosylmethionine (SAM), which is converted during the course of the reaction to s-adenosylhomocysteine (SAH). In the next step, the monomethylarsonate, MMA(V) is reduced to monomethylarsonous acid, MMA(III), by the biological reductants, before the second enzymatic oxidative methylation reaction, catalyzed by the enzyme methyltransferase, forming dimethylarsinic acid, DMA(V). Methylation is again achieved by the conversion of s-adenosylmethionine (SAM) to s-adenosylhomocysteine (SAH) (Fig. 4) (Hall and Gamble 2012; Aposhian et al. 2000; Chen and Rosen 2020). The resulting metabolites are more readily excreted.

Thus, the detoxification pathway of inorganic arsenic produces monomethylarsonous acid, MMA(III), and also dimethylarsinous acid, DMA(III), which have been found to interfere with cellular targets and important biomolecules such as DNA and proteins and thus act as potential toxic agents (Kitchin 2001). In the case of overloaded methylation capacity of the liver, excess arsenic is stored in soft tissues. In addition to methylation, alternative detoxification mechanisms, such as enhanced antioxidant defense or downregulation of apoptosis, should be considered (Yoshida et al. 2004).

Detoxification of arsenic from the body occurs mainly through the urine, a much smaller portion through feces, and deposition into hair and nails (Buchet et al. 1981). Approximately half of the excreted arsenic in urine is dimethylated, and a quarter is monomethylated. The rest is inorganic arsenic.

### Mechanisms of arsenic toxicity

#### Impact of arsenic on energy metabolism

Arsenic has the capacity to inhibit many enzymes important for cellular processes, including energy metabolism and ATP production (Muzaffar et al. 2023; Shen et al. 2013). Arsenic and phosphorus are in group 15 of the periodic table and have similar chemical properties. Owing to similar structural characteristics, arsenate [AsO_4_]^3−^ can replace inorganic phosphate to form unstable and readily hydrolyzable 1-arseno-3-phosphoglycerate, which ultimately leads to arsenate-induced uncoupling of the glycolytic production of energy (Garrett and Grisham 2012).

The pyruvate dehydrogenase complex (PDC) consists of three enzymes that convert pyruvate into acetyl-coenzyme A, providing ATP for cell metabolism. Arsenite can interact with the pyruvate complex PDC, thus uncoupling ATP synthesis. The α-ketoglutarate dehydrogenase complex (KGDHC) is a mitochondrial enzyme complex that catalyzes one of the key steps in the tricarboxylic acid (TCA) cycle. In this case, following its interaction with the KGDH complex, arsenite can also uncouple mitochondrial respiration (Bergquist et al. 2009).

It has been reported that arsenic interacts with specific sites of proteins in the glycolytic pathway. Among many such proteins, hexokinase isozymes (hexokinase-1 (HK-1) and hexokinase-2 (HK-2)) are sugar kinases that catalyze the first step in glucose metabolism, the phosphorylation of glucose (Zhang et al. 2015). In connection with hexokinases, an interesting opposite mechanism was observed. While in cancers, the glycolytic pathway is significantly increased, the interaction of arsenic with hexokinases has a significant inhibitory effect, indicating that, in addition to its toxic effect, arsenic may paradoxically exert prospective anticancer effects. Such an unusual property of arsenic is used in arsenic-based compounds that exhibit promising effects in the treatment of cancers characterized by high remission rates, such as acute leukemia (Falchi et al. 2016).

#### Arsenic-mediated ROS formation

The inhibitory effect of arsenic on energy metabolism is complemented by arsenic-mediated oxidative damage. Arsenic can induce ROS formation in several cells, including vascular endothelial cells, leukocytes, vascular smooth muscle cells, and various other cells (Medda et al. 2021). The detrimental effects of As can be reversed by increased levels of antioxidant enzymes and low-molecular-weight ROS scavengers (Kessel et al. 2002). In addition to oxygen-derived free radicals (ROS), arsenic exposure has been reported to generate nitrogen-derived free radicals (RNS), including nitric oxide (NO^**·**^); however, the results are not very convincing (Shi et al. 2004).

Trivalent arsenicals (inorganic and organic) are highly reactive species that interact with protein thiols (–SH) and inhibit their activity. Other arsenic-mediated toxic mechanisms include inflammation and epigenetic alterations (Hu et al. 2020). Arsenic has been reported to decrease the levels of antioxidant enzymes, including superoxide dismutase (SOD) and catalase (CAT), activate NADPH oxidase, and reduce the mitochondrial membrane potential via the release of cytochrome c (Ali et al. 2016; Medda et al. 2020). The ROS derived from reduced oxygen as a result of the toxic action of arsenic include superoxide radical anion (O_2_^**·**−^), singlet oxygen (^1^O_2_), hydroxyl radicals (^**·**^OH), peroxyl radicals (ROO^**·**^), perhydroxyl radicals (HO_2_^**·**^), and other redox species.

The possible mechanisms of arsenic-induced ROS formation include the following:

#### Mitochondria

Arsenic in pentavalent and trivalent forms inhibits mitochondrial respiration and uncouples mitochondrial oxidative phosphorylation in a comparable manner (Corsini et al. 1999). Arsenic-induced uncoupling of mitochondrial oxidative phosphorylation may divert electrons toward excessive ROS (superoxide radical) formation (Cadenas et al. 1977). Studies using isolated mitochondria revealed that the major source of ROS derived from the mitochondrial chain is ubiquinone. Indeed, As-induced oxidative activity was abrogated when the complex I inhibitor rotenone was used.

Pyruvate dehydrogenase (PDH) is a mitochondrial multienzyme complex containing sulfhydryl groups that catalyzes the irreversible conversion of pyruvate to acetyl coenzyme A. Pyruvate dehydrogenase is one of the primary targets of heavy metal toxicity. As_2_O_3_ inhibits PDH through direct interaction with vicinal dithiols in pure enzymes (Samikkannu et al. 2003). However, in human leukemia (HL60) cells, As_2_O_3_ inhibited PDH mainly via the generation of ROS (and not through interactions with vicinal thiols), and the level of inactivation in HL60 cells was approximately 38 × greater than that in pure enzymes. It has been proposed that As_2_O_3_ triggers the production of hydrogen peroxide in mitochondria and that the presence of redox metals (copper, iron) catalyzes the decomposition of hydrogen peroxide via the Fenton reaction, producing hydroxyl radicals and causing oxidative damage to the PDH protein.

#### ROS formation during the transformation of arsenic species

The mechanism of enhanced ROS formation involves the oxidation of arsenite (As^3+^) to arsenate (As^5+^) species according to the following reaction (Valko et al. 2005, 2016):

$${\text{H}}_{3}{\text{AsO}}_{3}+{\text{H}}_{2}\text{O}+{\text{O}}_{2} \to {\text{H}}_{3}{\text{AsO}}_{4}+{\text{H}}_{2}{\text{O}}_{2} ({\Delta }_{r}\text{G}=-172\text{ kJ}/\text{mol})$$

The above reaction has negative Gibbs energy (− 172 kJ/mol), is spontaneous and exergonic, and produces hydrogen peroxide, which can be converted by traces of copper (Cu^+^) or iron (Fe^2+^) to hydroxyl radicals (^**·**^OH) (Fenton reaction), causing damage to biomolecules.

$${\text{Fe}}^{2 + } / {\text{Cu}}^{ + } + {\text{H}}_{{2}} {\text{O}}_{2} \to {\text{Fe}}^{3 + } /{\text{Cu}}^{2 + } + ^{\cdot} {\text{OH}} + {\text{OH}}^{ - } \left( {{\text{Fenton }}\,{\text{reaction}}} \right)$$

*NADPH oxidase* is a membrane-associated enzyme complex that regulates various redox-dependent signaling pathways (Skonieczna et al. 2017; Gupta et al. 2013). The application of a series of techniques, including gene expression profiling, engineered cells, and other techniques, revealed that NADPH oxidase is the main target of As-induced superoxide radical production which can be converted into hydrogen peroxide, one of the most effective cellular oxidants and important signaling molecule (Chou et al. 2004).

*The endoplasmic reticulum* is a critical target for metalloids, including arsenic. Endoplasmic generation of ROS in rat liver cells-16 (RLC-16) was studied following exposure to trivalent dimethylarsinous acid [(CH_3_)_2_AsOH, DMA(III)] (Naranmandura et al. 2012). DMA(III) exposure was shown to generate ROS in the endoplasmic reticulum, probably through the activation of the endoplasmic reticulum as a consequence of As-induced inhibition of the protein folding mechanism.

#### Arsenic-induced activation of signaling pathways

Arsenic-induced oxidative stress triggers upregulation of nuclear factor erythroid 2-related factor 2 (Nrf2), a master redox-sensitive transcriptional regulator of the antioxidant response to oxidative stress. Inorganic arsenic activates Nrf2/heme oxygenase-1 (HO-1). The activation of Nrf2 in response to arsenic exposure in various cell lines, such as hepatocytes, keratinocytes, and osteoblasts, enhances the synthesis of heme oxygenase-1, which in turn results in the synthesis of various bioactive metabolites (Chiu et al. 2016; Choi 2019).

The double-edged sword role of Nrf2 in response to arsenic toxicity substantiated by excessive ROS formation and increased oxidative stress is (i) protection against oxidative stress by increased expression of antioxidant enzymes and, conversely, (ii) sustained activation of Nrf2 associated with apoptotic resistance, metabolic reprogramming and increased survival of oncogenic cells and cancer development (Niture and Jaiswal 2012).

*Mitogen-activated protein kinases (MAPKs)* are serine-threonine protein kinases involved in the regulation of proliferation, survival, apoptosis, and various other cellular processes. The best-known MAPK pathways in mammalian cells are the extracellular signal-regulated kinases 1 and 2 (ERK1/2), the c-Jun N-terminal kinases 1, 2, and 3 (JNK1/2/3), the p38 MAPK α, β, δ, and γ pathways and the extracellular signal-regulated kinase 5 (ERK5) family (Soares-Silva et al. 2016).

Arsenic is known to activate MAPKs in a concentration (up to 500 µM)- and time-dependent manner. Dose-dependent arsenic exposure can activate different mitogen-activated protein kinases (Hou et al. 2014). Chronic arsenic exposure (500 nM, 20 weeks) is relevant to chronic occupational exposure to arsenic and induces continuous activation of p38 and a malignant cellular phenotype in human hepatocytes. It has also been shown that during chronic arsenic exposure, glutathione (GSH) synthesis is, to some extent, controlled by p38. Acute arsenic exposure activated ERK1/2 and JNK and increased the transcription of Nrf2 and the expression of glutamate-cysteine ligase, which is the first rate-limiting enzyme of glutathione synthesis. These results confirmed that the MAPK pathway is sensitive to arsenic intoxication and related glutathione synthesis.

*Nuclear factor-κB (NF-κB)* is mediated by two key signaling pathways, the canonical and non-canonical pathways, which differ in their mechanisms and biological functions (Sun 2017). Ligands of diverse immune receptors stimulate the canonical pathway, and the non-canonical pathway responds to signals from tumor necrosis factor receptor (TNFR) family members.

Human fetal hepatocyte cells (*L*-*02*) were treated with low levels of As^3+^ (0.2 µmol/L) (Yin et al. 2024). Arsenic induced ROS formation and activated the NF-κB signaling pathway via the phosphorylation of p65 at the Ser536 and Ser276 sites, resulting in the upregulation of hexokinase-2 (HK2), a key mediator of aerobic glycolysis and a promoter of cell proliferation in various cancer cell lines. Thus, low levels of arsenic activate the ROS/NF-κB/HK2 axis, which in turn affects glycolysis and cell proliferation, two mutually interconnected phenomena.

An interesting health-beneficial association between the tumor suppressor protein p53 and arsenic trioxide (As_2_O_3_), an established agent used to treat acute promyelocytic leukemia, has been reported (Chen et al. 2021). The *TP53* (tumor protein p53) gene is the most frequently mutated gene (> 50%) responsible for many human cancers. Arsenic trioxide is a cysteine-reactive substance that can reverse structural p53 mutations. Structural analysis of arsenic-bound p53 mutants revealed the coordination of three cysteines to arsenic within the DNA-binding domain, stabilizing the DNA-binding loop-sheet helix motif. Arsenic interactions support the thermostability and transcriptional activity of p53 mutants. Importantly, arsenic trioxide reactivated mutant p53 for tumor suppression. These results may provide avenues for anticancer therapies based on targeting p53 mutations through the use of arsenic trioxide.

### Health effects of arsenic toxicity

Since arsenic interferes with many enzymatic reactions in biological systems, it represents a potential hazard for several organs. There is a strong link between chronic arsenic toxicity and skin, lung, and bladder cancers. Gastrointestinal and hepatic disorders are the result of arsenic ingestion and exposure via other routes. Arsenic affects multiple organ systems and may result in a variety of cardiovascular disorders, neurological effects, respiratory problems, and kidney disease (ATSDR 2023).

Following absorption, the liver primarily methylates inorganic arsenic (iAs, which includes arsenate and arsenite) to produce monomethylated and dimethylated compounds (MMA, DMA), which are then eliminated through the kidney together with unmethylated inorganic arsenic (Vahter 2002). However, there is still disagreement over whether the methylation process of arsenic is a detoxifying or potentiating mechanism (Syblo et al. 2000).

There are significant inter- and intra-population fluctuations in the relative amount of arsenic metabolites in urine, which has been estimated to be approximately 10–30% of inorganic arsenic, 10–20% of MMA, and 60–80% of DMA (Hernandez and Marcos 2008; Steinmaus et al. 2005).

It has been reported that Taiwan, Mexico, and the US populations have higher DMA% and lower MMA% values, which are related to diabetes-related outcomes and higher body mass indices (Gribble et al. 2013). Conversely, in populations from Taiwan, Bangladesh, and Argentina, higher levels of MMA% and lower levels of DMA% have been linked to cancer and cardiovascular outcomes (Chen et al. 2013; Steinmaus et al. 2006; Kuo et al. 2017).

#### Arsenic and cancer

According to several agencies, such as the International Agency for Research on Cancer, the National Toxicology Program, and the US Environmental Protection Agency, arsenic has been classified as a known human carcinogen (IARC 2004). Long-term exposure to arsenic from drinking water can cause lung, urinary, skin, bladder, and other organ cancers in a dose-dependent manner.

*Skin cancer:* The degree of hyperpigmentation and hyperkeratosis of the skin is a typical sign of chronic arsenic exposure and indicates the possible incidence of skin cancer. The most frequently occurring arsenic-induced skin cancers are basal cell carcinoma (BCC), a very early form of squamous skin cancer in situ (Bowen’s disease), and squamous cell carcinoma (SCC) (Yu et al. 2006). Interestingly, arsenic-induced skin cancers frequently develop in sun-protected areas (Yu et al. 2001).

As discussed above, arsenic interacts with protein-SH groups and inhibits the activity of glutathione (GSH), which increases the level of oxidative stress manifested by increased oxidative damage in mouse and human skin. Oxidative damage has been documented by increased levels of 8-hydroxyguanine (8-OH-Gua), one of the major modified DNA bases found in many cancerous tissues (Matsui et al. 1999). The level of arsenic-induced Bowen’s disease was positively correlated with the level of 8-OH-Gua. Low levels of arsenic (less than 5 µM) upregulated the expression of nuclear factor kappa B (NF-κB) and activator protein (AP-1), which promote cell proliferation (Germolec et al. 1996).

In addition, arsenic significantly affects the regulation of p53. Studies of Bowen’s disease lesions revealed that arsenic exposure was linked with G2/M cell cycle arrest and DNA aneuploidy (Yih et al. 2005). These and other cellular disorders indicate that p53 dysfunction is induced by arsenic exposure.

*Lung cancer:* Although the exact mechanism of arsenic-induced lung cancer is unclear, the biotransformation of arsenic plays an important role in this process (Wei et al. 2019). The most critical group of people at risk of arsenic-induced cancer are smokers; however, a considerable number of people at risk of lung cancer are also non-smokers (Steinmaus et al. 2013). The most common type of As-induced lung cancer is squamous cell carcinoma.

Although arsenic is not a direct genotoxic agent, it is an oncogenic promoter that increases cell proliferation and/or inhibits DNA repair in animals (Rossman et al. 2002). Interestingly, it has been reported that arsenic increases the carcinogenic effect of other potential cancer-causing agents, such as benzo[a]pyrenes (Evans et al. 2004).

Angiogenesis is the process of the formation of new blood vessels. Short-term arsenic exposure induced angiogenesis in human lung epithelial cells (BEAS-2B) and adenocarcinoma cells (A549). The mechanism of arsenic-induced angiogenesis involves the excessive formation of ROS, which in turn activates one of the frequently deregulated pathways in human cancers, involving the Akt signaling pathway, the extracellular signal-regulated kinase 1/2 (ERK1/2) signaling pathway, and the increased expression of hypoxia-inducible factor 1 (HIF-1) and vascular endothelial growth factor (VEGF) (Liu et al. 2011; Gao et al. 2023). Antioxidant administration suppressed ROS production and decreased angiogenesis via the deregulation of the Akt and ERK activities associated with HIF-1 expression. The mechanism of arsenic-regulated angiogenesis plays an important role in the development of interventions to prevent As-mediated carcinogenesis and angiogenesis.

Histones are small alkaline proteins that are important for DNA packaging, sustained chromosomal stability, the promotion of cell-mediated apoptosis, and many other functions (Henikoff and Smith 2015). The association between histone modifications and cancer is well-established (Audia and Campbell 2016). Arsenic can modify histones via acetylation, methylation, and phosphorylation, resulting in increased expression of oncogenes and chromosomal structural changes. Histone H3 lysine 9 (H3K9) methylation is a key central epigenetic modification that is increased by arsenic in human non-small cell lung carcinoma A549 cells. This and other studies provide evidence that the mechanism of arsenic (and some other heavy metals) carcinogenicity involves histone modifications that may affect the expression of specific genes important in cancer development (Zhou et al. 2009).

*Bladder cancer:* The human bladder is one of the primary targets of As-induced cancer. Arsenate, monomethylated arsenic, dimethylated arsenic, and other arsenic metabolites detected in urine are associated with the risk of bladder cancer.

Populations living in areas contaminated with arsenic have an increased risk of bladder cancer (Koutros et al. 2018). The incidence of bladder cancer risk in a population exposed to low-to-moderate arsenic levels can be affected by various lifestyle and biological factors, including smoking habits, weight (BMI), alcohol consumption, nutrient intake, and other factors.

The subject of the detailed study was the cytotoxicity of various arsenic compounds in a human bladder carcinoma cell line (EJ-1) (Naranmandura et al. 2011). The highest level of cytotoxicity was observed for dimethylarsinous acid (DMA(III)) and dimethylmonothioarsinic acid (DMMTA(V)), followed by inorganic arsenite (iAs(III)), monomethylarsonic acid (MMA(V)), dimethylarsinic acid (DMA(V)) and dimethyldithioarsinic acid (DMDTA(V)), indicating that the sulfur-containing metabolite DMMTA(V) was one of the most toxic metabolites. Treatment of cells with DMMTA(V) resulted in suppressed expression of the proteins p53 and p21 and increased levels of hydroxyl radicals, which contributed to an increase in the overall level of DNA damage. EJ-1 human bladder cancer cells treated with dimethylarsinous acid (DMA(III)) or dimethylmonothioarsinic acid (DMMTA(V)) presented a 60% decrease in glutathione (GSH), indicating the involvement of oxidative stress in cell death. EJ-1 cells exposed to inorganic arsenite (iAsIII) presented increased expression of glutathione (GSH) and slightly increased levels of ROS; however, these effects were detected only in the case of long-term exposure to arsenite. In summary, dimethylmonothioarsinic acid (DMMTA(V)) has been confirmed as one of the most toxic arsenic metabolites with potential carcinogenic effects on the urinary bladder.

#### Arsenic and neurological disorders

The brain requires a significant amount of energy and is especially susceptible to oxidative damage caused by various toxic substances, including arsenic. Arsenic inhibits the function of antioxidant enzymes, increasing the level of oxidative stress, which is a key mechanism of arsenic-induced neurotoxicity (Thakur et al. 2021). Neurotoxic effects induced by arsenic include a reduction in brain weight, a decrease in the number of neuroglial cells and neurons, and the dysregulation of neurotransmitters (Mochizuki 2019).

Enhanced oxidative stress and mitochondrial dysfunction are major neurotoxic mechanisms underlying arsenic toxicity. The impact of lower and higher levels of arsenic on the rat brain dopaminergic system, which controls cognitive functions, emotions, motivations, movement, and other functions, has been evaluated (Chandravanshi et al. 2014). Arsenic exposure (2 or 4 mg/kg body weight) increased ROS formation by 47% or 84%, increased oxidative stress, decreased the mitochondrial membrane potential (ΔΨm) by 13% or 15%, and decreased the activity of mitochondrial complexes. Disrupted expression of pro- and anti-apoptotic proteins and stress markers has also been observed. Rats exposed to a lower dose (2 mg/kg body weight) of arsenic tended to recover from both behavioral and neurochemical alterations following the withdrawal of arsenic exposure. Conversely, behavioral and neurochemical changes in rats exposed to a higher dose of arsenic were found to persist for a longer period.

Increased oxidative stress due to arsenic exposure is manifested by a lipid peroxidation process accompanied by inflammation, atherosclerosis, increased DNA damage, death of brain cells, and degeneration of the central nervous system (Felix et al. 2005).

Individuals exposed to acute high doses of arsenic (more than 2 mg of As/kg per day) may develop the destruction of axonal cylinders over a longer period, which may result in peripheral neuropathy (Uede and Furukawa 2003).

Acetylcholine is a neurotransmitter that is important for learning, memory, attention, and other functions. Acetylcholinesterase is an enzyme that hydrolyzes acetylcholine and is an essential component of cholinergic neurotransmission. In rats, arsenic trioxide decreases the activity of acetylcholinesterase in a dose-dependent manner (Patlolla and Tchounwou 2005). The suppressed activity of acetylcholinesterase has been associated with peripheral neuropathy and damage to the central nervous system (Singh et al. 2011).

Epidemiological studies reported a strong correlation between arsenic exposure and cognitive dysfunction in children and adults (Tyler and Allan 2014). These cognitive defects depend on the concentration and duration of arsenic exposure as well as other factors. Children are usually exposed to arsenic for shorter durations than adults are (e.g., occupational exposure); therefore, adults are usually affected more severely than children. In addition, the arsenic methylation capacity is associated with the detoxification process; it is usually greater in children than in adults (Chowdhury et al. 2003).

Animal studies revealed that cognitive dysfunction following arsenic exposure involves cholinergic, glutamatergic, and monoaminergic signaling; altered function of the hippocampus; glucocorticoid signaling; and other mechanisms (Tyler and Allan 2014).

#### Arsenic and cardiovascular diseases

Chronic exposure to arsenic has been associated with the incidence of cardiovascular diseases (Navas-Acien et al. 2005; Stea et al. 2014). Epidemiological studies have confirmed a causal relationship between the level of arsenic exposure and cardiovascular disease risk factors (Moon et al. 2012). Hypertension, one of the major risk factors for cardiovascular disease, has been found to correlate with arsenic exposure in a dose-dependent manner (Abhyankar et al. 2012).

As discussed above, oxidative stress is a well-established mechanism of arsenic-induced toxicity in many organ systems, including the cardiovascular system, also known as the circulatory system, which consists of the heart, blood vessels, and blood. Arsenic-induced ROS formation includes the oxidation of arsenite(III) to arsenate(V) accompanied by the formation of hydroxyl radicals, superoxide radicals, peroxyl radicals, and nitrogen-reactive species (RNS) (Flora 2011). In addition, ROS are also formed by the arsenic-mediated stimulation of an electron-transporting membrane-located enzyme complex, NADPH oxidases (NOXs), or arsenic-induced release of the free iron pool from the iron-storage protein ferritin (Shi et al. 2004). This process may catalyze the decomposition of hydrogen peroxide to hydroxyl radicals via the Fenton reaction and have a synergistic damaging effect with ascorbic acid (Jomova et al. 2024, 2023, 2011).

Nitric oxide synthase (NOS) is a family of enzymes responsible for the synthesis of nitric oxide (NO^**·**^) from L-arginine. Arsenic inhibits endothelial NOS (eNOS) synthase, a major protection barrier of endothelial cells against vascular diseases (Förstermann and Münzel 2006). Low levels of nitric oxide in blood vessels are associated with endothelial dysfunction, which may lead to (i) narrowed blood vessels resulting in hypertension, (ii) inflammation of arteries transporting oxygenated blood from the heart, and (iii) platelet formation, which may cause blood clots and semisolid/gel masses to form in arteries and veins and is important in the control of bleeding. However, blood clots (thrombi) in the circulatory system may cause thrombosis, acute lung embolic events, and heart attacks.

In addition to eNOS, arsenic can inhibit redox enzymes such as superoxide dismutase (SOD), catalase (CAT), glutathione peroxidase (GPx), and thioredoxin reductase (TRX), which in turn increase the level of ROS, such as superoxide radicals, which can subsequently decrease the level of nitric oxide through their mutual (very fast) reaction, producing toxic peroxynitrite (see also below) (Jomova et al. 2024).

Arsenic-induced inhibition of eNOS reduces the bioavailability of NO^·^ and causes endothelial dysfunction, which in turn increases blood pressure (Owlya et al. 1997). Arsenic can also decrease vascular relaxation capacity under in vitro conditions, thus affecting smooth muscle cells (Lee et al. 2003). However, when considering increased blood pressure due to arsenic poisoning, one should consider the possibility of arsenic-mediated renal damage, which may be a contributing factor (Chen et al. 2011).

Arsenic also has direct cytotoxic effects on cardiomyocytes, which are the cardiac cells responsible for heart contraction and relaxation (Luong and Rabkin 2009). Arsenic may induce apoptosis or even necrosis of cardiomyocytes. The most sensitive target for arsenic toxicity is the myocardium, the muscular layer of the heart (Roman et al. 2011). Notably, As_2_O_3_ is a relatively safe chemotherapeutic agent for the treatment of acute promyelocytic leukemia (APL). Cardiotoxicity is a typical side effect of various chemotherapies, and patients receiving As_2_O_3_ should be under the control of a cardiologist (Mathews et al. 2013).

**Fig. 5:**
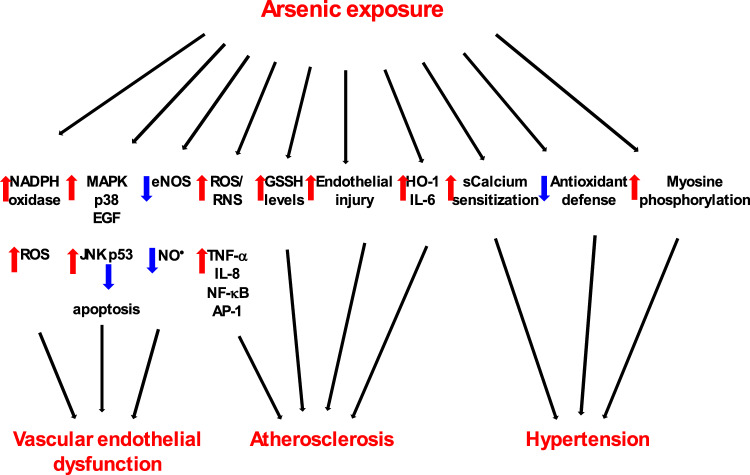
Effects of arsenic on the mechanisms of vascular endothelial dysfunction, atherosclerosis, and hypertension

The extracellular matrix (ECM) is a network of extracellular macromolecules, enzymes, collagen, and other biomolecules that provide support for surrounding cells. Arsenic significantly suppresses the expression of extracellular matrix (ECM) transcripts in the heart and the embryonic mouse fibroblast line NIH3T3, particularly those associated with elastin and collagen (Hays et al. 2008). These data confirmed a tight link between arsenic(III) toxicity and defects in the production of the components of the vascular extracellular matrix. The alleviation of the abovementioned pathologies caused by arsenic toxicity may prevent the incidence and progression of a wide range of cardiovascular diseases. Arsenic-induced biochemical changes and their effects on vascular endothelial dysfunction, atherosclerosis, and hypertension are shown in Fig. 5.

## Mercury

**Fig. 6:**
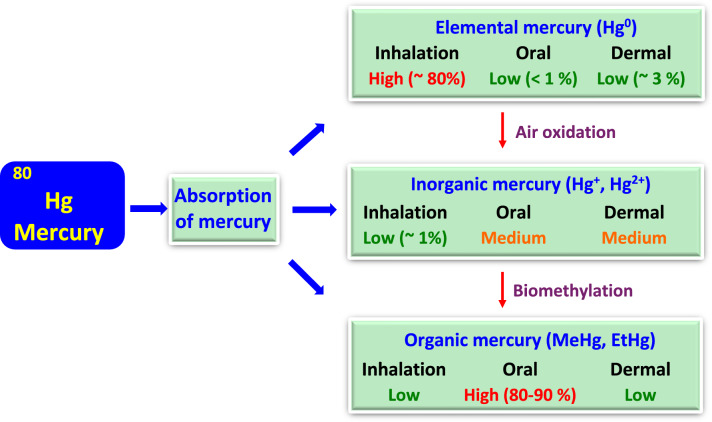
Relative levels of common mercury exposure routes

Mercury (Hg) is the 80th element in the period table. The electronic configuration of mercury is [Xe] 4f^14^5d^10^6s^2^. Mercury is found in rock in the Earth’s crust in several chemical and physical forms, such as elemental (metallic) mercury, inorganic mercury, and organic-methyl mercury (Fig. 6) (Genchi et al. 2017). At room temperature, mercury is in liquid form and weakly volatile (with a partial pressure of 0.261 Pa at 25 °C); however, at higher temperatures, its volatility increases (Gaffney and Marley 2014).

Oxidation states + 1 or + 2 characterize mercury compounds. Compounds with an oxidation number of + 2 are more common. Mercury compounds containing coordinated oxygen (e.g., HgO) are formed at temperatures reaching 350 °C. Mercury compounds with an oxidation state of + 1 are rare. The mercury ion Hg_2_^2+^ exists as a stable diatomic molecule.

The best-known mercury compound is probably HgCl_2_. It is a colorless substance with applications in agriculture (fungicide), industry (catalyst in the production of vinyl chloride) and as a precursor of other Hg-based compounds. Another mercury compound is mercury(II) sulfide (HgS). HgS is used as a pigment in plastics, rubber, and paint.

### Mercury in the environment

Humans and animals are exposed in the environment to various forms of mercury, ranging from elemental mercury to inorganic and organic mercury compounds (Fitzgerald and Clarkson 1991). Elemental mercury (Hg°) is a silver-white metal in a liquid state at room temperature that slowly evaporates, forming colorless, odorless, and toxic vapors. The use of mercury in industrial products such as bulbs and thermometers has been discontinued. Elemental mercury is still used to some extent as dental amalgam fillings or “silver fillings”.

Inorganic mercury is abundant in the environment and occurs in the form of minerals and mineral impurities. Inorganic salts of mercury with sulfur or chlorine are found in water, soil, and dust. Environmental emissions of elemental and inorganic mercury originate from coal-fired power stations and from plants that discharge mercury-contaminated water. Skin creams and other cosmetic products made in some countries from contaminated sources may contain mercury compounds, which may represent a potential health risk (Clarkson and Magos 2006). Mercury sulfide (α-HgS), or vermilion red, is used as a red coloring pigment and in tattoo dyes.

Mercury undergoes a series of chemical and physical transformations in nature, during which it reacts with organic substances to form organo-mercury compounds. The most representative example is a highly neurotoxic methylmercury occurring in the environment. Methylmercury and its derivatives are the major sources of organomercury in humans. Exposure to methylmercury occurs via fish and shellfish consumption.

### Mercury metabolism

As already outlined, there are three types of mercury: elemental (liquid mercury), inorganic (liquid mercury), and organic (methylmercury) (Fig. 6). Following exposure, mercury is distributed in several tissues in the body, preferentially in the kidneys.

Elemental mercury at higher temperatures vaporizes and is absorbed primarily by inhalation, and its absorption via the gastrointestinal tract is minimal (Posin et al. 2023). Mercury has lipid-soluble properties and is readily transported into the bloodstream and erythrocytes. Elemental mercury is converted in erythrocytes by catalase to the divalent inorganic form. Since mercury crosses the blood‒brain barrier (BBB), in addition to the central nervous system, it also affects the kidneys (Aschner and Aschner 1990).

Inorganic mercury compounds are toxic and are moderately absorbed through the gastrointestinal tract and via dermal absorption. It accumulates mainly in the kidney, causing damage to this organ. Owing to their rather low solubility, the penetration of inorganic mercury compounds into the central nervous system is restricted. Elemental and inorganic mercury salts are excreted mainly from the kidney and, to a lesser extent, by the gastrointestinal tract.

Organic mercury comes in three different forms: aryl, short- and long-chain alkyl compounds. Organic methylmercury is primarily absorbed by the gastrointestinal tract (mainly from food) and, to a much lesser extent, via dermal and inhalation routes. Exposure to organic mercury results in chronic or even acute toxicity. Alkyl-organic mercury (methylmercury) is lipid soluble and accumulates in the brain, kidney, liver, and skin. Like other forms of mercury, organic mercury also crosses the blood‒brain barrier as well as the placenta, which may lead to spontaneous abortion.

The half-life determines the amount of organic mercury (MeHg or EtHg) accumulated in the body from the regular consumption of fish. The toxicokinetics of MeHg in humans may depend on genetic background, the microbiome of the gut, weight, and other factors (Sauder et al. 1988). The estimated value of the biological half-life of MeHg is t_1/2_ ~ 50 days, but with a wide confidence interval of 30–150 days (Tam and Rand 2024).

### Mechanisms of mercury toxicity

As outlined above, organic mercury compounds are the most toxic forms of mercury, and representative examples include methyl mercury (MeHg) or ethylmercury (EtHg). Organic mercury is lipid soluble and can permeate through biological membranes, which represents the gateway to the intracellular toxicity of mercury (Branco et al. 2021; do Nascimento et al. 2008). The primary target of MeHg-induced toxicity is the central nervous system.

The transmembrane transport of organic mercury compounds into the cytoplasm occurs through a functionally and biochemically specific amino acid transport system. Intracellular mercury can trigger a range of cellular alterations that can induce detrimental effects on tissues and body organs. Methylmercury is a weak electrophile and preferentially binds protein nucleophilic thiol (–SH) and selenol (–SeH) groups (Jomova and Valko 2013). In addition to the –SH and –SeH functional groups, mercury can also interact more weakly with the carboxyl (–COOH), amide (–CONH_2_), and amino (–NH_2_) groups. These interactions can significantly inhibit the activity of proteins and increase the level of oxidative stress, which can damage all important biomolecules, including proteins, lipids, and nucleic acids. In addition, overproduction of ROS can activate a variety of signaling pathways, such as the nuclear factor erythroid 2-related factor 2 (Nrf2) pathway.

#### Thiols and selenols in MeHg-induced oxidative stress and toxicity

ROS-induced oxidative stress has been implicated in various chronic diseases, such as cancer, neurodegenerative disorders, and cardiovascular diseases. Various redox and non-redox metals, including methylmercury, induce excessive ROS formation (Valko et al. 2005). One of the key manifestations of MeHg-induced toxicity is disrupted redox equilibrium, which is tightly associated with cellular thiols and selenols. As outlined above, MeHg can interact with the thiol (–SH) groups of glutathione and selenol (–SeH) groups of antioxidant selenoenzymes such as glutathione peroxidase (GPx) and thioredoxin reductase (TrxR). Selenol groups are more acidic (pK ~ 5.4) than thiol groups and therefore are deprotonated under physiological conditions. Their inhibition by MeHg results in a shift in the equilibrium of the cell redox balance toward a prooxidant state (Antunes et al. 2018).

Glutathione (GSH) is the most abundant (~ mM concentration) and very efficient low-molecular-weight antioxidant that maintains redox equilibrium in human cells (Pei et al. 2023). The indirect antioxidant activity of glutathione is demonstrated by glutathione peroxidase (GPx), which uses glutathione as a reducing agent to catalyze the reduction of H_2_O_2,_ or organic hydroperoxides to water. The direct antioxidant activity of glutathione is ensured by ROS-scavenging mechanisms.

MeHg-induced oxidative stress and neurotoxicity are attributed to glutathione depletion. MeHg can interact with GSH to form a GS-MeHg complex (Ballatori and Clarkson 1982), which significantly suppresses the levels of “active” GSH manifested by increased oxidative stress (Antunes dos Santos et al. 2018).

Epidemiological and animal trials have shown that MeHg is responsible for the significant loss of neuronal cells in the cerebellum (responsible for movement and balance) and the visual cortex (a cerebral cortex region important in the processing of visual information) (Aschner and Syversen 2005). Among other factors, cell loss can be attributed to low levels of GSH (Kaur et al. 2007). The intracellular concentrations of GSH in the mammalian cerebellum are in the millimolar range; therefore, MeHg-induced oxidative stress can be attributed to direct ROS formation in addition to the direct interaction between MeHg and GSH (Ni et al. 2011).

Glutathione peroxidase and thioredoxin reductase are selenoenzymes containing selenium (Se) atoms in their active sites. The glutathione peroxidase (GPx)/glutathione (GSH) system is one of the most effective defense barriers against oxidative stress. The activity of either of these two components is negatively affected by MeHg, which in turn is responsible for the impaired detoxification of hydrogen peroxide and lipid peroxides. Increased levels of hydrogen peroxide can, in turn, lead to increased formation of ROS, including hydroxyl radicals (Fe, or the Cu-Fenton reaction) or peroxyl radicals capable of causing significant levels of damage to biomolecules (Brigelius-Flohé and Maiorino 2013).

Several different models involving the mouse brain, mouse brain mitochondria, and human neuroblastoma SH-SY5Y cells exposed to MeHg confirmed significantly reduced activity of glutathione peroxidase, impaired cell viability, and increased oxidative stress (Farina et al. 2009).

A study using an in vitro model of human neuroblastoma (SH-SY5Y) cells revealed that MeHg inhibited the activity of the selenoenzymes GPx and TrxR (Meinerz et al. 2017). These results show that selenoenzymes play important roles in cellular detoxification and protection against MeHg intoxication.

The expression of antioxidant genes encoding the GPx and TrxR families is downregulated by MeHg and rescued by elevated levels of selenium (Penglase et al. 2014). This finding indicates that selenium can reduce the toxicity of MeHg; however, the exact mechanism has not yet been explored.

#### MeHg and calcium homeostasis

Mercury can affect calcium homeostasis, which plays an important role in the regulation of neuronal cell death (Kim and Sharma 2003). Mercury compounds can inhibit voltage-gated calcium channels involved in a variety of cellular Ca^2+^-mediated signaling processes and maintain a link between electrical signals and cellular processes of nonelectrical origin, such as contraction. Methyl mercury disrupts calcium homeostasis, which is manifested by increased intracellular calcium levels and subsequent cell death (Choi 1992). In vitro data confirmed that calcium channel blockers (e.g., cardiac drugs used to regulate blood pressure) may delay the methylmercury-induced increase in intracellular calcium stores, and thus prevent neurological disorders related to methylmercury intoxication (Levesque and Atchison 1991).

Another study reported that in addition to increasing intracellular calcium [Ca^2+^]_i_, methyl mercury potently decreased the production of nitric oxide (NO^**·**^) as well as the NO-synthase mRNA level (Kuo et al. 2002). The L-type calcium channel blocker verapamil antagonized the inhibitory effect of methylmercury on nitric oxide production. It has been proposed that the inhibitory effect of MeHg on nitric oxide production is to some extent mediated by the Ca^2+^-stimulated adenylyl cyclase-cAMP protein kinase pathway.

#### MeHg and mitochondria

Mitochondria are frequent targets of mercury compounds. Cerebellar granule cells are numerous cells in the cerebellum that target MeHg-mediated neurotoxicity (Castoldi et al. 2000). MeHg (5–10 µM) impaired mitochondrial activity and led to breakdown (lysis) of the plasma membrane, resulting in necrosis. Shortly after intoxication, lower concentrations of MeHg (0.5–1 μM) did not cause alterations in mitochondrial activity; however, the cells progressively underwent damage, reaching apoptosis 18 h after intoxication. Neural network fragmentation and microtubule depolymerization resulting in altered cellular morphology were detected at a low MeHg concentration (1 μM) within 1.5 h of intoxication. These results indicate that even low MeHg concentrations (0.5–1 μM) cause mitochondrial damage several hours after exposure; conversely, neuronal damage is observed within 1.5 h of intoxication.

In vivo studies reported that MeHg accumulation inside mitochondria triggered a series of biochemical alterations (Denny and Atchison 1994) resembling those observed in mitochondrial respiratory chain inhibition (do Nascimento et al. 2008).

High levels of MeHg in rats decrease the activity of enzymes such as superoxide dismutase (SOD), cytochrome c oxidase, and the heterotetrameric protein succinate dehydrogenase, which are associated with mitochondrial energy metabolism (Yoshino et al. 1966).

Proteins in rat liver mitochondria interact with MeHg and trigger the uptake of K^+^ into mitochondria, which in turn results in the loss of membrane potential, the major cause of uncoupling (Sone et al. 1977). In addition to altering K^+^ homeostasis, treating rat brain mitochondria with MeHg caused both ATP-dependent and ATP-independent decrease in Ca^2+^ uptake and increase in Ca^2+^ efflux from mitochondria (Denny et al. 1993). Notably, relatively small alterations in Ca^2+^ related to depolarization of mitochondria are insufficient to affect neurotransmitter release via normal physiological mechanisms. This fact does not exclude significant changes in other mechanisms triggered by MeHg-induced changes in Ca^2+^ concentrations.

#### MeHg and neurotransmitters

Mercury-induced neurotoxicity is a well-established phenomenon (Branco et al. 2021). An association between mercury and neurotransmission suggests that MeHg can stimulate the spontaneous release of neurotransmitters such as GABA, dopamine, acetylcholine, and serotonin from rat brain synaptosomes (Juarez et al. 2002). This can lead to depolarization or hyperpolarization of the postsynaptic cell.

The neurotoxic effect of methylmercury on mouse cerebral neuron cultures has been studied (Park et al. 1996). Methylmercury chloride (Me–Hg–Cl) caused significant neuronal damage and death in a time-dependent manner. Low-molecular-weight antioxidants such as glutathione, selenium, or cysteine and the antioxidant enzyme catalase were able to reverse the neurotoxicity caused by methylmercury chloride. The N-methyl-D-aspartate (NMDA) receptor is a glutamate receptor and Ca^2+^ ion channel in neurons that mediates a slow component of excitatory synaptic transmission and plasticity. Noncompetitive dizocilpine (MK-801), competitive D-2-amino-5-phosphonovaleric acid (D-AP5), and glycine site 7-chlorokynurenic acid antagonists blocked methylmercury-induced neurotoxicity in cerebral neuron cultures. These results confirmed that ROS and the excitatory potency of acidic amino acids such as glutamate are involved in methylmercury-induced neurotoxicity in cerebral neuron cultures.

The neurotoxicity of MeHg is documented by decreased levels of the intracellular antioxidant glutathione (Shanker et al. 2001). Since cysteine is an important precursor of glutathione synthesis, the effect of MeHg on cysteine uptake by astrocytes and neurons was studied. One hour of pretreatment with MeHg caused significant concentration-dependent inhibition of cysteine uptake by astrocytes but not by neurons. An inhibitory study revealed that the inhibition of cysteine uptake by MeHg in astrocytes occurs through a Na^+^-independent ASC system that is responsible for the transport of small amino acids and the Na^+^- and K^+^-dependent amino acid transport system X-AG.

#### MeHg and the Nrf2 signaling pathway

Nuclear factor erythroid 2-related factor 2 (Nrf2) is an important antioxidant transcription factor that is activated in response to altered redox homeostasis (Cuadraro et al. 2019). Nrf2 regulates the expression of more than 250 genes. Kelch-like ECH-associated protein 1 (Keap1) regulates the activity of Nrf2 in response to oxidative stress and promotes its degradation in the absence of stress. Thus, the Keap1‒Nrf2 pathway is a complex protective response to oxidative and electrophilic stresses. Considering that Nrf2 signaling plays an important role in maintaining redox equilibrium, its involvement in the protection against oxidative stress induced by heavy metals, including mercury, has a rational basis (Buha et al. 2021).

Two months of treatment with inorganic mercury (HgCl_2_) significantly suppressed the accumulation of Nrf2 in the nucleus of cardiac tissue (Baiyun et al. 2018). In addition, the concentration of malondialdehyde (MDA), a biomarker of the lipid peroxidation process, significantly increased. Conversely, a significant decrease in the reduced-to-oxidized glutathione (GSH/GSSG) ratio was observed. This finding highlights the significantly increased level of HgCl_2_-induced oxidative stress.

In a hepatotoxicity study, rats were exposed to HgCl_2_ for 3 days (Liu et al. 2017). Dose-dependent hepatotoxic effects include increased ROS formation, non-protein sulfhydryl, malondialdehyde (MDA), and apoptosis. In addition, HgCl_2_ exposure impaired the antioxidant enzymes GPx and SOD and upregulated Nrf2 and heme oxidase-1 (HO-1) in the liver.

In conclusion, Nrf2 signaling is an important instrument in the defense against heavy metal toxicity. However, Nrf2 signaling may act as a double-edged sword. Some works have concluded that heavy metal toxicity results in increased activity of Nrf2 via a Keap1-dependent mechanism, thus protecting against metal-induced toxicity. However, some studies reported the downregulation of Nrf2 expression due to exposure to heavy metals, including mercury compounds. The most critical parameter affecting the switch of Nrf2 from upregulation to downregulation is the length of heavy metal exposure. Most short-term exposure studies are consistent with Nrf2-mediated protection mechanisms against heavy metal toxicity. Conversely, long-term (chronic) exposure to heavy metal toxicity may lead to hyperactivation of Nrf2, which is associated with increased cell division and ultimately carcinogenesis. Overactivation of Nrf2 can help cells escape oxidative stress-related damage either by increasing the expression of antioxidant target genes or promoting cell proliferation and survival.

### Health effects of mercury

Human exposure to even small amounts of mercury may cause serious health problems, especially the development of children in utero and during infancy, childhood, and early and middle adolescence (Posin et al. 2023). As already discussed above, humans are most often exposed to methylmercury (organic form), which frequently occurs in relatively high amounts in fish and shellfish; however, the risk of exposure to elemental mercury also exists. Mercury toxicity affects the lungs, skin, eyes, and nervous, cardiovascular, and excretory systems. Symptoms of mercury poisoning include a deteriorated nervous system, impaired motor system, impaired hearing, speech, tremors, headaches, and other symptoms.

#### Mercury and diseases of the central nervous system

Mercury vapor (vapHg°) enters the body through the lungs, and approximately 80% of mercury vapor reaches the bloodstream (Magos 1997). vapHg° can cross the blood‒brain barrier, enter the central nervous system, and accumulate predominantly in the cerebellum and cerebral cortex (Warfvinge 1995). Mercury vapor crosses the placenta and can be a threat to the fetus. In addition, mercury vapor is secreted in breast milk; therefore, children breastfed by mothers who live in contaminated areas are at health risk.

Methyl mercury (MeHg) is formed in the aquatic environment and accumulates in fish bound to protein cysteine thiol groups. Ingested fish protein containing the MeHg-cysteine complex is absorbed by the gastrointestinal tract and distributed throughout the body to all major organs within 1–2 days, where it is bound to hemoglobin in erythrocytes (Mottet et al. 1997). MeHg‒cysteine complexes readily cross the blood‒brain barrier and accumulate in the central nervous system. The molecular changes responsible for the incidence of neurological disorders are outlined in Table 1.

**Table 1:** Toxic effects of mercury: molecular alterations affecting the development of neurological disorders

Mercury toxicity	
Molecular alterations	Neurological disorders
ROS-induced oxidative damage	Amyotrophic lateral sclerosis (ALS)
Mitochondrial dysfunction/swelling	Parkinson diseases
ROS and RNS generation	Alzheimer’s disease
Peroxidation of lipids	Cognitive deficits
Enhanced apoptosis	Axonal atrophy
Epigenetic alterations	Impaired motor coordination
Altered membrane fluidity	Mood disorders
Genetic mutations	Mental disease

#### Mercury and glutamate neurotransmission

The glutamate neurotransmitter system is the primary excitatory drive, and its integrity is crucial for the proper functioning of the central nervous system (Branco et al. 2021). A dysregulated glutamate system has been associated with altered glutamate excitation, which is implicated in neurodegenerative pathologies (Simunkova et al. 2019) such as stroke and mental diseases such as schizophrenia and Parkinson’s, Alzheimer’s, and Huntington’s diseases (Crupi et al. 2019).

Both organic mercury (MeHg) and inorganic mercury (Hg^2+^) affect glutamate-mediated excitatory signaling by increasing its release from presynaptic terminals and simultaneously hampering glutamate uptake by astrocytes, resulting in dysregulation of the glutamate system (Aschner et al. 1990).

#### Mercury and oxidative stress-related diseases

The brain is very sensitive to oxidative stress-induced damage, mainly because of its high and specific metabolic activity. High iron pools, the abundance of a variety of lipid structures, and high oxygen consumption lead to the formation of a complex system prone to oxidative damage. Oxidative damage to the central nervous system is characterized by increased lipid peroxidation, inflammation, DNA and protein damage, autophagy, and other pathophysiological events. The analysis of biomarkers of oxidative damage confirmed that oxidative stress is a common denominator of various neurodegenerative diseases, such as Alzheimer’s disease, Parkinson’s disease, Huntington’s disease, and mental diseases (Poprac et al. 2017).

Among mercury compounds, methylmercury is the most common cause of neurological alterations. MeHg interacts with major antioxidant selenoenzymes, such as glutathione peroxidase (GPx), thioredoxin reductase (TrxR), and the major intracellular antioxidant glutathione (GSH). The decreased activity of enzymes due to mercury intoxication is reflected by increased ROS-induced oxidative stress and the disruption of redox signaling pathways, which negatively affects neurodevelopment (Branco et al. 2012). Oxidative stress causes mitochondrial damage, which in turn results in more damage to astrocytes than to neurons. MeHg has adverse effects on neurodevelopment manifested by the suppressed differentiation of neural stem cells into neurons (Tamm et al. 2008).

MeHg decreases the inner mitochondrial membrane potential and increases the formation of superoxide radicals and hydrogen peroxide. MeHg inhibits the electron transport chain, which, in turn, induces the release of cytochrome c, causing the death of brain cells (Mori et al. 2011).

#### Mercury and neuroinflammatory disorders

Neuroinflammation plays a pivotal role in the incidence, development, and progression of neurodegenerative diseases such as multiple sclerosis, Alzheimer’s disease, Parkinson’s disease, Huntington’s disease, and other disorders that significantly affect the brain and spinal cord (Adamu et al. 2024).

MeHg-induced neurotoxicity is accompanied by neuroinflammation characterized by activated glial cells, mainly microglia and astrocytes. In vitro and in vivo studies revealed that MeHg exposure triggers the expression of numerous proinflammatory cytokines (Muniroh 2020; Ni et al. 2012). Low MeHg concentrations (up to 5 µM) activate interleukin-6 (IL-6) and monocyte chemoattractant protein (MCP)-1 expression in the human astrocytoma cell line U87MG (Clarkson et al. 2007; Muniroh 2020). Monocyte chemoattractant protein-1 (MCP-1) is a member of the chemokine family that attracts and activates potent chemokines for monocytes and macrophages at the site of inflammation. The phenomenon of macrophage activation was further studied in the range of 2–10 µM concentrations of MeHg. The results of this study confirmed the activation of murine (RAW 264.7) and human (U937)-derived macrophage lines via the upregulation of the expression of macrophage inflammatory protein-2 (MIP-2), IL-6, and IL-8 (David et al. 2017; Yamamoto et al. 2017).

The mechanism of MeHg-induced cytokine expression involves the induction of oxidative stress, cyclic adenosine monophosphate (cAMP), and cytosolic phospholipase A2 and C (Shanker et al. 2004; Takanaga et al. 2004). MeHg-induced cytokine expression in astrocytes involves excessive formation of hydrogen peroxide and activation of transcription factors such as nuclear factor kappa B (NF-κB) and nuclear factor-erythroid related factor-2 (Nrf-2) (Kim et al. 2012). MeHg has been shown to activate Nrf2 by altering the expression of NADPH-quinone oxidoreductase 1 (NQO1) (a xenobiotic-metabolizing enzyme that detoxifies chemical stressors) and glutamate-cysteine ligase, also known as gamma-glutamylcysteine synthetase (first and rate-limiting enzyme of glutathione biosynthesis) (Muniroh 2020).

#### Mercury and Alzheimer’s disease

The potential link between mercury and Alzheimer’s disease (AD) has been the subject of various studies (Azar et al. 2021). No difference in mercury content in the cerebrospinal fluid was detected between AD patients and controls; however, the mercury level in the blood of AD patients was greater than that in the blood of controls (Gerhardsson et al. 2008). However, several studies evaluating blood mercury content have reported contradictory results (Azar et al. 2021). There was no difference in the mercury content in the urine between AD patients and the control group.

AD is a disease of multifactorial origin characterized by (i) enhanced dysregulated redox metal (Cu, Fe)-catalyzed production of ROS and oxidative stress, (ii) the presence of abnormal structures, amyloid plaques (consisting of extracellular deposits of amyloid-β (Aβ) protein) and neurofibrillary tangles (abnormal accumulation of the protein tau), and (iii) acetylcholine deficiency caused by the disturbed (aberrant) activity of acetylcholinesterase. Mercury-induced oxidative stress in the brain has been discussed in the preceding paragraphs. Mercury inhibits the activity of various enzymes, which may result in the induction of oxidative stress.

Mercury has been found to elicit inhibitory effects on kinase enzymes, which is manifested by an increase in the β-secretase pathway of amyloid precursor protein (APP) metabolism, resulting in increased formation of amyloid-β (Olivieri et al. 2000).

Wistar rats were administered MeHg for 4 weeks, and the total Hg content in the blood and brain regions and the levels of amyloid-β in the plasma, cerebrospinal fluid, and brain regions were evaluated (Kim et al. 2014). In addition, the expression of the multifunctional cell surface low-density lipoprotein receptor-related protein 1 (LRP1) in the brain capillary endothelium was determined. The results revealed that in the MeHg-treated group, amyloid-β levels decreased in the cerebrospinal fluid (CSF) and increased in the hippocampus in a dose-dependent manner. MeHg also decreased the expression of LRP1 in the brain capillary endothelium, in agreement with the decreased level of amyloid-β in the cerebrospinal fluid. These results indicate that MeHg inhibits the transport of amyloid-β and supports its accumulation in the hippocampus. The results also revealed that the plasma level of LRP1 may be an early and sensitive biomarker of mercury-induced accumulation of amyloid-β in the brain.

Tau protein (tubulin-associated unit) is a protein that plays an important role in the development of Alzheimer’s disease (Muralidar et al. 2020). Tau is a microtubule-associated protein that is abundant in the axons of neurons. Microtubules are formed by dimerization of α- and β-tubulins. The hyperphosphorylated microtubule-associated protein tau is a main component of neurofibrillary tangles. It has been reported that methylmercury-induced tau hyperphosphorylation is caused by the activation of the c-Jun-N-terminal kinase (c-JNK) signaling pathway in the cerebral cortex (Fujimara et al. 2009). MeHg interacts with the thiol groups of both α- and β-tubulins, thus disturbing the microtubule structure, and inorganic mercury (Hg^2+^) interacts with tau fragments, causing microtubule disassembly (Bjørklund et al. 2017).

To explore in greater detail the etiological involvement of mercury toxicity in the pathogenesis of Alzheimer’s disease, more epidemiological trials studying the incidence of Alzheimer’s disease among individuals occupationally exposed to mercury are necessary. A benefit would be the screening of mercury content in serum and cerebrospinal fluid during regular medical checkups in risk groups. Trials should consider age, sex, pregnancy, the occurrence of chronic disease, and other pathophysiological factors to obtain reliable data on the extent to which mercury toxicity can contribute to the incidence of Alzheimer’s disease.

#### Mercury and cardiovascular disease

As discussed above, the toxic effects of mercury are most often related to the central nervous system. However, mercury is also known to cause cardiotoxicity (Hu et al. 2021). Methylmercury intoxication is associated with a suppressed low-frequency component of the heart rate variability spectrum and an increased risk of myocardial infarction, hypertension, atherosclerosis, and other cardiac pathologies (Genchi et al. 2017).

The lower limit of mercury in hair, above which there is a risk of cardiovascular diseases, is estimated to be ~ 2 μg/g (Hu et al. 2020). This finding is in agreement with an interesting correlation between hair levels of mercury and oxidized low-density lipoproteins (LDLs) (Yoshizawa et al. 2002; Virtanen et al. 2005).

Excessively high levels of LDL in the blood may cause cholesterol to be deposited in artery walls and form plaques in a process called atherosclerosis (Borén et al. 2020). Atherosclerosis manifests as the hardening of arteries, which can cause infarction, stroke, blood clots, or aneurysms. Mercury-increased LDL oxidation disrupts the integrity of the mitochondrial phospholipid membrane, resulting in the loss of mitochondrial potential and the externalization of phosphatidylserine, which is considered an early event leading to apoptosis. This process is manifested by cardiac dysfunction and heart failure (Wu et al. 2024).

Mercury interferes with the homeostasis of intracellular calcium, which may affect the contractility of myocytes. Myocarditis involves inflammation of the heart muscle and can be worsened by mercury, which activates proinflammatory signaling, such as nuclear factor kappa-light-chain-enhancer of activated B cells (NF-κB) (Rice et al. 2014).

#### Mercury interference with cancer

A correlation between occupational exposure to mercury and the potential risk of cancer has been reported (Skalny et al. 2022). However, the results of epidemiological studies evaluating the link between mercury and cancer are not unambiguous. Experimental models revealed that high-dose mercury exposure was associated with cytotoxicity, whereas low-dose exposure interfered with estrogen receptor, JNK, and Nrf2 signaling and NADPH oxidase, supporting the proliferation of normal and cancerous cells. Following low-dose mercury exposure, suppressed apoptosis, enhanced survival signaling, and inhibited DNA repair mechanisms lead to increased DNA damage and the risk of malignant transformation.

A statistically significant correlation between the number of farmers who used fungicides containing mercury and leukemia occurrence in the Cracow region (southern Poland) was reported (Janicki et al. 1987). The mercury content in the farmers’ hair was 1.24 mg/kg, whereas that of healthy subjects was 0.49 mg/kg.

#### Mercury and renal diseases

In addition to characteristic symptoms typical of “general” mercury toxicity, such as tremor, excitability, muscle weakness, impaired speech, and other conditions, renal damage also manifests as edema, proteinuria (high levels of proteins in the urine), changes in urine volume, and other symptoms.

The severity of renal disease is correlated with the amount of intoxicated mercury. Inorganic mercury accumulates in the kidneys as a Hg-cysteine complex at a metal-to-ligand ratio of 1:2 in the form of a Cys-S-Hg-S-Cys complex. The Hg-cysteine complex can be reabsorbed by organic transporters (Wu et al. 2024).

One of the most prominent epigenetic alterations caused by mercury intoxication involves histone posttranslational modifications and DNA methylation. MeHg-treated animals presented significantly altered renal function substantiated by epigenetic changes in DNA from kidney tissue (Khan et al. 2019). These changes involve aberrant alterations in the methylation of proteolysis matrix metalloproteinase 9, further promoting cytoskeleton disruption and subsequent damage to renal function. It has been reported that the exposure of mouse embryonic stem cells to inorganic mercury (HgCl_2_) reduces the total number of histone proteins, which is attributed to a decrease in the mono-methylation of the 27th amino acid in histone H3 (H3K27) (Ghadia et al. 2012).

#### Mercury and lung disease

The pulmonary system is unusually vulnerable to mercury intoxication because approximately 80% of mercury vapor can enter the lungs and cause lung inflammation (pneumonitis), difficulties breathing, cough, asthma, and chest pain, resulting in progressive pulmonary failure (Bjorklund et al. 2018). The lungs are the entrance gates for the distribution of mercury throughout the body. Mercury-mediated pulmonary diseases are related to mercury vapor (Hg°), which can be easily absorbed by the lungs. Mercury inhalation can negatively affect immunological responses through altered adaptive and innate immunity (Miao et al. 2023).

Mercury causes damage to the epithelial lining, a part of the alveolar membrane involved in gas exchange (Todd et al. 2012). Mercury-mediated tissue damage can promote pulmonary fibrosis, which is characterized by excessive accumulation of extracellular matrix and airway remodeling, resulting in lung scarring and shortness of breath.

Pulmonary toxicity mediated by mercury can affect other organ systems associated with the lungs. Thus, preventing toxicity caused by mercury vapors is of key importance. A recent study reported adverse respiratory effects in individuals whose hair mercury content was greater than 5 ppm, whereas concentrations less than 1 ppm seem to be safe (Pateda et al. 2018).

## Lead

Lead (Pb) is the 82nd element in the periodic table of elements and is a very soft, gray-blue metallic element. Lead has 82 electrons with the following electronic configuration: [Xe]4f^14^5d^10^6s^2^6p^2^ (Fuhr et al. 1996; Needleman 2004). The main lead mineral is galena (PbS), which contains 87 wt% lead. Lead is a good conductor of electrical current and occurs in compounds in the + 2 and + 4 oxidation states, with the + 2 oxidation state being more stable. Pb^2+^ compounds form a wide range of coordination geometries with coordinated carboxylates, thiolates, nitrogen, phosphorus, halides, and other ligands. The coordination numbers of Pb^2+^ range from 1 to 12. Fourfold coordination in a tetrahedral arrangement around a central atom is observed only for Pb^4+^ compounds with bulky ligands.

Lead is a highly toxic element for humans, animals, and the environment. Lead accumulates in bones, liver, and kidneys and is particularly hazardous for children, young people, and pregnant women (Wani et al. 2015).

### Lead in the environment

Lead persists in the environment and accumulates in soils and sediments through air absorption, waste discharge to the water system, mining activities, and erosion (Wani et al. 2015). Lead has various adverse effects on an ecosystem, affecting plants, terrestrial animals, and aquatic animals. In addition to other adverse effects, lead toxicity can retard the reproduction and growth of animals and plants. With various regulations, including the removal of lead from petrol, lead levels in the air in the past 40 years have significantly decreased.

Many products used in households, including paint, plumbing materials, pipes, glasses, batteries, cosmetics, and ammunition, contain lead or lead compounds (WHO 2017). Much of human exposure to lead originates from industrial sources, such as fossil fuels, mining sites, smelting sites, and other sources. The settling process of lead particles to the ground, which are released into the air from artificial sources, is rather slow; therefore, inhalation represents one of the key routes of intoxication by lead. Lead is exposed to the atmosphere in the form of lead sulfates, oxides, and carbonates.

Lead is dangerous to children because growing organisms have a greater capacity to absorb more lead than adults do. Pregnant women are of particular concern because lead exposure can harm their developing baby. Current US regulations set blood lead limits at 3.5 µg per deciliter of blood for children aged 1–5 years (ACCLPP 2012).

### Absorption, distribution, and metabolism of lead

Lead has no positive physiological effect on the human body. Humans are exposed to lead via several routes: the digestive system, pulmonary system, and small amounts via the dermal route (Rădulescu and Lundgren 2019). The respiratory tract very efficiently absorbs lead particles of submicron size. Larger particles can be transported from the respiratory tract to the gastrointestinal tract by swallowing. The efficiency of lead absorption by the gastrointestinal tract depends on various factors, such as an individual’s weight, physical status, and age. A significantly greater absorption of lead is observed in children than in adults. Inorganic lead is primarily absorbed in the first part of the small intestine (duodenum). Dermal absorption of lead is less effective and restricted to organic lead. Inorganic lead is distributed throughout the body in the same way regardless of the route of absorption. Pb in the blood is transported into erythrocytes by an anion exchanger (Bergdahl et al. 1997; Smith et al. 2002; Simons 1985). Pb efflux from erythrocytes occurs via active ATP transport. Pb in erythrocytes interacts with several intracellular proteins, such as aminolevulinic acid dehydratase (ALAD). Human ALAD is a polymorphic zinc-containing enzyme with two alleles (ALAD1 and ALAD2) and three genotypes. ALAD is a major lead-binding site in red blood cells (Xie et al. 1998). Zinc is known to inhibit the detrimental effects of Pb and provide protection and reactivation of ALAD activity.

Inorganic Pb in plasma exists in several forms: (i) loosely bound to low-affinity sites in serum albumin or other transport proteins; (ii) complexed with low-molecular-weight ligands, including amino acids, carboxylic acids or antioxidants; and (iii) bound to metalloproteins or (iv) free (unbound) Pb^2+^ (Al-Modhefer and Bradbury 1991). Pb^2+^-coordinating ligands involve sulfhydryl-containing molecules, histidine, glutathione, citrates, and other compounds with heavy metal chelating properties.

Approximately 90% of lead is stored in mineralized tissues, including teeth and bones. The majority of lead in soft tissues is stored in the liver. The lead concentration in bones tends to increase with age. Postmortem analysis revealed that bones accumulate approximately 94% of the total adult body lead burden (Barry 1975). In bones, Pb^2+^ can replace Ca^2+^ from the Ca^2+^–O = P–phosphate groups of hydroxyapatite and form stable complexes with phosphate groups (Pb^2+^–O = P–). The exchange of Pb between bones and soft tissues has been documented by Pb isotope analysis, which revealed that 10–90% of the blood lead may originate from the bone stock (Gulson 2000). In addition to pregnancy, the release of lead to blood from bone is related to osteoporosis, abnormal weight loss, and lactation.

Quantitative analysis of Pb in soft tissues performed in the mid-1970s revealed that adults contain approximately 20 µg/dL Pb (Barry 1975). However, more recent data from the U.S. revealed concentrations of less than 5 µg/dL (CDC 2018). Most of the Pb in soft tissue can be found in the liver, accounting for 33%, followed by skeletal muscle (18%), skin (16%), connective tissue (11%), fat (6%), kidney (4%), lung (4%), and brain (2%) (Schroeder and Tipton 1968). Another quantitative analysis revealed that the highest concentrations of lead in soft tissues are found in the liver and kidney (Barry 1975).

The mechanism by which lead is absorbed by soft tissues is not entirely clear. Two saturable and non-saturable pathways for Pb absorption have been proposed. In these pathways, Pb uses transport mechanisms designed for iron and calcium, involving voltage-gated L-type calcium channels in adrenal medullary cells and store-operated calcium channels in embryonic kidney and brain endothelial cells (Kerper and Hinkle 1997a, b) and possibly anion exchangers in astrocytes. Divalent metal transporter 1 (DMT1) is expressed in the kidney and small intestine and is tailored for iron transport. More research is needed to clarify whether DMT1 is used for the transport of Pb across the renal epithelium. In many soft tissues, lead is bound to proteins such as retinol-binding proteins (Fowler and DuVal 1991).

The distribution of organic Pb (e.g., tetraethyl and tetramethyl lead) has rarely been studied. One hour after 1–2 min of inhalation of tetraethyl Pb or tetramethyl Pb (1 mg/m^3^), the liver accounts for approximately half of the lead burden, the kidneys 5%, and the rest of the body has a burden that is widely distributed (ATSDR 2020). Pharmacokinetic analysis of organic lead in blood revealed that the organic form of lead was first distributed from the respiratory tract, followed by a distribution of dealkylated Pb compounds (Vural and Duydu 1995).

The metabolism of inorganic lead is characterized by the formation of lead complexes with low-molecular-weight ligands and/or lead complexes with coordinated ligands from proteins. The most populated ligands involved in the interaction with Pb are albumin and non-protein sulfhydryl groups. The major intracellular ligand in erythrocytes is aminolevulinic acid dehydratase (ALAD) (see above).

Organic lead (alkyl Pb) is metabolized in the liver by oxidative dealkylation catalyzed by cytochrome P-450. Tetraethyl Pb is excreted in the urine as ethyl Pb, diethyl PB, and inorganic Pb (Turlakiewicz and Chmielnicka 1985; Zhang et al. 1994). Following exposure to tetraalkyl Pb compounds, trialkyl Pb metabolites were detected in the liver, kidney, and brain (Nielsen et al. 1978). Inhaled 0.64 mg of tetraethyl Pb/m^3^ and 78 mg of tetramethyl Pb/m^3^ were removed from the blood within 10 h.

Regardless of the route of exposure, lead is excreted predominantly in urine and feces and to a lesser extent in the saliva, sweat, and seminal fluids (Hernández-Ochoa et al. 2005). Urinary excretion of Pb is double that of fecal excretion.

### Mechanisms of lead toxicity

One of the first papers studying lead-induced toxicity was published in 1965 and focused on the oxidation of unsaturated fatty acids by a series of metals, including Pb^2+^ (Willis^1965^). Unlike redox-active metals, lead was reported to be ineffective under the given experimental conditions. Years later, a more detailed study confirmed a direct link between lead and ROS-induced lipid peroxidation in several rat brain regions. The maximum retention concentrations of lead in the spinal cord and cerebellum correlate in a dose-dependent manner with the levels of peroxidation of lipids (Ur-Rehman 1984), including apoptotic pathways (He et al. 2003).

**Fig. 7:**
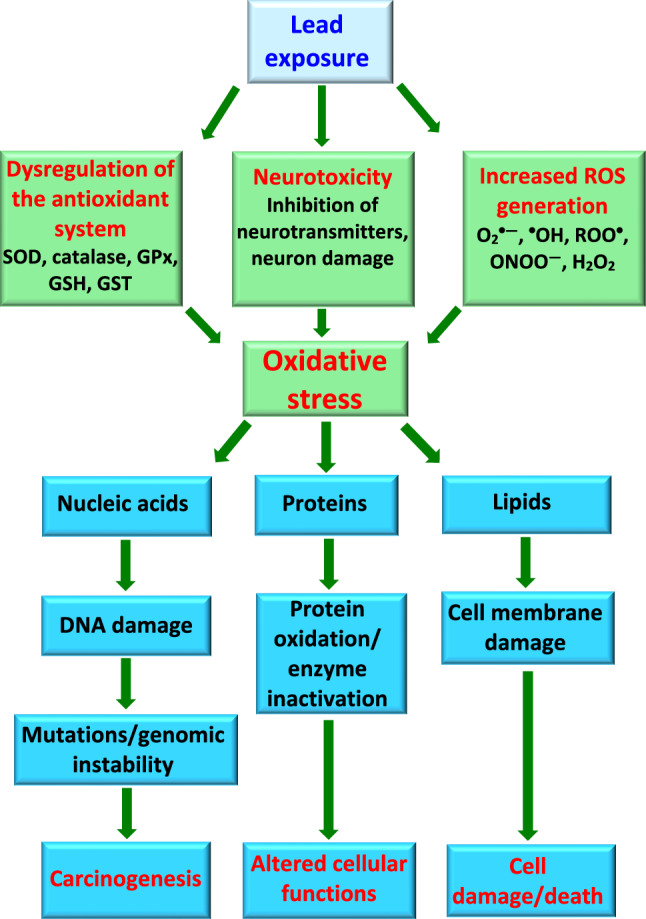
A simplified scheme of the molecular targets and mechanism of lead toxicity

The mechanisms of lead toxicity are complex and involve a variety of biomolecules, processes, interference with various signaling pathways, and oxidative stress, which is a common denominator of many pathologies induced by lead exposure (Fig. 7).

#### Lead and neurotoxicity

According to Dr. Silbergeld, lead has two distinct forms of neurotoxicity. The first is a toxic neurodevelopmental agent that interferes with the differentiation of the central nervous system, and the second is a neuropharmacological toxicant that affects the ionic mechanism of neurotransmission (Silbergeld 1992; Virgolini and Aschner 2021). Thus, Pb^2+^-induced neurotoxicity is multifactorial and occurs through several distinct mechanisms, including lead-induced oxidative stress, divalent cation mimicry, mitochondrial dysfunction, increased neuroinflammation, and enhanced brain permeability.

*Lead and oxidative stress:* Oxidative stress is an imbalance between the generation and elimination of ROS/RNS in favor of their generation. Oxidative stress causes damage to all important molecules, such as DNA, proteins, and lipids (Forman and Zhang 2021). The brain is extremely susceptible to oxidative damage due to high oxygen consumption, which is produced in mitochondrial neurons. The neurotoxicity of lead underlines its ability to cross the blood‒brain barrier (Singh et al. 2018).

Pb^2+^ exposure results in dysregulation of the antioxidant pool. Lead interacts with protein thiol (–SH) groups, which, in turn, inhibits the activity of antioxidant enzymes such as glutathione peroxidase (GPx), superoxide dismutase (SOD), and catalase (CAT) and dysregulates their efficiency as ROS scavengers. Pb^2+^ can share its electrons and form covalent bonds with protein sulfur atoms because of the polarizability of both the sulfur and lead atoms. Inhibiting the activity of antioxidant enzymes in the mouse liver resulted in increased ROS formation and oxidative stress (Xu et al. 2008).

In addition to enzymes, lead can interact with the cysteine-SH groups of reduced glutathione (GSH) and induce a toxic response (Hultberg et al. 2001). Thus, lead exposure can cause damage to the antioxidant defense system and suppress the overall antioxidant capacity of the liver, which is the most important detoxification organ of the body.

The brain is highly abundant in lipids, and increased ROS formation in the brain is manifested by increased peroxidation of lipids, as confirmed by increased biomarkers of the lipid peroxidation process (Jomova et al. 2023). This process is catalyzed by the presence of Fe^2+^ stored in ferritin. Fatty acids and iron combine, resulting in increased peroxidation of lipids and the formation of toxic substances involved in lipid peroxidation, such as the toxic and mutagenic metabolites malondialdehyde (MDA) and 4-hydroxynonenal (HNE). Fe^2+^ can also catalyze the dismutation of hydrogen peroxide, a major signaling molecule, and form damaging hydroxyl radicals (Fenton reaction). Increased ROS formation oxidizes neurotransmitters, particularly catecholamines. Dopamine metabolism by monoamine oxidases produces hydrogen peroxide, a substrate for the Fenton reaction.

These events contribute to the formation of a prooxidant environment in the cell, enhancing the transport of Pb^2+^ across the blood–brain barrier (BBB), which causes damage to important intracellular biomolecules in neuronal cells. The activation of proinflammatory mediators and the dysregulation of tight junction proteins and their associated adhesion factors are characteristics of these mechanisms.

Microglia, as part of their protective immune response, elicit the production of superoxide radicals and hydrogen peroxide; however, neurons and oligodendrocytes are more susceptible to ROS-induced oxidative damage than are microglia and astrocytes (Wang and Michaelis 2010).

Other major mechanisms of lead toxicity manifested by oxidative stress as a common denominator include lead-hemoglobin interactions involving aminolevulinic acid (δ-ALA) dehydratase inhibition (Warren et al. 1988). Lead interferes with several steps of heme synthesis and inhibits aminolevulinic acid dehydratase (ALAD), an enzyme encoded by the *ALAD* gene that catalyzes the second step of porphyrin biosynthesis. The inhibited activity of *ALAD* results in increased blood and plasma levels and consequently urinary excretion of aminolevulinic acid (ALA). Aminolevulinic acid undergoes conversion from the keto form to the enol form required for autooxidation reactions, generating superoxide radical (O_2_^**·**−^) and reducing ferrichrome. Simultaneously, the reaction of aminolevulinic acid with the oxidized form of hemoglobin (Oxy-HB) produces aminolevulinic radicals and hydrogen peroxide. The formation of superoxide and hydrogen peroxide can generate hydroxyl radicals (^·^OH).

In addition, ferrochelatase (FECH), which is expressed by the FECH gene and is a final enzyme of the heme biosynthesis pathway that inserts iron into protoporphyrin IX, is inhibited by lead (Desnick et al. 2021). A screening test for lead poisoning revealed that the free erythrocyte protoporphyrin (FEP) concentration is elevated in cases of lead intoxication, iron deficiency, and other diseases.

*Lead and the mechanism of ionic mimicry:* Another mechanism of significant health threat and lead toxicity involves the substitution of essential elements in the metal binding sites of proteins with lead. Pb^2+^ can mimic the effects of structurally similar divalent metal ions such as Ca^2+^ and Zn^2+^ and interfere with functions requiring the presence of these essential metals (Vetter and Leclerc 2003; Jomova et al. 2022).

Pb^2+^-induced increase in intracellular calcium ([Ca^2+^]_i)_ triggers mitochondrial dysfunction and oxidative damage, thus promoting the apoptosis of neuronal cells (Virgolini and Aschner 2021). In addition, Pb^2+^ negatively affects the transport and storage of Ca^2+^, interferes with protein calcium binding sites, or alters cell functions that ensure calcium homeostasis. The ionic radius of Pb^2+^ is 1.19 Å, and the atomic radius of Ca^2+^ is 1.00 Å, which makes these metal ions mutually substitutable (Emsley 1998). Pb^2+^ can replace not only Ca^2+^ from their natural binding sites, but also structurally similar Zn^2+^, Fe^2+^, or eventually Cu^2+^ ions. Replacement of essential metals from their natural metal-binding sites by Pb^2+^ results in a shifted redox balance and impaired organelles, cells, and organ systems.

Two important proteins whose function is affected by Pb^2+^ are calmodulin, a Ca-binding multifunctional calcium-binding messenger protein, and protein kinase Cα (PKC), a family of serine/threonine protein kinases that regulate various cellular functions, including Ca-mediated signal transduction (Goodsell 2016). The effect of Pb^2+^ on the peripheral membrane-binding domain C2α of protein kinase Cα (PKC) has been studied via NMR and isothermal titration calorimetry techniques (Morales et al. 2011). The study revealed that the C2α domain binds Pb^2+^ with higher affinity than the natural cofactor Ca^2+^. Förster resonance energy transfer spectroscopy revealed that Pb^2+^ can displace Ca^2+^ from the C2α domain in the presence of lipid membranes and inhibit the activity of protein kinase Cα (PKC).

Other examples of the ability of Pb^2+^ to interfere with essential metals involve (i) voltage-gated calcium channels (VGCCs), also known as voltage-dependent calcium channels, which are present in the membrane of neurons, glial cells, and other excitable cells. Pb^2+^ can block all types of voltage-gated calcium channels, which prevents the physiological increase in intracellular Ca^2+^ levels (Atchison 2003). (ii) The GluR2 subunit of the α-amino-3-hydroxy-5-methyl-4-isoxazolepropionic acid (AMPA) receptor is involved in cell migration, calcium signaling, and normal functioning of the central nervous system. It has been reported that long-term exposure to lead acetate, Pb(CH_3_COO)_2_, suppresses GluR2 expression, resulting in neuronal cell death (Ishida et al. 2013). (iii) Another target of Pb^2+^ toxicity is the N-methyl-D-aspartate receptor (NMDAR), a glutamatergic receptor and Ca^2+^ ion channel present in neurons. The NMDA receptor allows the anti-port transport of Ca^2+^ and Na^+^ into the cell and of K^+^ out of the cell. In addition to blocking NMDARs, Pb^2+^ toxicity manifests as an inhibitory effect on the transport of sodium, calcium, and other ions through the membrane, thus suppressing the excitatory postsynaptic potential, which in turn may limit the generation of action potentials (Flora et al. 2013).

*Lead and neuronal mitochondrial dysfunction:* Mitochondria are organelles that generate the chemical energy necessary to power cellular processes. The toxic effects of metals that target mitochondria have been frequently studied (Meyer et al. 2013). Although non-redox metals do not directly participate in redox reactions, Pb^2+^ can disturb mitochondrial redox homeostasis by suppressing the antioxidant activity of reduced glutathione (GSH) or antioxidant enzymes (mtSOD (SOD2), catalase, glutathione peroxidase) as a result of the interaction of Pb^2+^ with protein -SH groups. In addition, Pb^2+^ can replace Ca^2+^, Fe^2+^, or Zn^2+^, thus altering the integrity of membranes and promoting the lipid peroxidation process (Caito and Aschner 2015) and the catalytic activity of traces of redox-active metals to decompose hydrogen peroxide via the Fenton reaction. ROS-mediated damage to mitochondrial organelles may disrupt redox homeostasis and cause cell death (Blajszczak and Bonini 2017).

Importantly, Pb^2+^ interferes with the production of nitric oxide (NO^**·**^), an important messenger involved in the regulation of the immune, cardiovascular, and nervous systems (Calabrese et al. 2007). Structural similarities and mutual substitution of Ca^2+^ with Pb^2+^ can alter the activity and expression of Ca^2+^-dependent neuronal nitric oxide synthase (nNOS) and endothelial NOS synthase (eNOS) in various brain regions (Garcia-Arenas et al. 1999; Nava-Ruiz et al. 2012).

Pb^2+^ can compete with and displace Zn^2+^, an important structural element of NOS enzymes that produces NO^**·**^ at the glutamate/N‐methyl‐D‐aspartate receptor (NMDAR)/nitric oxide (NO^**·**^) signaling pathway (Garza-Lombó et al. 2018). The impairment of the NO^·^ transduction system may have an impact on the plasticity and synaptic development of brain regions related to higher cognitive functions such as abstract thinking, problem-solving, systematic decision-making, and many more.

It has been reported that Pb^2+^ affects oxidative processes in mitochondria. Low levels of Pb^2+^ can alter phosphorylation in neonatal rat brain mitochondria (Bull et al. 1979) and mitochondrial respiration in the synaptosomes of young rats (Rafalowska et al. 1996).

Lead toxicity is accompanied by increased ROS-induced oxidative stress, which, in turn, induces DNA damage. DNA damage activates p53 and triggers an imbalance in the Bax/Bcl-2 ratio and irreversible cell death. The mechanism of Pb^2+^ toxicity involves altered mitochondrial permeability, the release of mitochondrial proteins resulting in the apoptosis of brain cells, an increase in the proapoptotic Bax/Bcl-2 ratio via the downregulation of the antiapoptotic protein Bcl-2, and the upregulation of the proapoptotic protein Bax (Pulido and Parrish 2003; Sharifi et al. 2002).

In a study employing PC-12 cells, which are frequently used in neurobiological studies, the mechanism of toxicity revealed that Pb^2+^ can trigger apoptosis through the altered ratio between the antiapoptotic protein BCl-2 and the proapoptotic proteins Bax and p53 in favor of their proapoptotic activity (Xu et al. 2006).

Apoptosis is associated with disturbed calcium homeostasis in intracellular compartments and is regulated by mitochondrial functions (Garza-Lombó et al. 2018). Ryanodine receptors (RyRs) are bulky ion channels that release Ca^2+^ from an intracellular calcium-storing compartment and play important roles in brain function. Intoxication by Pb^2+^ affects ryanodine receptors, thereby increasing [Ca^2+^]_I_ in cultured cells (Fan et al. 2013; Jia et al. 2018). Increased cytoplasmic [Ca^2+^]_I_ supports Ca^2+^ uptake by transmembrane mitochondrial uniporter calcium channels. These processes alter the mitochondrial membrane potential (MMP) and reduce the synthesis of ATP, causing oxidative injury.

*Lead and neuroinflammation: *Neuroinflammation is a complex process involving activated microglia and astrocytes, the production of inflammatory cytokines, increased ROS generation, diminished antioxidant capacity, neuronal loss, and injury to the central nervous system (Chibowska et al. 2016; Metryka et al. 2018).

Pb^2+^ exposure triggers the activation of nuclear factor kappa B (NF-κB), activator protein-1 (AP-1), c-jun N-terminal kinase (JNK), and caspases in the rat brain, which may contribute to neurotoxicity via apoptotic mechanisms (Ramesh et al. 2001).

Chronic exposure of immature rats to Pb^2+^ resulted in astroglial activation and microglia-induced neuroinflammation accompanied by increased production of proinflammatory cytokines such as the interleukins IL-1β, IL-6, and the tumor necrosis factor TNF-α (Struzynska et al. 2007). Developmental Pb^2+^ intoxication in mice induces the expression of transforming growth factor-β1 (TGF-β1) and the interleukin IL-6 (Kasten-Jolly and Lawrence 2018).

Pb^2+^ exposure increases the production of the proinflammatory cytokines interleukin IL-1β, tumor necrosis factor TNF-α and interferon-gamma IFN-γ which activate the synthesis of NADPH oxidases (NOXes), the major nonmitochondrial source of superoxide O_2_^·−^ in microglia and astrocytes (Kasten-Jolly and Lawrence 2018). Pb^2+^ also activates the synthesis and secretion of the proinflammatory cytokine IL-8 through a nuclear factor erythroid 2-related factor 2 (Nrf2)-dependent mechanism involved in the synthesis of xenobiotic-metabolizing enzymes (Metryka et al. 2018).

*Lead and DNA damage:* In workers prone to occupational exposure to Pb, the content of Pb in whole blood has been monitored via ICP‒MS, and DNA damage in leukocytes has been reported (Danadevi et al. 2003). The mean Pb abundance was nine times greater in the studied group than in the control group. In addition, the study revealed nearly 45% more DNA damage in the exposed group than in the control group.

The increased level of DNA damage in workers exposed to lead is attributed to the excessive formation of ROS (and increased oxidative stress) due to the interaction of Pb^2+^ with sulfhydryl groups of intracellular glutathione (GSH) and antioxidant enzymes such as SOD, catalase, and glutathione peroxides (GPx).

Female rabbits were studied for DNA damage and tissue apoptosis after prolonged low-dose (15 mg/kg) and high-dose (30 mg/kg) oral exposure to lead acetate, Pb(CH_3_COO)_2_. (Ahmed et al. 2012). The results revealed that lead acetate causes dose-dependent DNA damage and apoptosis in rabbit tissue, as reflected by degenerative changes and diffuse atretic follicles in ovarian tissues.

### Health effects of lead toxicity

Lead poisoning occurs over months or years of exposure (ATSDR 2020). The most vulnerable are children under 6 years of age, who can suffer permanent adverse health effects. Intoxication by high levels of lead in adults can be fatal. Symptoms of lead intoxication can include weight loss, hypertension, headaches, abdominal pain, neurological and mental problems, kidney problems, reproductive problems, gastrointestinal problems, cardiovascular disorders, and deterioration of other organ systems.

#### Lead toxicity and neurological disorders

Lead is considered a neurotoxin that affects the nervous system most significantly of all organ systems (Gilani et al. 2015). The half-life of lead in the brain is 2 years, whereas the half-life in the blood is 1 month (ATSDR 2020).

Astrocytes, a highly abundant subtype of glial cells, neurons, and the extracellular matrix, have a blood–brain barrier (BBB) that is permeable to Pb^2+^. Adverse health effects are diagnosed in adults and children at blood lead levels of 5 μg/dL (Collin et al. 2022).

The levels of lead in children are usually higher than those in adults, mainly because children are often exposed to the same environment as adults are and consume proportionally more food relative to their weight than adults do (Hauptman et al. 2017). Lead exposure is especially dangerous to the developing brains of children and fetuses. Increased levels of lead in the blood correlate with delayed growth in children, decreased cognitive function and IQ, and hyperactivity. Children with increased blood lead levels have ADHD, hearing problems, and a damaged peripheral nerve system. Some studies have confirmed that increased blood levels of lead may disturb brain functions, termed encephalopathy.

Lead intoxication is associated with damage to various brain regions and manifests as cognitive decline, altered executive functions, abnormal social behavior (e.g., aggression), and problems with fine motor skills (Ramírez Ortega et al. 2021).

Neurological disorders caused by lead are closely connected with the role of calcium in the brain (Goldstein 1993). Calcium is essential for mediating communication between neurons by releasing synaptic vesicles. Brain-derived neurotrophic factor (BDNF), which is active in synapses where communication between cells occurs, plays a key role in this communication. The action potential allows a transiently increased calcium concentration at the presynaptic active zone, which triggers the release of neurotransmitters. Lead poisoning is characterized by its transport to target tissues and competition with structurally similar calcium, which may disrupt the calcium-dependent production of brain-derived neurotrophic factors and consequently connections between brain cells.

Lead also affects the function of multifunctional serine/threonine protein kinase C (PKC), which is important in modulating various cellular processes, including exocytosis, apoptosis, proliferation, and other processes (Zhao et al. 1998). Lead interacts with PKC more tightly than with its physiological activator, calcium, which may cause problems with neurotransmission. Dysregulation of protein kinase C may also affect cellular second-messenger systems, which may negatively affect gene expression and protein synthesis.

#### Lead toxicity and the renal system

The WHO estimated that lead toxicity accounts for approximately 3% of kidney disorders (Angrand et al. 2022). Nephrotoxicity results from the fact that the kidney is the main route of removing lead from the body. Lead-induced toxicity is manifested by loss of kidney function accompanied by altered fluid homeostasis as a result of a decreased glomerular filtration rate and activation of the renin–angiotensin system, promoting fluid retention with various pathological consequences, such as peripheral edema and pulmonary edema.

Although the threshold value of the blood lead level above which kidney damage occurs has not been established exactly, many studies have reported a direct link between lead exposure and renal dysfunction. Adult epidemiological studies revealed that blood lead levels of approximately 5 µg/dL are associated with adverse effects on kidney function. Lead-induced nephrotoxicity is manifested by several pathologies, such as proximal tubular nephropathy, resulting in cell death and the formation of tubular glomeruli (Walsh and Unwin 2012). Another pathology associated with lead-induced toxicity is glomerulosclerosis, characterized by the scarring of the nephron filters (glomerulus) attached to a tubule, causing increased urine protein levels (Rosenberg and Kopp 2017).

Depending on the time and dose of lead exposure, renal abnormalities are classified as acute or chronic nephropathy. Acute nephropathy is characterized by tubulointerstitial renal fibrosis, a hallmark of chronic kidney disease, and a predictor of renal survival. Tubulointerstitial renal fibrosis is characterized by a harmful process of progressive deposition of connective tissue in the kidney parenchyma, resulting in the deterioration of renal functions (Efstratiadis et al. 2009).

Lead-induced damage to the lining cells of the proximal tubular system results in the formation of nuclear inclusions and ultimately renal glycosuria and aminoaciduria, which are characterized by high levels of sugar and amino acids in the urine, respectively (Kellum et al. 2021).

The levels of lead exposure correlate with the blood levels and clearance rates of creatinine, indicating the severity of impaired renal function. The biomarkers of acute kidney injury involve the urinary level of *N*-acetyl-β-D-glucosaminidase, which is a proximal tubule lysosomal enzyme and indicator of acute kidney injury. Another biomarker, kidney injury molecule-1 (KIM-1), is a transmembrane glycoprotein that is upregulated in proximal tubular cells in response to acute nephrotoxic injury. Under physiological conditions, kidney tubular epithelial cells produce low levels of the matrix metalloproteinases MMP-2 and MMP-9; however, following lead intoxication and renal fibrosis, the mRNA transcription levels of MMP-9 and MMP-2 are significantly increased and serve as sensitive biomarkers of acute kidney injury (Cheng et al. 2017; Wiercinska et al. 2011).

#### Lead toxicity and the reproductive system

Lead exposure has been associated with many types of physiological and biochemical damage to the reproductive system (Llave and Pacheco 2020). In men, exposure to inorganic lead has been associated with altered parameters of semen quality, sperm morphology, reduced sperm count, and motility. In women, lead exposure may affect fertility, pregnancy complications, pregnancy hypertension, low-birthweight, and many other factors.

The replacement of histones with protamines in sperm DNA protects sperm chromatin. Protamines are specific proteins rich in arginine, and their altered expression may negatively affect male fertility (Simon et al. 2011). Zinc interacts with human protamine P2 (HP2), thus stabilizing sperm chromatin. Since HP2 has a similar affinity for both Pb^2+^ and Zn^2+^ and intoxicated Pb^2+^ can compete with/replace Zn^2+^ in HP2, the strength of the HP2‒DNA interaction can be hampered. In addition, the interaction of Pb^2+^ with DNA may negatively affect the HP2–DNA interaction. Thus, exposure to lead resulting in altered HP2‒DNA interactions negatively affects sperm chromatin condensation and male fertility (Quintanilla-Vega et al. 2000).

Like zinc, lead can also displace calcium because of its structural similarities. Lead crosses the placenta, and its interference with calcium metabolism may impair the growth of the fetus (Zhu et al. 2010). In addition, pregnancy hypertension (Yoon and Ahn 2016), premature rupture of membranes (Huang et al. 2018), preeclampsia (high blood pressure, high protein in the urine, swelling of legs and feet, headaches, nausea, and liver problems), and other negative symptoms (Disha et al. 2019) may occur.

*Lead toxicity and cardiovascular disorders:* Lead toxicity is associated with the following cardiovascular disorders: peripheral vascular disease, ischemic heart disease, and stroke. Several clinical studies have reported an association between chronic lead exposure and hypertension; however, other factors contributing to elevated blood pressure, such as age, weight, diet, and family history, should be considered (Vaziri and Gonick 2008).

Low-to-moderate levels of lead (~ 30 µg/dL) are considered relatively safe as far as blood pressure is concerned; however, higher amounts of lead (mostly occupational exposure) may represent a potential risk for hypertension.

A dose-dependent association between the severity of cardiovascular disease and lead exposure has been reported (Navas-Acien et al. 2007). In addition to hypertension, some studies employing the general population and relatively low blood lead levels (< 5 μg/dL) reported positive correlations with stroke mortality, peripheral arterial disease, and coronary heart disease. When a link between hypertension and lead intoxication is considered, impaired renal function and other pathologies affecting blood pressure should be considered.

In vitro and in vivo studies using experimental animals revealed that the main factors responsible for the lead-induced increase in blood pressure are oxidative stress, deficiency of nitric oxide (NO^**·**^), inflammation, and dysregulated coronary vasoregulation mechanisms such as coronary perfusion pressure, tissue pressure, and metabolic and endothelial factors (Vaziri 2008). The lead-induced formation of ROS (e.g., hydrogen peroxide) may participate in the Fenton reaction, producing hydroxyl radicals (^·^OH), which may cause severe damage to cardiomyocytes (Cai and Harrison 2000).

It has been proposed that lead-mediated reduction in nitric oxide availability is not a consequence of its reduced production but rather of oxidative stress. Depletion of nitric oxide is a consequence of its interaction with superoxide radical, which produces, according to the diffusion-controlled reaction, peroxynitrite (ONOO^−^) (Kirsch and Groot 2002)

$${\text{NO}}^{ \cdot } + {\text{O}}_{2}^{ \cdot - } \to {\text{ONOO}}^{ - } {\text{ k}} = \left( {4 - 19} \right) \times 10^{9} {\text{ M}}^{ - 1} {\text{s}}^{ - 1}$$

Peroxynitrite is a highly cytotoxic RNS that contributes not only to adverse cardiovascular but also to the renal and neurological consequences of lead exposure.

Lead-induced hypertension in rats resulted in the upregulation of the gp91^phox^ subunit of NAD(P)H oxidase in the left ventricle and the brain and compensatory upregulation of superoxide dismutase, Mn-SOD (SOD2) in the heart and Cu,Zn-SOD (SOD1) in the brain and kidney. Interestingly, no changes in catalase or glutathione peroxidase (GPx) levels have been observed (Farmand et al. 2005). The relatively constant levels of the H_2_O_2_-converting enzymes catalase and glutathione peroxidase on one side and increased oxidative stress on the other can result in the accumulation of hydrogen peroxide (Vaziri et al. 2003). Hydrogen peroxide is a substrate for the Fenton reaction, forming highly reactive hydroxyl radicals and a potent activator of nuclear factor kappa B (NF-κB), producing proinflammatory cytokines, chemokines, and adhesion molecules and contributing to cardiovascular remodeling.

## Chromium

Chromium (Cr) is the 24th element in the periodic table. Chromium is a transition metal element with an electronic configuration [Ar]3d^5^4s^1^ containing one electron in each of the 3d orbitals (Anger et al. 2000). Owing to its electronic configuration, chromium can exist in oxidation states ranging from -2 to + 6. Chemically and biologically, the most important states are 0 (elemental chromium), + 3 (trivalent chromium), and + 6 (hexavalent chromium) (Wise et al. 2022).

### Chromium in the environment

Chromium is a metal that naturally occurs in rocks, volcanic emissions, water, and soil. Chromium occurring in the ore is predominantly in the trivalent state as Cr^3+^; however, in the course of industrial processing, chromium can either be reduced to elemental chromium Cr^0^ or oxidized to hexavalent chromium Cr^6+^ (Wise et al. 2022).

Millions of industrial workers worldwide, including those involved in chrome plating, steel welding, and other chrome-related industries, are exposed to welding fumes that frequently contain chromium (Reif and Murray 2024). The chromium concentration in the air in urban and contaminated areas ranges from 10 ng/m^3^ to 450 ng/m^3^. Many workers in industry are exposed to Cr^3+^ and Cr^6+^ as soluble or insoluble materials. The general population is at risk of chromium intoxication through inhaling air, ingesting food, and drinking water contaminated with chromium.

### Absorption, distribution, and metabolism of chromium

Chromium can enter living organisms via inhalation, the oral route, and dermal contact. Acute poisoning most frequently occurs through the oral route, whereas chronic poisoning occurs via inhalation and dermal contact (Alvarez et al. 2021). Severe exposures are not typical for occupational or environmental intoxication with chromium and are usually accidental. Inhaled chromium species irritate the respiratory tract, cause pulmonary sensitization, and may increase the risk of lung cancer. Cr(VI) compounds in contact with the skin can cause dermatitis and skin ulcers.

The immediate treatment of acute intoxication by chromium may require hemodialysis to effectively eliminate chromium from the body (ATSDR 2012). Additional therapies involve supplemental oxygen, bronchodilators, expectorants, and ventilatory support. Oral toxicity treatment involves vomiting, lavage, and then the intake of ascorbate, N-acetylcysteine, calcium EDTA, and other metal chelators. Increased intake of small molecular weight antioxidants such as vitamin E, B6, and essential metals (Se, Zn) may help alleviate the adverse effects of chromium toxicity and increase oxidative stress. Dermal toxicity caused by chromium requires the mechanical removal of affected areas and washing.

The severity of intoxication by inhalation depends on the size of the particles. Larger particles (> 5 µm) are usually less soluble and may be more toxic than smaller particles (< 5 µm) absorbed in the bloodstream (Alvarez et al. 2021; Nickens et al. 2010).

The toxicity of Cr(VI) in the form of CrO_4_^2−^ has been well-documented for more than 2 centuries. In addition, hexavalent (VI) chromium compounds have been classified as known or probable human carcinogens. (Alvarez et al. 2021). The toxicity of Cr(VI) species is associated mainly with their ability to increase oxidative stress through various mechanisms (see below).

The absorption capacity of Cr(III) is rather poor, and chromium enters living systems predominantly in the hexavalent form. Cr(VI) species are structurally similar to phosphates and sulfates and, therefore, can enter cells via transmembrane anion channels designed for their transport (Wise et al. 2022; Chakraborty et al. 2022). Cr(VI) in the bloodstream is taken up by erythrocytes and reduced to Cr(III) by a variety of biological reductants, such as small-molecular-weight antioxidants (ascorbate, reduced glutathione (GSH), cysteine) and antioxidant enzymes (superoxide dismutase (SOD, catalase)) (Ma et al. 2024). The reduction process from Cr(VI) to Cr(III) may proceed via pentavalent/tetravalent intermediates (Singh et al. 2022). Ascorbate is a major biological reductant of Cr(VI), accounting for approximately 80%, followed by reduced glutathione or cysteine (the remaining 20%) (Nickens et al. 2010). Notably, the defense against Cr(VI) toxicity already starts in the saliva and the gastric environment by gastric juice and represents an important protective mechanism against oral exposure (Sun et al. 2015). In contrast to hexavalent chromium, trivalent chromium competes with iron and binds directly to transferrin, an iron-transporting plasma protein.

Since the 1950s, trivalent chromium species, Cr(III), have been classified in small amounts as micronutrients important for glucose and lipid metabolism (Shin et al. 2023). However, some studies have reported that Cr(III) species can be potentially toxic and carcinogenic. Safe Cr(III) supplements use chelating agents such as picolinate ligands (Cr(III)-picolinate complexes), which reduce the toxicity of Cr(III) metal ions at a minimum level. This is analogous to the highly toxic metal gadolinium. Gadolinium complexed with suitable ligands has reduced toxicity to a minimum and can be used as a contrast agent in magnetic resonance imaging (MRI).

Cr(VI) can cross the placenta into the fetal circulation. Following inhalation exposure, chromium is found in breast milk (Banu et al. 2017), so lactating women exposed to chromium can put their babies at considerable risk.

Cr(III) is widely distributed within the human body, with the greatest amount being absorbed in the form of a Cr(III)-protein complex via the bone marrow, lungs, kidney, and liver (ATSDR 2012). The highest amount of chromium in all organs was detected in the lungs.

Approximately 60% of an absorbed Cr(VI) dose is excreted predominantly in the form of Cr(III) via the renal system within 8 h of ingestion. Smaller amounts are excreted in nails, hair, sweat, and biliary excretions (Kiilunen et al. 1983). Chromium clearance from plasma is usually rapid and takes several hours; expectedly, tissue clearance is slower and may take several days. A small portion of ingested chromium is excreted via the biliary system.

### Mechanisms of chromium toxicity

Under in vitro conditions, Cr(VI) does not interact with DNA; however, the presence of cellular reductants such as reduced glutathione or ascorbate may cause a variety of Cr-induced DNA lesions and significant levels of oxidative damage.

#### Chromium-induced oxidative stress

As outlined above, Cr(VI) species largely exist in the form of chromate anions (CrO_4_^2−^) and can enter cells via transmembrane anion channels used for the transport of phosphate and sulfate anions (Salnikow and Zhitkovich 2008).

**Fig. 8:**
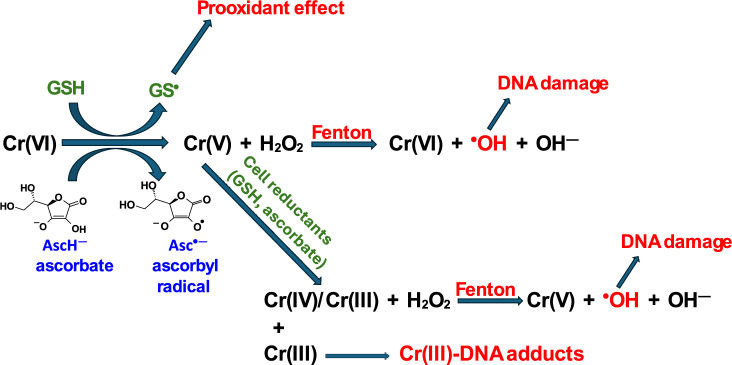
Redox reactions of chromium ions in biological environments

The Cr(VI) inside the cell can undergo a cascade of reactions leading to toxic chromium-based species in the intracellular environment. The most efficient in vivo reductants of Cr(VI) are the cellular detoxifying agents glutathione and ascorbate, which are present at sufficient concentrations in the cell. It is believed that under in vivo conditions, the interaction of Cr(VI) with ascorbate proceeds via a two-electron process and results in the formation of Cr(IV) intermediates (DesMarais and Costa 2019). In addition to ascorbate, the intracellular environment is highly abundant in reduced glutathione (GSH), which interacts with Cr(VI) and slowly reduces hexavalent chromium to the pentavalent species Cr(V) (Fig. 8) (O’Brien et al. 2001). In the rat lung, ascorbate has been found to be kinetically more efficient than glutathione (Standeven and Wetterhahn 1992).

In vitro studies confirmed that in addition to ascorbate and GSH, various other substances can reduce chromates. The most effective reductants are lipoic acid, cysteine, fructose, and other natural reductants (Kasprzak 2002; Liu and Shi 2001).

A specially designed electron paramagnetic resonance (EPR) spectrometer for the in vivo detection of free radicals confirmed the presence of Cr(V) intermediates in the liver and blood as a result of the reduction process of Cr(VI) species in the cellular environment (Liu and Shi 2001). This study convincingly confirmed the involvement of Cr(V) and related free radicals in the mechanism of Cr(VI)-induced toxicity. The reduction process of Cr(VI) species can generate not only Cr(V) but also Cr(IV) species. Biological reductants such as glutathione (GHS) or ascorbate (AscH^−^) reduce Cr(VI) to Cr(V) with concomitant formation of glutathionyl (GS^**·**^) and ascorbyl radicals (Asc^**·**−^), increasing, thus, the oxidative stress. A mixture of both oxidation states of chromium, Cr(V) and Cr(IV), in the presence of a major cellular signaling molecule, hydrogen peroxide, can catalyze the generation of hydroxyl radicals (^**·**^OH) via the Fenton reaction (Fig. 8). Hydroxyl radicals are mediators of oxidative DNA damage. Various adducts of hydroxyl radicals with DNA bases have been detected in cancer tissues; the most representative example is the 8-OH-guanine DNA adduct.

Free radicals and lipid hydroperoxides generated by the decomposition of hydrogen peroxide (Fenton reaction) in the presence of Cr(III) ions were studied via EPR spin trapping spectroscopy (Shi et al. 1993). The application of spin traps confirmed the generation of hydroxyl radicals. Whereas the application of a chelating and contrast agent for diagnostic imaging, diethylenetriaminepentaacetic acid (DTPA) suppressed the formation of hydroxyl radicals, typical antioxidants, such as glutathione and cysteine, did not affect radical formation.

Reduced forms of chromium species, e.g., Cr(III), have been shown to be involved in redox cyclic reactions in the Fenton reaction and as catalysts in the decomposition of hydrogen peroxide, resulting in the formation of hydroxyl radicals (Shi et al. 1993). The toxicity of Cr(III) complexes strongly depends on the environment of the redox metal ions. Cr(III) ions complexed with picolinate ligands neutralize the trivalent charge and reduce the toxicity of chromium ions.

Two systems containing Cr(VI) and ascorbate, differing in the presence and absence of hydrogen peroxide, were evaluated for the level of DNA strand breaks (Shi et al. 1994). Gel electrophoretic measurements revealed that the level of DNA damage was significantly greater in the system containing hydrogen peroxide (Fenton).

Studies with rats following the oral administration of 25 mg of Na_2_Cr_2_O_7_ revealed significantly increased levels of lipid peroxides in liver mitochondria and microsomes (Bagchi et al. 1995). Analysis of rat urine confirmed increased levels of malondialdehyde, formaldehyde, acetone, and acetaldehyde, which are reactive, mutagenic, and toxic products of the lipid peroxidation process.

#### Chromium and signaling pathways

In a study of chromium(VI)-induced hepatotoxicity, hexavalent chromium was reported to activate Kupffer cells, which can release proinflammatory cytokines such as the tumor necrosis factor TNF-α and the interleukins IL-6 and IL-1β and activate the nuclear factor kappa B (NF-κB) signaling pathways (p-IKKα/β and p-p65) (Shen et al. 2023).

Cr(VI) has been reported to activate NF-κB in Jurkat cells, which are frequently used in studies of T-cell leukemia, certain cancers, and HIV (Ye et al. 1995). The proposed mechanism resulting in the activation of NF-κB involves the formation of ROS generated by redox cycling between Cr(V) and Cr(IV) in the Fenton reaction. Cr(VI)-induced activation of a redox-sensitive transcription factor—activator protein (AP-1), has been associated with the phosphorylation of p38 MAP kinase (MAPK) but not with extracellular signal-regulated kinase (ERK). These experiments confirmed that key redox-sensitive transcription factors, NF-κB and AP-1, are activated in response to increased hydroxyl radicals by the Cr(V)/Cr(IV)-mediated Fenton reaction.

P53 is the most frequently mutated gene in human cancers, and the tumor suppressor protein p53 protects cells against tumorigenesis (Chen et al. 2022a, b). This transcription factor is known to be activated by various stimuli, such as ROS, radiation, and toxic substances. A human non-small cell lung cancer cell line (A549) treated with Cr(VI) was shown to activate the protein p53 via the increased formation of hydroxyl radicals (^·^OH) (Wang and Shi 2001). Hydroxyl radicals are generated by the dismutation of hydrogen peroxide in the Fenton reaction, which is catalyzed by traces of pentavalent chromium Cr(V) being reduced from hexavalent chromium by the essential electron donor NADPH. Chromium-induced activation of p53 is associated with phosphorylation of the Ser15 site and acetylation of the Lys382 site.

#### Chromium and genotoxicity

Chromium toxicity is closely associated with its genotoxic effects. Chromium can alter both DNA methylation and histone modification, thus negatively affecting cellular epigenetics. Chromium has been shown to alter the levels of H3K27Me3, H3K4Me3, and H3K9Me3, which epigenetically modify the DNA packaging of the protein histone H3, indicating trimethylation at the 27th, 4th, and 9th lysine residues of the histone H3 protein, respectively (Sun et al. 2009). Altered levels of the histone protein H3 are associated with unfavorable prognoses in several chronic diseases, including cancer.

Mismatch repair (MMR) proteins play an important role in repairing DNA errors (e.g., point mutations). The genotoxicity of Cr(VI) has been associated with increased levels of H3K9Me2 (di-methylation at the 9th lysine residue of the histone H3 protein), which is detected in human non-small cell lung cancer cell lines (A549) (Sun et al. 2009). This resulted in altered expression of the MLH1 protein, a member of the mismatch repair family of proteins involved in the repair of DNA mismatches. Thus, chromium toxicity may negatively affect the DNA repair process.

Histone deacetylases (HDACs) are enzymes responsible for the removal of acetyl groups from the lysine residues of both histone and non-histone proteins (Seto and Yoshida 2014). Cr(VI) can inhibit the activity of these enzymes. Chromium can induce crosslinks between histone deacetylase 1 (HDAC1) and the DNA methyltransferase enzyme (DNMT1), forming the HDAC1-DNMT1 complex. This and other epigenetic alterations in gene expression may represent important stimuli in chromium-induced carcinogenesis (Schnekenburger et al. 2007).

The Cr(III)-dependent pathway associated with the carcinogenicity and mutagenicity of Cr(VI) compounds has been the subject of a series of studies (Zhitkovich et al. 2001). The reduction of the carcinogenic chromate Cr(VI) by biological reductants such as ascorbate or glutathione results in the formation of Cr(III)-ascorbate-DNA crosslinks and binary chromium(III)–DNA adducts in which chromium interacts with guanine at the N7 position (Brown et al. 2020). These two chromium DNA adducts substantiate the mutagenic and genotoxic potential of Cr(VI) species. Whereas ascorbate-Cr(III)-DNA adducts show a high level of mutagenicity, Cr(VI)-DNA adducts exhibit a high degree of genotoxicity and a low level of mutagenicity.

### Health effects of chromium toxicity

As discussed above, chromium bioaccumulation can induce multiple pathophysiological effects, the most common being allergic reactions, gastrointestinal problems, altered function of the male reproductive system, and neurological, cardiovascular, hepatological, and neuronal toxicity. To monitor the extent of intoxication by chromium, blood tests and urine analysis are most frequently used (Franchini and Mutti 1988). Here, we report several examples of the health consequences of various types of chromium-induced toxicity.

#### Chromium and respiratory toxicity

Chromium inhalation is associated with bronchial tissue inflammation, and increased lung clearance results in the incidence of asthma, coughing, and other respiratory problems (Hossini et al. 2022).

Short-term Cr(VI) vapor or particle exposure results in inflammation in lung tissue that typically persists for several weeks. Chronic or repetitive Cr(VI) exposure causes airway neutrophilia, a common feature of many chronic inflammatory lung diseases (Beaver et al. 2009).

Lung inflammation caused by Cr(VI) has been documented in cell culture, animal and human studies, and trials (Kouokam et al. 2022). Respiratory Cr(VI) toxicity has been shown to regulate various inflammatory pathways, such as the MAPK, NF-κB, and Akt pathways.

Evaluations by the Dutch Centre for Occupational Diseases revealed that long-term inhalation exposure (more than 10 years) is manifested by chronic inflammation of the airways leading to bronchitis, lung fibrosis, chronic obstructive pulmonary disease (COPD), and emphysema (Lenderink and Laan 2014).

The chromium-mediated respiratory effects observed in inhalation studies in rodents have been shown to be time- and concentration-dependent. The solubility of chromium particles may significantly affect their toxic effects and the site of their toxic action. Whereas insoluble chromium compounds may cause damage to the lower respiratory tract, exposure to soluble chromium trioxide causes damage to the nasal mucosa and perforation (Derelanko et al. 1999). The association between the solubility and toxicity of chromium compounds was evaluated in another study. This study revealed that the toxicity of chromium oxide (low solubility) is much weaker than the toxicity of chromium sulfate and Cr_2_(SO_4_)_3_ (higher solubility) (ATSDR 2012). Thus, the toxicity of chromium compounds generally increases with their solubility.

#### Chromium and dermal toxicity

A tragic example of unusually severe chromium intoxication has been reported from an electroplating factory where a young worker fell into a tank containing chromates, which has fatal consequences. The victim suffers from skin burns and multiple organ failure (Lin et al. 2009).

The most frequently reported toxic dermal irritants that cause skin burns are hexavalent chromium compounds such as chromium trioxide (CrO_3_), sodium chromate (Na_2_CrO_4_), potassium chromate (K_2_CrO_4_), and dichromate (K_2_Cr_2_O_7_). Skin burns can open an entry route for chromium compounds into organisms and promote systemic toxicity (Jumina and Harizal 2019). Shortly after dermal exposure to potassium chromate, mitochondria-mediated apoptosis of skin fibroblasts may occur (Lee and Goh 1988). Normal cultured human fibroblasts exposed to Cr(VI) induce the activation of an ataxia-telangiectasia mutated (ATM) serine/threonine kinase that plays an important role in cell death in response to genotoxic stresses (Ha et al. 2004). Activated ATM is accompanied by the formation of S-phase-dependent DNA double-strand breaks.

Animal experiments following chromate exposure revealed skin inflammation, edema, and necrosis. A lethal dose of chromium causes scarring and induces full-thickness destruction of skin tissue (ATSDR 2012).

#### Chromium and gastrointestinal toxicity

In general, the intestine can absorb hexavalent chromium compounds more readily than Cr(III) compounds. Gastric juice readily reduces ingested Cr(VI) to Cr(III) species; however, their absorption capacity from the gut is limited to approximately 1% in comparison to Cr(VI) compounds, whose absorption capacity is approximately 10% (Donaldson and Barreras 1966).

Gastrointestinal problems associated with Cr(VI) exposure include abdominal pain, gastritis, duodenal ulcers, and liver disorders such as cirrhosis (Katsas et al. 2024). Practically immediate symptoms of Cr(VI) oral intake include nausea, vomiting, diarrhea, gastric pain, and fever. The oral lethal dose for the average adult is approximately 1–3 g of Cr(VI) compounds.

The relatively low genotoxicity/carcinogenicity observed in animals exposed to Cr(VI) through drinking water has been attributed to extracellular Cr(VI) reduction in the stomach (de Flora 2000).

In another study, the duodenum of a hybrid strain, B6C3F1 mice, which were exposed to varying doses of Cr(VI) in drinking water for 7 or 90 days, exhibited dose- and time-dependent decreases in the reduced-to-oxidized glutathione ratio (GSH/GSSG) (Thompson et al. 2011). These findings suggest that chronic low-dose exposure to Cr(VI) in drinking water can induce oxidative stress in target tissues.

#### Chromium and cardiovascular toxicity

A nearly 2-year-old baby experienced cardiac collapse after consuming an unknown quantity of sodium dichromate. Early hypoxia alterations in the myocardium were discovered after autopsy (Ellis et al. 1982). Another example of chromium-mediated cardiotoxicity, cardiovascular collapse, has been reported a few hours after the consumption of 50 ml of chromic acid by a 35-year-old woman (Loubieres et al. 1999). Ingestion of 50 g of chromium sulfate, (Cr_2_(SO_4_)_3_, caused the death of a woman who died from cardiogenic shock accompanied by bleeding and damage to the gastrointestinal mucosa (Van Heerden et al. 1994). Alterations in myocardial bioelectrical and mechanical activity, as assessed by electrocardiography and cardiac ballistics, were found in a study on the heart function of middle-aged workers exposed to K₂Cr₂O₇ (Rager et al. 2019).

An in vivo study of a subcutaneous injection of potassium dichromate K₂Cr₂O₇ revealed increased oxidative stress, apoptosis, and inflammatory mediators in the Wistar rat heart (Li et al. 2019).

An in vitro experiment using cardiac cells was carried out to examine the effects of exposure to Cr(VI) at various concentrations (4.5–0.25 ppm) for 24 h (Chang et al. 2011). These findings demonstrated a decline in cardiovascular function and reduced RAF kinase inhibitor protein (RKIP) production.

In a different study, the toxicity of Cr(VI) in human umbilical vein endothelial cells (HUVECs) and bovine hemoglobin (bHb) induced oxidative stress and activated the JNK and p38 MAPK cascades, which ultimately resulted in cell death mediated by the mitochondrial apoptosis pathway (Cao et al. 2019). These findings suggest that Cr(VI) may also contribute to cardiovascular toxicity by causing harm to HUVECs and changing the structure of bHb (Cao et al. 2019).

#### Chromium and reproductive toxicity

Following exposure of adult monkey testes to Cr(VI) for 6 months, histopathological and biochemical analyses revealed impaired spermatogenesis and aberrant sperm cell lineage ultrastructures in the nucleus, Golgi, mitochondria, cytoplasm, and one of the most important cells for sperm production, Sertoli cells. Although Cr(VI) disturbed the redox equilibrium manifested by an imbalance between antioxidants and ROS in the treated testes, the negative effects of Cr(VI) on the reproductive system may be alleviated within 6 months after withdrawal (Aruldhas et al. 2005).

The effects of accidental oral exposure of Cr(VI) on the female reproductive system have also been studied. The results have shown that dose-dependent Cr(VI) has deleterious effects on female fertility (Hossini et al. 2022). A reduced frequency of mating, fewer follicles, a rise in abortion, implantation problems, and negative effects on fetuses and litters represent only some of these changes (Banu et al. 2017).

Notably, the results concerning the effects of chromium on the female reproductive system are far from unambiguous. The National Toxicology Program report from 2008 did not document any statistically significant variations in reproductive characteristics among female rodents (NTP 2008). Furthermore, research on animals treated with varying doses of chromium for periods ranging from 3 months to 2 years has demonstrated that neither the male reproductive system nor the concentration or motility of sperm are impacted by Cr(VI) (NTP 2008).

#### Chromium and cancer

Hexavalent chromium compounds are substances known to cause cancer in humans. This is supported by a substantial body of research showing that exposure to chrome in the chromium alloy, chrome manufacturing, plating, and pigment industries can cause cancer in people. In addition to other cancers, hexavalent chromium has been associated with occupational exposure to lung cancer (Gibb et al. 2000).

A comprehensive meta-analysis evaluating the relationships between exposure to Cr(VI) and the mortality and incidence of human cancers was recently published (Deng et al. 2019). In total, 44 cohort studies were included, with 94,089 cases; a total of 2938 patients died from respiratory system cancers. Cr(VI) exposure was correlated with a high risk of respiratory system cancer mortality (standardized mortality ratio (SMR) = 1.33; 95% confidence interval (CI) 1.19–1.48).

A growing body of evidence suggests that dysregulating pathways linked to stem cells may be a potential mechanism behind the carcinogenesis of hexavalent chromium. After being exposed to a continuous 1 μM hexavalent chromium exposure, immunosuppressed mice exhibited increased cellular proliferation, anchorage-independent growth, and tumor formation capacity in the lung epithelial cell line BEAS-2B (He et al. 2013). In addition to tumor growth and angiogenesis, hexavalent chromium-transformed cells also presented a 35-fold decrease in the expression of miR-143, a crucial regulator of IGF-IR/IRS1 signaling (He et al. 2013).

Chromates are frequent water contaminants in many regions of the world. Although statistically limited, an epidemiological trial conducted in China revealed that areas with increased concentrations of chromates in drinking water reported increased stomach cancer mortality (Zhang and Li 1987).

In the National Toxicology Program report in 2008, a 2-year rodent study of the oral intake of chromates such as sodium dichromate dihydrate in drinking water confirmed an association between gastrointestinal cancer and ingested Cr(VI) (NTP 2008). Sodium dichromate has been confirmed to induce neoplasms of the oral cavity and small intestine in rats and mice, respectively. Conversely, no conclusive evidence of carcinogenicity/toxicity was found after exposure to Cr(III) as chromium picolinate in feed. Unlike Cr(III), which has significant limitations in crossing the cell membrane, Cr(VI) easily enters cells through nonspecific anion channels. While the intracellular reduction of Cr(VI) to Cr(III), which mostly occurs in the stomach, is believed to be a cause of genotoxicity and carcinogenicity, extracellular reduction is considered to be a detoxifying mechanism. Based on the tissue distribution study of male rats and female mice, it was revealed that the highest concentrations of chromium were detected in tissues after exposure to Cr(VI). Significantly lower concentrations of chromium were detected in controls and after Cr(III) exposure at an equivalent external dose. This implies that a small quantity of Cr(VI) species spread systemically throughout the body after escaping gastrointestinal reduction (Witt et al. 2013).

Regarding the possible mechanism of carcinogenesis, low levels of Cr(VI) exposure from drinking water may trigger the formation of high ROS levels, which in turn may help to induce the formation of tumors due to DNA mutations, increasing cell proliferation, altering the epigenome, preventing cell death, and accelerating tumor growth (Shin et al. 2023).

Taken together, compared with respiratory intoxication, oral exposure, due to the extracellular reduction of Cr(VI) to Cr(III) in the gastrointestinal tract, results in less Cr(VI) entering target cells. However, some of the ingested Cr(VI) may escape extracellular reduction in the stomach and instead enter cells. Even a small amount of Cr(VI) that penetrates cells has the capacity to trigger the development of tumors via various mechanisms. Chromium levels in drinking water in risk areas must, therefore, be set at levels that safeguard the general public (Katsas et al. 2024).

#### Detoxification of heavy metals by chelation

Chelation therapy appears to be a suitable intravenous or oral therapy to chelate heavy metals in the blood, which are further eliminated from the body. Acute intoxication by heavy metals requires immediate medical help.

Biological systems contain various low-molecular-weight organic molecules (e.g., antioxidants, amino acids, and small peptides) with effective metal-chelating properties. Metals, often with an oxidation number of + 2, such as Pb^2+^, Cd^2+^, and Hg^2+^ are bound with chelating molecules by coordination bonds (Sears 2013).

As discussed above, cadmium, arsenic, lead, and mercury have no essential biochemical functions but have various toxic effects. Chelators can coordinate with metal ions through their electron donor atoms, such as nitrogen, oxygen, and sulfur. The strength of the metal ion–chelate interactions, characterized by the equilibrium constants, depends on several factors, such as the electronic configuration of the metal ions, the stereochemistry around the metal ions, and the nature of the chemical bond between the metal and donor atoms. Chelators form in the blood metal chelate structures and mobilize metals from tissues to the kidneys for excretion in the urine and/or to the liver by the bile.

**Fig. 9:**
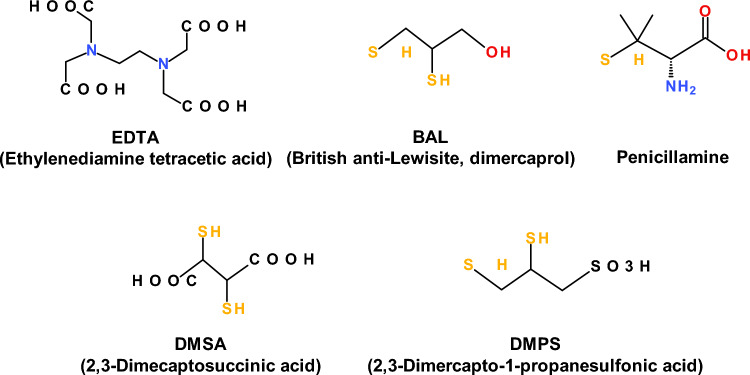
Selection of frequently used metal chelators

The most frequently used chelators for the treatment of heavy metal and metalloid intoxication are ethylenediaminetetraacetic acid (EDTA), dimercaprol, British anti-Lewisite (BAL), 2,3-dimercaptosuccinic acid (DMSA), 2,3-dimercapto-1-propane sulfonic acid (DMPS), and D-penicillamine (Fig. 9).

*Ethylenediaminetetraacetic acid (EDTA) *is approved by the U.S. Food and Drug Administration for treating lead poisoning in adults and children. In addition, EDTA has the ability to bind other heavy metals, such as zinc, cadmium, mercury, and iron (George and Brady 2023). Following interactions with metals, soluble metal‒EDTA complexes can be excreted from the body. EDTA is administered either intravenously or intramuscularly. Orally, EDTA is very poorly absorbed (< 5%). The intravenous administration of EDTA is effective, and EDTA is rapidly absorbed and transported into the plasma and interstitial fluids. EDTA has a charge of -4, which prevents its transport across cell membranes. The most profound adverse effect of EDTA is renal toxicity, which clinically manifests as renal failure, proteinuria, and other pathologies.

*BAL (dimercaprol)* is an intramuscularly administered chelator used to treat acute poisoning caused by mercury, arsenic, lead, and gold. Side effects include fever, gastrointestinal problems, and hypertension. In addition to its ability to treat heavy metal toxicity, dimercaprol has been approved by the FDA for the treatment of genetic disorders associated with copper homeostasis (Wilson’s disease).

*DMSA (2,3-dimercapto succinic aid)* is effective in the urinary excretion of cadmium, arsenic, lead, and inorganic and methyl mercury (Aposhian 1983). The DMSA is administered orally, intravenously, transdermally, or rectally in the clinic. DMSA is one of the most efficient chelating agents to remove methylmercury from various organ tissues, including animal brains.

*DMPS (2,3-dimercapto-1-propane sulfonic acid)* is a suitable chelator for cadmium, arsenic, and both organic and inorganic mercury (Aposhian 1983). The DMPS is preferentially administered intravenously, rapidly transformed into a disulfide form, and excreted in the urine and bile.

*D-Penicillamine* is an efficient copper removal used to treat Wilson’s disease. Penicillamine can also mobilize cadmium, arsenic, mercury, and lead (Eidelman and Lowry 2017). It is less efficient than DMSA and DMPS in removing methylmercury from animals; moreover, its removal efficiency from the brain is limited.

## Conclusion

A detailed analysis of many published papers revealed that anthropogenic (human) activities contribute significantly to environmental contamination by heavy metals such as aluminum, cadmium, arsenic, mercury, lead, and chromium (Mitra et al. 2022).

These and other heavy metals are recognized systemic toxicants that are associated with a number of human health disorders, including chronic diseases such as cardiovascular diseases, neurological disorders, renal, lung, and dermal diseases, and various types of cancer. Humans can be exposed to heavy metals not only in the natural environment where they live but also in different industries. The application of these principles and methodologies in managing chemical and biological hazards in the workplace is known as occupational toxicology.

Heavy metals enter organisms via three main routes: skin, inhalation, and ingestion. The pathway of metal exposure, time of exposure, and dose determine the potential health risk and degree of damage. The size of the dose usually determines whether it is chronic or acute metal intoxication. The degree of metal toxicity depends on various factors, including the valence state, the oxidation/reduction capacity of a metal ion, its solubility, the ionic radius of the metal, its lipophilicity/hydrophilicity, its ability to interact with the ligand environment, and other structural characteristics. The common denominators of all these processes include ROS-induced oxidative stress, dysregulation of antioxidant mechanisms, and inactivation of enzymes. Even though each heavy metal has a specific mechanism of toxicity, there are common unifying toxicity characteristics for certain groups of heavy metals outlined below.

*Heavy metal-induced ROS generation:* Several heavy metals have similar mechanisms of action. Arsenic, cadmium, mercury, lead, and chromium can preferentially interact with thiol groups (–SH) of antioxidant enzymes (e.g., SOD, catalase, GPx) and antioxidants (e.g., GSH), inhibit their activity, and impair mitochondrial function by increasing ROS production. In addition, increased ROS formation can alter the DNA repair process, induce DNA damage, and cause chronic diseases, including cancer. The mechanism of the carcinogenicity of heavy metals is thought to be related to defects in DNA repair following metal-induced oxidative stress and DNA damage. Toxic metal-induced ROS formation can induce epigenetic instability, which may be implicated in altered gene expression. Increased ROS formation is also responsible for the peroxidation of lipids, altered membrane fluidity, and the formation of malondialdehyde (MDA) and hydroxynonenal (HNE). Indirect cadmium-mediated ROS formation is due to the replacement of iron or copper by cadmium from metal-binding proteins. Accumulated iron/copper can catalyze the formation of ROS (e.g., by the Fenton reaction). The replacement of redox-active metals with cadmium can disrupt the biological metabolism of a cell.

*Heavy metal-induced cancer: *The carcinogenic effects of heavy metals related to ROS formation are outlined above. The carcinogenic effect of heavy metals has also been associated with their ability to interact with regulatory proteins involved in cell cycle regulation, DNA synthesis and repair, and the processes of apoptosis and necrosis. Cadmium and arsenic interfere with the activity of various transcription factors, including nuclear factor kappa B (NF-κB), activator protein-1 (AP-1), and p53. This may lead to failure of the expression of protective genes and consequently dysregulation of cell growth control. Cadmium can activate the transcription factors jun and fos and ERK1/2. A permanently activated signaling pathway causes continually activated proliferation, leading to increased tumor growth. It has been demonstrated that some heavy metals, such as cadmium, can increase the resistance of prostate epithelial cells to undergo malignant transformation via the overexpression of antiapoptotic Bcl-2 and the interruption of several proapoptotic mechanisms, such as the JNK pathway. In addition, chromosomal instability caused by defective DNA repair has been reported as an important mechanism for Cr^6+^-induced carcinogenesis.

**Fig. 10:**
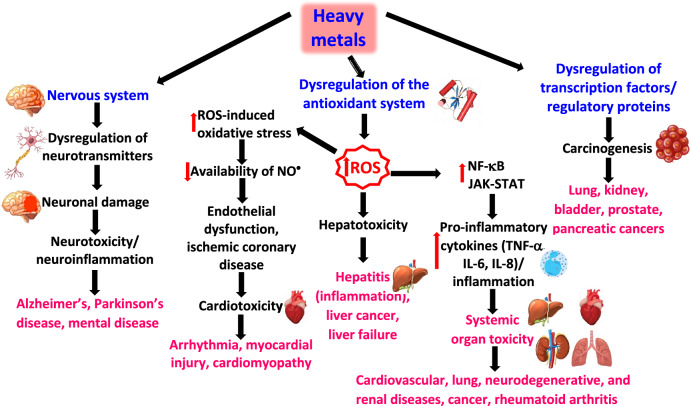
A simplified view of the mechanism of heavy metal toxicity (modified from Mitra et al. 2022)

*Heavy metal-induced epigenetic alterations:* Heavy metals can induce epigenetic modifications that impact gene activity and subsequent protein expression without changing the DNA sequence. Epigenetic alterations caused by lead, arsenic, mercury, and chromium involve DNA methylation and histone modification. A typical epigenetic alteration associated with cadmium is exclusively DNA methylation. At present, the exact mechanism by which heavy metal exposure/environmental factors induce epigenetic modifications is not known. However, a common denominator of this complex mechanism is the generation of ROS, which are responsible for the increased expression of protooncogenes and the silencing of tumor suppressor genes. For example, a well-known epigenetic change, DNA methylation, has been associated with inhibited expression of tumor suppressor genes. The carcinogenic effects of arsenic and cadmium have been attributed to epigenetic modifications involving DNA methylation and histone modification marks. After exposure to hexavalent chromium, human bronchial epithelial cells exhibit epigenetic dysregulation, such as increased levels of histone methyltransferases and histone H3 methylation marks. A simplified scheme of heavy metal toxicity in humans is shown in Fig. 10.

Occupational toxicology often has to solve the problems of intoxication with several metals at the same time; therefore, toxicology research into the different heavy metals that can interact within the body is very important. In addition, toxic metals can interact with essential metals in the body, and their mutual action can be additive, synergistic, or antagonistic. This and other problems surrounding the toxicity of heavy metals and their health effects require an interdisciplinary approach involving specialists in toxicology, pharmacology, epidemiology, molecular biology, and other disciplines.
